# Neglected medium-term and long-term consequences of labour and childbirth: a systematic analysis of the burden, recommended practices, and a way forward

**DOI:** 10.1016/S2214-109X(23)00454-0

**Published:** 2023-12-06

**Authors:** Joshua P Vogel, Jenny Jung, Tina Lavin, Grace Simpson, Dvora Kluwgant, Edgardo Abalos, Virginia Diaz, Soo Downe, Veronique Filippi, Ioannis Gallos, Hadiza Galadanci, Geetanjali Katageri, Caroline S E Homer, G Justus Hofmeyr, Tippawan Liabsuetrakul, Imran O Morhason-Bello, Alfred Osoti, João Paulo Souza, Ranee Thakar, Shakila Thangaratinam, Olufemi T Oladapo

**Affiliations:** a Maternal, Child and Adolescent Health Program, Burnet Institute, Melbourne, VIC, Australia; b UNDP–UNFPA–UNICEF–WHO–World Bank Special Programme of Research, Development and Research Training in Human Reproduction (HRP), Department of Sexual and Reproductive Health and Research, World Health Organization, Geneva, Switzerland; c Centro de Estudios de Estado y Sociedad (CEDES), Buenos Aires, Argentina; d Centro Rosarino de Estudios Perinatales (CREP), Rosario, Argentina; e School of Nursing and Midwifery, University of Central Lancashire, Preston, UK; f Department of Infectious Disease Epidemiology, London School of Hygiene & Tropical Medicine, London, UK; g Africa Center of Excellence for Population Health and Policy, Bayero University, Kano, Nigeria; h S Nijalingappa Medical College and HSK Hospital & Research Centre, Bagalkot, India; i Department of Obstetrics and Gynaecology, University of Botswana, Gaborone, Botswana; j University of the Witwatersrand and Walter Sisulu University, East London, South Africa; k Department of Epidemiology and Department of Obstetrics and Gynecology, Faculty of Medicine, Prince of Songkla University, Hat Yai, Thailand; l Department of Obstetrics and Gynaecology, Faculty of Clinical Sciences and Institute for Advanced Medical Research and Training, College of Medicine, University of Ibadan, Ibadan, Nigeria; m Department of Obstetrics and Gynaecology, University of Nairobi, Nairobi, Kenya; n Department of Social Medicine, Ribeirao Preto Medical School, University of São Paulo, São Paulo, Brazil; o Croydon University Hospital, London, UK; p Institute of Metabolism and Systems Research, University of Birmingham, Birmingham, UK

## Abstract

Over the past three decades, substantial progress has been made in reducing maternal mortality worldwide. However, the historical focus on mortality reduction has been accompanied by comparative neglect of labour and birth complications that can emerge or persist months or years postnatally. This paper addresses these overlooked conditions, arguing that their absence from the global health agenda and national action plans has led to the misconception that they are uncommon or unimportant. The historical limitation of postnatal care services to the 6 weeks after birth is also a contributing factor. We reviewed epidemiological data on medium-term and long-term complications arising from labour and childbirth beyond 6 weeks, along with high-quality clinical guidelines for their prevention, identification, and treatment. We explore the complex interplay of human evolution, maternal physiology, and inherent predispositions that contribute to these complications. We offer actionable recommendations to change the current trajectories of these neglected conditions and help achieve the targets of Sustainable Development Goal 3. This paper is the third in a Series of four papers about maternal health in the perinatal period and beyond.

This is the third in a **Series** of four papers about maternal health in the perinatal period and beyond, to be published in conjunction with *eClinical Medicine*. All papers in the Series are available at www.thelancet.com/series/maternal-perinatal-health

## Introduction

Accelerating the reduction of the maternal mortality ratio (MMR) has been a singular focus of the maternal health community for decades. This focus is reflected in more than 30 years of international initiatives, strategies, research prioritisation exercises, and quality improvement activities,^1^, ^2^, ^3^, ^4^, ^5^, ^6^, ^7^ as well as being the primary goal of countless regional, national, and local maternal health programmes worldwide.^8^, ^9^, ^10^ Many such programmes have prioritised intervention strategies to combat acute obstetric emergencies, such as haemorrhage, hypertensive disorders of pregnancy, and sepsis. Although these efforts have collectively helped to drive the global MMR to its lowest level in recorded history,^11^, ^12^ many of the medium-term and long-term (and often chronic) complications, which emerge after 6 weeks following childbirth, are comparatively less visible, or completely ignored. Conditions such as depression, urinary and anal incontinence, and sexual dysfunction can be caused or exacerbated by pregnancy and childbirth but might only present months, or even years, after childbirth. By this time, women are no longer accessing postpartum care services; historically, these services are only available up to 6 weeks after birth. These conditions can have lifelong social, economic, and health consequences. The situation can be worse in many low-income and middle-income countries (LMICs), particularly those with a persistently high burden of maternal mortality, than in high-income countries (HICs). Even in countries where resources are channelled to meet the global targets for the 2030 Sustainable Development Goals (SDGs) agenda, these long-term conditions are often overlooked. And even in settings where individuals present to health services with birth-related complications after postpartum care has ended, attending health providers might be ill-equipped to address these chronic conditions. Midwifery and obstetric training tends to focus on the management of the leading, acute causes of maternal morbidity and mortality.

One such complication—obstetric fistula—has benefited from a dedicated stakeholder community, resulting in high-profile advocacy, research, guidelines, and high-quality improvement initiatives.^13^, ^14^ Although more still needs to be done to alleviate the suffering of women affected by obstetric fistula, other fairly common complications that affect women in the medium term and long term after birth are rarely acknowledged or prioritised in the global health landscape. A scarcity of data on these conditions also impairs their visibility to key stakeholders. Neglect in the maternal health agenda translates into low visibility, financing, and collective effort to address these conditions at global, regional, and national levels.

In this third paper in the Series on maternal health in the perinatal period and beyond, we explore the spectrum of medium-term and long-term complications that can arise from the processes of labour and childbirth. We highlight the interplay between human evolution, maternal physiology, and inherent predisposition to these complications. We systematically searched for epidemiological data on these conditions, and systematically identified and summarised high-quality clinical guidelines on their prevention, identification, and treatment. We placed particular emphasis on the implications for women living in LMICs.

**Key messages**

• Many women experience labour-related and childbirth-related morbidity in the medium-to-long term after childbirth (ie, beyond 6 weeks postnatally). Available data show the most prevalent conditions are dyspareunia (35%), low back pain (32%), urinary incontinence (8–31%), anxiety (9–24%), anal incontinence (19%), depression (11–17%), tokophobia (6–15%), perineal pain (11%), and secondary infertility (11%).
• Other conditions that occur as a consequence of labour and childbirth are less frequent (or less common), yet still have severe effects on women's health and wellbeing. These conditions include pelvic organ prolapse, post-traumatic stress disorder, thyroid dysfunction, mastitis, HIV seroconversion, nerve injury, psychosis, venous thromboembolism, and peripartum cardiomyopathy.
• Most prevalence data on these conditions come from high-income countries with well resourced health systems. There are scarce population-level data from low-income and middle-income countries, except for postpartum depression, anxiety, and psychosis.
• Although high-quality guidelines do exist for some of these conditions, these are mostly developed in, and tailored for, high-income country settings. Available guidelines consistently highlight the importance of good-quality care at birth, systematic clinical assessments, screening of postpartum women to identify those at risk, and prompt management.
• To comprehensively address these conditions, broader and more comprehensive health service opportunities are needed, which should extend beyond 6 weeks postpartum and embrace multidisciplinary models of care. This approach can ensure that these conditions are promptly identified and given the attention that they deserve.

## Neglected medium-term and long-term complications arising from labour and childbirth

We conducted scoping literature searches and we developed a list of priority health conditions (panel). Eligible conditions were those affecting women that are directly or primarily related to the effects of labour and childbirth. This list includes conditions that are linked to, or exacerbated by, intrapartum interventions. We focused on those conditions emerging more than 6 weeks postpartum (with no upper time limit). The term neglected refers to those conditions that have been overlooked or underappreciated in national and global health agendas, as determined by consultation with the authorship group.

Panel **Medium-term and long-term conditions that can arise following labour and childbirth**

**Conditions related to labour and childbirth**

*Genitourinary conditions*

• Fistula (any type)
• Pelvic floor disorders, including pelvic organ prolapse (anterior or posterior vaginal wall prolapse); anal incontinence; urinary incontinence
• Wound complications*
• Secondary infertility

*Sexual health conditions*

• Postpartum sexual dysfunction (including dyspareunia)

*Mental health conditions*

• Postpartum depression
• Anxiety disorders
• Birth-related post-traumatic stress disorder
• Postpartum psychosis
• Tokophobia

*Cardiovascular conditions*

• Peripartum cardiomyopathy

*Neurological conditions*

• Neuropathies
• Neural injury

*Endocrine conditions*

• Postpartum thyroiditis

*Breast conditions*

• Mastitis (including recurrent or chronic)

*Metabolic conditions*

• Postpartum weight retention

*Infections*

• HIV seroconversion
• Sepsis (excluding sepsis within 6 weeks postpartum)

*Haematological conditions*

• Chronic anaemia†

*Effects on subsequent pregnancy*

• Placenta praevia or accreta
• Placental abruption
• Uterine rupture

**Conditions related to labour and childbirth interventions (ie, iatrogenic conditions)**

*Conditions specific to caesarean section, laparotomy, hysterectomy, or uterine rupture repair*

• Surgical-site infection
• Pelvic adhesions
• Bowel obstruction
• Surgical injury
• Uterine rupture
• Wound, scar, or uterine complications
• Deep vein thrombosis
• Pneumonia
• Pain and nerve injury
• Menorrhagia and dysmenorrhoea (often as a result of surgical intervention)
• Chronic pain
• Dyspareunia
• Secondary infertility
• Urinary incontinence; anal incontinence (often as a result of or worsened by surgical intervention)
• Pelvic organ prolapse (often as a result of or worsened by surgical intervention)
• Adverse psychological effects
• Adverse effects on subsequent pregnancies, including placental defects

*Conditions related to episiotomy or perineal repair*

• Perineal pain
• Urinary incontinence; anal incontinence
• Infections, injuries, or poorly healed perineum
• Dyspareunia

*Conditions related to operative vaginal birth*

• Pelvic organ prolapse
• Urinary incontinence; anal incontinence

These conditions can affect any person who has given birth, regardless of parity or mode of birth. These conditions can also emerge following use of intrapartum interventions, such as caesarean section, instrumental vaginal birth, episiotomy, hysterectomy, or laparotomy. We excluded studies on conditions that occur primarily in the short-term (within 6 weeks after birth), although we acknowledge that some medium-term to long-term conditions can develop within, but manifest beyond, this period. We excluded conditions that arise directly from comorbidities existing before pregnancy or that develop during pregnancy, such as diabetes or pre-eclampsia. We also excluded conditions affecting the child who has been born of the pregnancy, as they were outside the scope of this Series. In this paper, we refer to birthing parents as women in keeping with our review search terms, but we acknowledge that this group also includes gender-diverse people, as well as adolescent girls.

## Why do these complications occur?

**Figure 1:**
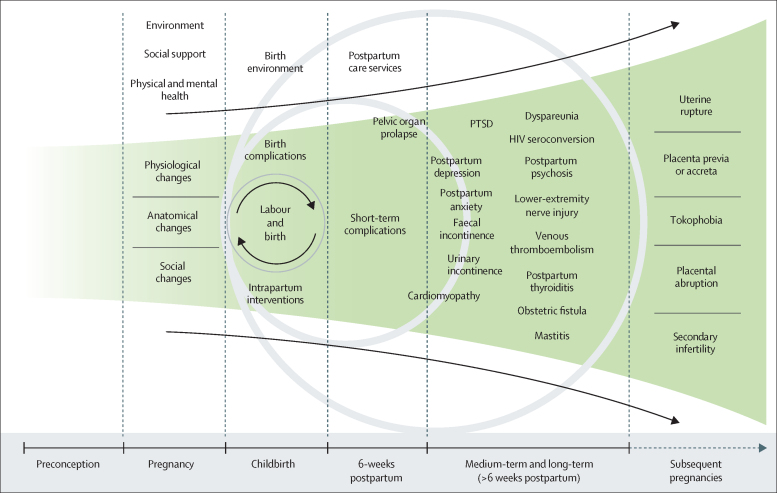
Schematic of the medium-term and long-term conditions that arise from labour and childbirth PTSD=post-traumatic stress disorder.

The majority of the approximately 140 million women who give birth globally every year do not have morbidity in the 6 weeks after birth. However, many women, even if they have an uncomplicated vaginal birth without the need for interventions, will have a postpartum complication. Substantial postpartum morbidity resulting from a physiological process, such as labour and birth, might seem counterintuitive. Figure 1 presents a conceptual representation of the interplay between predispositions that are intrinsic and extrinsic to pregnancy, labour, and childbirth, and medium-term and long-term maternal complications.

From an evolutionary standpoint, it is unsurprising that profound maternal adaptations that favour newborn survival have developed at the potential expense of long-term maternal health and wellbeing. In simplistic terms, many evolutionary adaptations will favour the fetus that carries genetic permutations to the next generation, rather than the wellbeing of the mother. The human brain consumes an extraordinary quantity of energy, requiring a relatively higher blood flow than that of non-human brains.^15^ Human brain evolution has depended upon development of an exceptionally efficient maternal–fetal exchange interface, the placenta.^16^, ^17^, ^18^ Because brain development, and consequently brain size, is privileged, human infants are immature at birth compared with other species, and are dependent on their family for years of physical and cognitive development. Human brain evolution has also resulted in a tight or disproportional fetopelvic fit. This feature is reflected in the observation that human births more frequently result in difficult labours due to fetal head–pelvic disproportion, as compared with other primates.^19^

The physiological changes of pregnancy affect multiple maternal organ systems, including the cardiovascular system (eg, spiral arteriole remodelling, reduced peripheral resistance, increased cardiac output and blood volume, and reduced blood pressure), immune system, endocrine and hormonal changes (altering hormone cycles and increased progesterone and oestrogen levels), and ligament laxity.^20^, ^21^, ^22^, ^23^, ^24^ Although these changes occur naturally during pregnancy (eg, relaxation of the pelvic floor ligaments and musculature for easier passage of the fetal head), they also increase the propensity for long-term complications to develop (eg, pelvic organ prolapse). These risks can be compounded by social and clinical factors (eg, poor social support, older or younger maternal age, parity, obesity, or comorbidities), fetal factors (eg, position or size), or whether intrapartum complications occurred (eg, malpresentation, prolonged second stage of labour, levator ani avulsion, or soft tissue trauma). Effective and timely care around the time of birth and a positive birth experience can thus prevent or reduce the severity of medium-term and long-term complications.

Although many labour and childbirth interventions are offered to minimise harm for mother or baby, their misuse or overuse can lead to iatrogenic complications.^25^ Episiotomy is a key example: routine episiotomy remains prevalent, although randomised trials have long shown that restrictive episiotomy policies, rather than liberal or routine use, are associated with less posterior perineal trauma and fewer complications.^26^ Similarly, the injudicious use of uterotonics to augment weak contractions during labour is a well known risk factor for life-threatening complications, such as uterine rupture. Women who survive uterine rupture can have devastating consequences, such as secondary infertility due to uterine wall repair and tubal ligation, partial or total hysterectomy, or pelvic sepsis. Even when pharmacological (eg, oxytocin or misoprostol for labour induction), mechanical (eg, instrumental vaginal birth), and surgical (eg, episiotomy or caesarean section) interventions are justified, they can still interfere with a woman's recovery. These complications can trigger adverse physical, social, or psychological outcomes that can persist or emerge long after childbirth.

The context of birth affects a woman's natural response to labour and childbirth. For example, birth in an unfamiliar, unsupportive, or hostile clinical environment has been found to affect a woman's emotional adjustment to parenthood and is associated with adverse clinical and emotional outcomes in the long term, such as a woman's sense of competence, confidence in adapting to parenthood, and the initiation of breastfeeding.^27^, ^28^ Sleep deprivation, difficulty breastfeeding, low support in parenting, and childcare-related challenges can also increase the risk of postpartum depression.^29^ Broader determinants of maternal health as presented in the first paper^30^ in this Series—the physical environment and climate, food security, housing, clean water, sanitation, employment, and adverse family, social, or financial circumstances, among others—can further accentuate risks and worsen postpartum outcomes.^31^

Much of the long-term morbidity following childbirth relates to mechanical injury. Vaginal birth involves considerable stretching of soft tissues in the pelvic floor, anal canal, and those supporting the bladder and urethra.^32^ This stretching can result in levator ani muscle injury—women with levator ani avulsion have greater risks of symptomatic prolapse.^33^, ^34^ Even in the absence of overt perineal tears, ultrasound can reveal separation or disruption of pelvic muscle fibres in some women.^35^ These clinical features can result in tissue laxity over the longer term, leading to pelvic organ prolapse or incontinence.^36^ Although this process can happen in pregnant women with an uncomplicated vaginal birth, studies have shown that forceps delivery, despite being protective for the fetus, is associated with greater maternal tissue damage.^26^ Softening of the symphysis pubis and sacroiliac joints during pregnancy can also lead to longer-term symphyseal or pelvic girdle pain.^37^

As depicted in figure 1, an important concept underpinning the interplay between the factors described earlier and labour and childbirth-related complications is time as an effect modifier. Both the intrinsic and extrinsic factors that make women susceptible to developing these complications tend to increase with labour duration. For example, the likelihood of interventions during labour increases as the first or second stages of labour become atypically long. An understanding of this relationship is fundamental to developing and taking preventive measures to reduce the burden of chronic conditions arising from the events surrounding labour and childbirth.

## The global burden of neglected medium-term and long-term conditions after labour and childbirth

### Identifying data on the burden of these conditions

We systematically searched for the best available estimates of the prevalence or incidence of adverse conditions occurring in the long term after labour and childbirth (appendix pp 2–4). Reliable global or national estimates were our preferred data source; however, no such estimates existed for any condition. We assessed review quality using the AMSTAR tool (A MeaSurement Tool to Assess systematic Reviews),^38^ a 16-point checklist that categorises a review as high, medium, low, or critically low quality, based on its methodology.

We identified 53 data sources—seven were representative household surveys or large maternity registers and 46 were systematic reviews. Two (4%) reviews were high quality, six (13%) were moderate quality, 12 (26%) were low quality, and 26 (57%) were critically low quality. The table summarises the identified data (appendix pp 29–37). For many conditions, differences in estimated prevalence were probably affected by different measurement tools, as well as whether population-based or facility-based sampling was used. Estimates from LMICs tended to rely on facility-based sampling studies, which are more likely to produce biased results. Although some conditions, such as urinary incontinence, postpartum depression and anxiety, and chronic pain had multiple data sources, we were unable to identify any eligible data for several conditions.

**Table:** Summary of epidemiological data on medium-term and long-term conditions after labour and childbirth

	Evidence base*
**Genitourinary conditions**	
Fistula (any type)	Household surveys in 19 sub-Saharan African countries suggest the lifetime prevalence of symptoms of vaginal fistula is 3·0 cases per 1000 women of reproductive age (95% CI 1·36–5·5).^39^ Lifetime prevalence of fistula symptoms among women who ever had a livebirth ranged from 3·4% in Uganda to 5·8% in Malawi.^40^ A systematic review found that the incidence of obstetric fistula in LMICs ranged from 0 to 4·09 cases per 1000 births.^41^ A separate systematic review, which included ten studies and 34 505 women, reported a pooled prevalence of obstetric fistula in LMICs of 0·29 per 1000 women (95% CI 0·00–1·07).^42^
Pelvic organ prolapse	Three US surveys conducted between 2005 and 2010 reported that the weighted prevalence of pelvic organ prolapse increased with parity, ranging from 1·4% to 4·5%.^43^ Two systematic reviews reported pelvic organ prolapse after vaginal birth occurred in 6%^44^ and 13·7%^45^ of women.
Anal incontinence	Three US surveys reported that the weighted prevalence of faecal incontinence increased with parity, ranging from 8·3% to 11·8%.^43^ A systematic review (25 studies including 2523 women) found that, among women who gave birth vaginally, the pooled prevalence of anal sphincter defect on ultrasound was 26% (95% CI 21–30) and of anal incontinence symptoms was 19% (14–25).^46^ A separate systematic review reported that faecal incontinence^47^ affected 27% of women at 6 weeks to 1 year postpartum, 33% of women between 2 years and 5 years postpartum, and 26% of women at over 5 years postpartum.
Urinary incontinence	Three US surveys reported that the weighted prevalence of moderate-to-severe urinary incontinence increased with parity, ranging from 11·8% to 23·9% (6112 women).^43^ A systematic review reported that the mean (weighted) prevalence of urinary incontinence in postpartum women between 6 weeks and 12 months in HICs was 31% (95% CI 26–36; 24 studies, 24 064 women), and the prevalence ranged between 10% and 63%.^48^ Other systematic reviews estimated the prevalence of stress urinary incontinence at 7·9%,^45^ 12–19%,^49^ 13·3%,^50^ and 15%.^44^ One systematic review reported a prevalence of stress urinary incontinence in women with levator ani muscle avulsion of 29–69%, at least 1 year after childbirth.^51^
**Sexual and reproductive health conditions**	
Secondary infertility	An analysis of 277 household surveys estimated that the prevalence of secondary infertility was 10·5% (95% CI 9·5–11·7).^52^
Postpartum sexual dysfunction (including dyspareunia)	A systematic review reported that the overall prevalence of dyspareunia was 35% (95% CI 29–41).^53^ In total, 43% of individuals had dyspareunia from 2 to 6 months postpartum (36–50), 22% (15–29) from 6 to 12 months postpartum, and 40% (33–47) from 12 to 24 months postpartum (one study). This review also reported that 89% (83–93) of women had resumed sexual intercourse within 2 to 6 months postpartum and 99% (97–100) within 6 to 12 months.^53^ A separate systematic review reported that 82–90% of women resumed sexual intercourse between 6 months and 9 months postpartum, and at least one problem was reported in 36–43% of women at 6 months and 80–91% of women at 12 months.^54^ Another review reported that the pooled prevalence of dyspareunia for women with an intact perineum following spontaneous vaginal birth was 15% (5–44) at 6 months postpartum and 16% (8–32) at 12 months postpartum.^55^
**Mental health conditions**	
Postpartum depression	A systematic review reported a pooled prevalence of postpartum depression of 14% (95% CI 12–15).^56^ These authors also reported a postpartum depression prevalence of 12·9% at 8 weeks postpartum, 17·4% (5·9–28·9) at 12 weeks postpartum, and 13·6% (10·4–16·8) at 24 weeks postpartum. Prevalence varied by country economic level, with 17·0% (14–20) in developing countries and 11·0% (8·8–13·2) in developed countries (according to terminology of the study). Four other systematic reviews reported on pooled prevalence of postpartum depression and reported findings of: 18·7% (17·8–19·7) in LMICs and 9·5% (8·9–10·1) in HICs;^57^ 17·7% (16·6–18·8) between 4 weeks and 52 weeks postpartum, ranging from 3·1% to 37·7%;^58^ 16% (13–19) at 4–6 months, 20% (11–29) at 7–12 months, and 25% (12–39) at over 12 months postpartum in women without a previous history of depression;^59^ and 15·9% (14–18) at 3–6 months, 17·9% (13–24) at 6–12 months, and 17·9% (14–23) at over 12 months postpartum.^60^
PTSD	A systematic review reported the pooled prevalence of PTSD was 1·1% (95% CI 0·5–2·0).^61^ A separate systematic review reported a pooled prevalence of PTSD of 1·4% (0·3–6·5) at 3 months and 6·8% (1·6–24·5) at 6 months postpartum.^62^ A third systematic review reported the rate of postpartum PTSD at 3 to less than 6 months postpartum was between 3·4% and 6·7%.^63^
Postpartum psychosis	A systematic review identified one study that reported prevalence of postpartum psychosis in the USA of 5 in 1000 women.^64^ A separate systematic review (five studies in LMICs) reported a cumulative incidence of postpartum psychosis of 1·1 per 1000 births, and the prevalence between studies ranged from 1·1 to 16·7 per 1000 births.^65^
Anxiety disorders	A systematic review reported that the pooled prevalence of postpartum anxiety disorder was 16% (95% CI 13·5–18·9) and of self-reported anxiety symptoms was 24·4% (16·2–33·7).^66^ A separate systematic review reported the pooled prevalence of postpartum anxiety disorder was 9·6% (3·4–15·9) at 5–12 weeks postpartum, 9·9% (6·1–13·8) at 1–24 weeks postpartum, and 9·3% (5·5–13·1) at over 24 weeks postpartum.^67^ A third systematic review reported the pooled prevalence of postpartum generalised anxiety disorder was 2·6% (1·3–3·8).^61^
Secondary tokophobia	A systematic review reported prevalence rates of fear of childbirth varied from 6·3% to 14·8% (as defined by a Wijma delivery expectancy/experience questionnaire score ≥85) in ten studies across seven European countries.^68^ A separate systematic review (including 17 studies) reported the pooled prevalence of tokophobia was 14% (95%CI 12–16) overall, and 12% (10–14) among multiparous women.^69^
**Cardiovascular conditions**	
Peripartum cardiomyopathy	An analysis of the National Inpatient Sample database in the USA reported the prevalence of peripartum cardiomyopathy as 33·5 per 100 000 livebirths in women aged 15–35 years, and 77·6 per 100 000 livebirths in women aged 36–54 years.^70^ A systematic review reported that the incidence of peripartum cardiomyopathy varied between countries, with the highest rates in Nigeria (1 in 102 births) and lowest in Japan (1 in 15 533 births).^71^
Venous thromboembolism	A systematic review reported the pooled incidence rate of venous thromboembolism during pregnancy and puerperium was 1·4 per 1000 (1·0–1·8 per 1000), and the authors estimated that 57·5% (95% CI 20·9–63·9) of these venous thromboembolism events occur postpartum (rather than antepartum).^72^
**Neurological conditions**	
Neuropathies and neural injury	A systematic review reported that the incidence of lower extremity nerve injury up to 6 months postpartum ranged from 0·3% to 2·3%.^73^
Chronic pain	A systematic review reported the incidence of first-onset low back pain ranged from 19% to 53%, with a mean incidence of 31·6%.^74^ A separate systematic review reported an incidence of perineal pain of 11% for women with an intact perineum following spontaneous vaginal birth at 3 months postpartum (95% CI 0·01–100).^55^
**Endocrine conditions**	
Postpartum thyroiditis	A systematic review reported the pooled prevalence of postpartum thyroid dysfunction was 8·1% (95% CI 7·8–8·2) and ranged from 0·9% to 11·7%. The authors also reported a prevalence of 7·9% (7·7–8·1) at up to 6 months postpartum, 6·4% (6·3–6·5) at up to 9 months postpartum, and 9·2% (9·1–9·3) at up to 12 months postpartum.^75^
**Breast conditions**	
Mastitis	A systematic review reported the pooled incidence rate of lactational mastitis at 0–25 weeks postpartum was 11·1 episodes per 1000 breastfeeding weeks (95% CI 10·2–12·0). Prevalence ranged from 2·5% to 20%.^76^
**Infections**	
HIV seroconversion	A systematic review reported the pooled HIV incidence rate was 2·9 per 100 person-years postpartum (95% CI 1·8–4·0).^77^
**Effects on subsequent pregnancy**	
Placenta previa or accrete	A systematic review reported the increased absolute risk of placenta accreta following caesarean section.^78^ The absolute risk was: 3·3 per 10 000 for no caesarean section; 12·9 per 10 000 after one caesarean section; 41·3 per 10 000 after two caesarean sections; 78·3 per 10 000 after three caesarean sections; 217 per 10 000 for four caesarean sections; and 230 per 10 000 for five or more caesarean sections.
Placental abruption	A systematic review reported the incidence of abruption ranged from 0·01% to 5·1%.^79^
Uterine rupture	An analysis of INOSS data (nine European countries) reported the prevalence of complete uterine rupture was 3·3 per 10 000 births (95% CI 3·1–3·5). Prevalence varied widely between countries, from 1·6 to 7·8 per 10 000 births.^80^
**Conditions specific to caesarean section, laparotomy, or hysterectomy**	
Uterine and wound complications	A systematic review reported the pooled incidence of chronic wound pain after caesarean section was 15·4% (95% CI 9·9–20·9) at 3–6 months postpartum; 11·5% (8·1–15·0) at 6–11 months postpartum; and 11·2% (7·4–15·0) at 12 months or more postpartum.^81^
Uterine rupture	An analysis of INOSS data (nine European countries) reported the overall prevalence of complete uterine rupture after caesarean section was: 22 per 10 000 births (95% CI 21–24) in women with previous caesarean section, and 35 per 10 000 births (32–37) in women with previous caesarean section and labour.^80^ Prevalence varied widely between countries, ranging from 8 to 68 per 10 000 births in women with previous caesarean section, and from 16 to 80 per 10 000 births in women with previous caesarean section and labour. A WHO multicountry survey analysis reported the incidence of uterine rupture in women who have had a caesarean section was 45 per 10 000 births, ranging from 0·1% to 2·5%.^80^ A systematic review reported the absolute risk of uterine rupture in vaginal birth after caesarean section was 0·87% and after planned repeat caesarean section was 0·09%. Uterine rupture rates ranged from 0% to 1·69%.^82^
Scar complications	A systematic review reported the prevalence of caesarean section scar defect ranged between 24% and 88% on ultrasonography.^83^ A separate systematic review reported the prevalence of a niche (caesarean section scar defect) in women with a history of caesarean section varied between 56–84% using contrast-enhanced sonohysterography and 24–70% using transvaginal sonography.^84^
Venous thromboembolism	A systematic review reported the pooled incidence of venous thromboembolism following caesarean section was 2·6 per 1000 caesarean section births (95% CI 1·7–3·5).^85^ In studies with a longer and better postpartum follow-up, incidence increased to 4·3 per 1000 caesarean section births (1·0–4·3).
Pain, nerve injury, chronic pain	A systematic review reported the incidence of chronic pain following a caesarean section at 2 to 6 months postpartum ranged between 4% and 41·8% (17 studies).^86^ A separate systematic review reported the pooled incidence of chronic postsurgical pain 3 to 6 months following a caesarean section was 15·4% (95% CI 9·9–20·9).^81^ A third systematic review reported the pooled prevalence of chronic pain following a caesarean section was: 19% (12–23) at 2 to 5 months, 13% (9–17) at 6 to 11 months, and 8% (6–10) at 12 months or more.^87^ A fourth systematic review reported pelvic pain after caesarean section occurred in 1·3% of women.^44^
Secondary infertility	A systematic review reported secondary infertility among women with caesarean section delivery was 43%.^44^
Pelvic organ prolapse	Analysis of three USA surveys reported the weighted prevalence of pelvic organ prolapse among women who had caesarean delivery only was 1·9% (95% CI 1·1–3·3). The weighted prevalence among women with previous hysterectomy was 5·4% (4·0–7·3).^43^ A systematic review reported that pelvic organ prolapse after caesarean section occurred in 21·2% of women, and stress urinary incontinence in 10·2% of women.^45^ A separate systematic review reported pelvic organ prolapse after caesarean section was 2·3%.^44^
Urinary incontinence	Analysis of three USA surveys reported the weighted prevalence of urinary incontinence among women who had caesarean section was 12·7% (95% CI 10·4–15·4). The weighted prevalence among women with previous hysterectomy was 29·5% (26·8–32·3).^43^ A systematic review reported the absolute prevalence of stress urinary incontinence among women who had had one or more caesarean sections ranged from 5% to 15%.^49^ A separate systematic review reported that urinary incontinence after caesarean section occurred in 14% of women.^44^ All studies reported caesarean delivery had lower rates of urinary incontinence compared with vaginal delivery.^43^, ^44^, ^49^
Anal incontinence	Analysis of three USA surveys reported the weighted prevalence of faecal incontinence among women who had a caesarean section was 6·4% (95% CI 4·6–8·8). The weighted prevalence among 1717 women with previous hysterectomy was 16·6% (14·6–18·8).^43^ A systematic review reported the rate of faecal incontinence after caesarean section was 3·6%.^44^ One study reported caesarean delivery had lower rate of anal incontinence than vaginal birth,^43^ but one study reported no statistically significant difference in rates.^44^
PTSD	A systematic review reported the pooled prevalence of PTSD after caesarean section was 9·0% (95% CI 4·6–15·6) at more than 8 weeks postpartum, and 4·8% at 4 weeks to more than 12 months postpartum (2·0–9·7).^88^
Adverse effect on subsequent pregnancies	A systematic review reported that the absolute risk of previa associated with caesarean section was 12 per 1000 deliveries (95% CI 8–15). The overall incidence of abruption with any previous caesarean section was 1·2–1·5%, and the rate of abruption was 10·3–15·0 per 1000 deliveries. The reported incidence of placenta accreta ranged from 11% in women with one previous caesarean section to 67% in women with 5 or more previous caesarean section deliveries.^89^ A separate systematic review reported the increased absolute risk of placenta accreta following caesarean section was 3·3 per 10 000 for no caesarean section, 12·9 per 10 000 after one caesarean section, 41·3 per 10 000 after two caesarean sections, 78·3 per 10 000 after three caesarean sections, 217 per 10 000 for four caesarean sections, and 230 per 10 000 for five or more caesarean sections.^78^
**Conditions related to episiotomy or perineal repair**	
Incontinence	A systematic review reported the prevalence of urinary incontinence ranged from 0·7% to 29·1%, and anal incontinence ranged from 2·8% to 29·1% among women with episiotomy.^90^ A separate systematic review reported the prevalence of urinary incontinence ranged from 0% to 75·2%.^91^
Postpartum sexual dysfunction	A systematic review reported the prevalence of sexual dysfunction ranged from 7·9% to 64·9%.^90^ A separate systematic review reported the incidence of dyspareunia for women with a repaired second-degree tear or episiotomy following spontaneous vaginal birth was 16% (95% CI 2–100) at 6–7 weeks postpartum, and 19% (13–28) at 3 months postpartum.

For several conditions, no systematic reviews of prevalence were identified: cervical incompetence, wound complications, Grave's disease, Sheehan's syndrome, postpartum weight retention, abnormal placentation, chronic anaemia, some post-procedural complications (surgical site infection, pelvic adhesions, bowel obstruction, surgical injury, pneumonia, menorrhagia, dysmenorrhea, sexual dysfunction), some post-episiotomy complications (poorly healing perineum or perineal pain after episiotomy), and conditions related to operative vaginal birth. HIC=high-income country. INOSS=The International Network of Obstetric Survey Systems. LMIC=low-income and middle-income country. PTSD=post-traumatic stress disorder.

* When confidence intervals were available in the support evidence base, they were reported; however, several reviews did not provide confidence intervals.

### Adverse conditions affecting more than 10% of women in the medium term or long term after birth

Conditions affecting more than 10% of women in the medium-term or long-term postpartum period (beyond 6 weeks after birth) include dyspareunia (difficult or painful sexual intercourse); anal or urinary incontinence, or both; postpartum depression; tokophobia (severe fear of childbirth); and chronic postpartum pain, such as low back pain and perineal pain.

A high-quality review reported that 35% (95% CI 29–41) of postpartum women have dyspareunia, which varied by time since birth: 43% of women had dyspareunia from 2 months to 6 months postpartum, 22% from 6 months to 12 months, and 40% from 12 months to 24 months.^53^ Among women who gave birth vaginally, the pooled prevalence of anal sphincter defect on ultrasound was 26% (21–30), whereas 19% (14–25) of women had anal incontinence symptoms at over 1 year after delivery.^46^ This study only assessed women following vaginal delivery; we also searched for other studies reporting prevalence on obstetric anal sphincter injury after caesarean section or instrumental vaginal delivery, but were unable to find reliable data. We identified several moderate-quality reviews for postpartum urinary incontinence from 6 weeks after delivery, with prevalence estimates of 8%,^45^ 12–19%,^49^ 13·3%,^50^ and 31%.^48^ The National Health and Nutritional Examination Survey (NHANES) of 6112 women in the USA found that faecal incontinence (prevalence ranging from 8% to 12%) and moderate-to-severe urinary incontinence (ranging from 12% to 24%) increased with parity.^43^ Factors including increasing age, some racial and ethnic groups, low educational status, high BMI, high number of comorbidities, and hysterectomy were associated with higher prevalence of incontinence.

Postpartum depression varied between HICs, which have a prevalence of 11% (95% CI 9–13), and LMICs, which have a prevalence of 17% (14–20).^56^ Three other reviews (of low quality or critically low quality) compared postpartum depression prevalence by country-income category. All of these reviews reported a higher prevalence of postpartum depression for women in LMICs (from 17·0% to 20·1%) compared with those in HICs (from 9·5% to 15·5%).^56^, ^58^, ^60^ Other reviews also found variation in postpartum depression prevalence between countries, and over time since birth.^57^, ^58^, ^59^, ^60^ A high-quality review found that the pooled prevalence of postpartum anxiety disorder was 16% (95% CI 13·5–18·9), whereas 24·4% (16·2–33·7) of women had self-reported anxiety symptoms.^66^ A separate, moderate-quality review reported that the pooled prevalence of postpartum anxiety disorder seemed stable over time since birth, with 9·6% (3·4–15·9) prevalence at 5–12 weeks postpartum, 9·9% (6·1–13·8) at 1–24 weeks, and 9·3% (5·5–13·1) beyond 24 weeks.^67^ Other than country income level, the latter two studies did not identify any additional mediating factors.

A moderate-quality systematic review identified ten studies from seven European countries reporting that the prevalence of postpartum fear of childbirth varied from 6·3% to 14·8%.^68^ A separate review found tokophobia was present in 12% (95% CI 10–14) of women, although there was substantial heterogeneity between studies.^69^ Neither of these studies identified any mediating factors or had timeframe limitations.

An analysis of 277 household surveys estimated that the prevalence of secondary infertility—defined as the inability to have a subsequent livebirth after a previous birth—was 10·5% (9·5–11·7).^52^ We identified two critically low-quality reviews on postpartum pain conditions—one reported that the mean incidence of first-onset low back pain from postpartum up to menopausal age was 31·6% (range 19–53).^74^ The second found the incidence of perineal pain among women with an intact perineum following spontaneous vaginal birth was 11% (95% CI 1–100) at 3 months postpartum, although we note the wide confidence interval of this finding.^55^

### Adverse conditions affecting 1% to less than 10% of women in the medium term or long term after birth

Conditions affecting 1% to less than 10% of women include pelvic organ prolapse, birth-related postpartum post-traumatic stress disorder (PTSD), postpartum thyroid dysfunction, lactational mastitis, HIV seroconversion, and lower-extremity nerve injury. Three US surveys reported that the weighted prevalence rates of pelvic organ prolapse increased with parity, ranging from 1·4% to 4·5%.^43^ Two additional reviews estimated that pelvic organ prolapse occurred in 6·0%^44^ and 13·7%^45^ of women after vaginal birth, and 2·4%^44^ and 21·2%^45^ after caesarean delivery. Increasing age, some racial and ethnic groups, low education status, high BMI, various comorbidities, and hysterectomy were associated with increasing incidence of pelvic floor disorders.

A systematic review reported a 1·1% (95% CI 0·5–2·0) prevalence of postpartum PTSD,^61^ whereas another review found prevalences of 1·4% (0·3–6·5) at 3 months and 6·8% (1·6–24·5) at 6 months.^62^ A third review reported that postpartum PTSD at 3 to less than 6 months postpartum was between 3·4% and 6·7%.^63^ Studies reported that comorbidities, such as depression, history of psychological problems or trauma, emergency caesarean section, pregnancy complications, and low levels of social support were risk factors for postpartum PTSD.^62^, ^63^ The pooled prevalence of postpartum thyroid dysfunction was 8·1% (7·8–8·2), although this varied from 7·9% (7·7–8·1) at up to 6 months postpartum, 6·4% (6·3–6·5) at up to 9 months, and 9·2% (9·1–9·3) at up to 12 months; no data were presented after 12 months postpartum and no mediating factors were identified.^75^ We identified one, critically low-quality review that reported on lactational mastitis.^76^ This review reported that at 0–25 weeks postpartum, 11·1 (95% CI 10·2–12·0) episodes of mastitis occurred per 1000 breastfeeding weeks, and prevalence from 0–12 months postpartum ranged from 2·5% to 20% across studies.^76^ Nipple damage or nipple pain, and infection with *Staphylococcus aureus* were reported risk factors for mastitis. Similarly, a critically low-quality review on HIV seroconversion found that the pooled HIV incidence rate was 2·9 (1·8–4·0) per 100 person-years postpartum.^77^ Studies identified pregnancy-induced physiological changes, sexually transmitted infections and other genital tract infections, vitamin A deficiency, severe anaemia, and younger age (<20 years) as potential risk factors for HIV incidence. The incidence of lower extremity nerve injury up to 6 months postpartum ranges from 0·3% to 2·3%, and occurred more often in women's first births and when neuraxial (epidural) analgesia or anaesthesia was used.^73^

### Adverse conditions affecting less than 1% of women in the medium term or long term after birth

Conditions affecting less than 1% of women include postpartum psychosis, venous thromboembolism, peripartum cardiomyopathy, and obstetric fistula. A moderate-quality review reported that the cumulative incidence of postpartum psychosis up to 1 year postpartum ranged from 1·1 to 16·7 per 1000 births, and did not report any mediating factors.^65^ A low-quality review found the pooled incidence rate of venous thromboembolism during pregnancy and puerperium was 1·4 per 1000 women (95% CI 1·0–1·8), of which 57·5% (20·9–63·9) occurred postpartum; no specific timeframe or mediating factors influencing incidence were reported in this review.^72^

An analysis of the National Inpatient Sample database in the USA reported that the prevalence of peripartum cardiomyopathy was 33·5 per 100 000 livebirths in women aged 15–35 years, and 77·6 per 100 000 livebirths in women aged 36–54 years.^70^ The incidence differed between racial and ethnic groups, with highest incidence in Black groups, followed by White and Hispanic groups. A systematic review found that the incidence of peripartum cardiomyopathy varied between countries, with the highest rates in Nigeria (1 in 102 births) and the lowest in Japan (1 in 15 533 births).^71^, ^77^

There were conflicting estimates on obstetric fistula prevalence. A meta-analysis of household surveys from 19 sub-Saharan African countries suggested that the lifetime prevalence of symptoms of vaginal fistula was 3·0 cases per 1000 women of reproductive age (95% CI 1·4–5·5).^39^ A separate analysis of three household surveys on obstetric fistula symptoms among women who have had a livebirth found considerably higher rates than the previous meta-analysis, ranging from 3·4% in Uganda to 5·8% in Malawi.^41^ Other systematic reviews, focused on women in LMICs, reported obstetric fistula prevalence ranging from 0 to 4·09 cases per 1000 births,^41^ to 0·29 cases per 1000 women (0·00–1·07).^42^ None of these studies identified mediating factors influencing incidence of obstetric fistula, other than country.

## Clinical guidelines for the prevention, recognition, and treatment of medium-term and long-term morbidities arising from labour and childbirth

Guidelines support clinicians on what interventions they should (or should not) offer and are ideally informed by a systematic assessment of available evidence.^92^ We conducted a systematic search to identify high-quality guidelines on the prevention, screening, diagnosis, and treatment of the adverse conditions of interest, from 2010 onwards (appendix pp 18–20). Because guidelines use different terminology, we extracted and classified all recommendations on the basis of their directions, regardless of the grading recommendations and levels (hierarchy) of evidence or recommendation strength. We did not critically appraise the evidence base for individual recommendations. We identified 157 guidelines, of which 46 were high-quality (appendix pp 41–42).^93^

46 high-quality guidelines were published between March, 2012, and March 30, 2022 by 72 different organisations (appendix pp 41–42). They pertained to 19 of the conditions that we selected, with most issued from HICs (the UK, the USA, European countries, Canada, and Australia). None were developed by an LMIC-based organisation. However, seven were WHO guidelines recommended for use internationally, which were developed with the involvement experts of various disciplines and from a range of countries (including LMICs). These guidelines covered postpartum depression (n=10), urinary incontinence (n=9), uterine and wound complications (n=8), and postpartum anxiety (n=7). There were few high-quality guidelines for other conditions, such as fistula, mastitis, and postpartum thyroiditis. No high-quality guidelines were identified for several conditions, such as secondary infertility and neuropathies. We extracted 898 recommendations from these 46 guidelines, which were categorised based on type (appendix pp 50–71).

**Figure 2:**
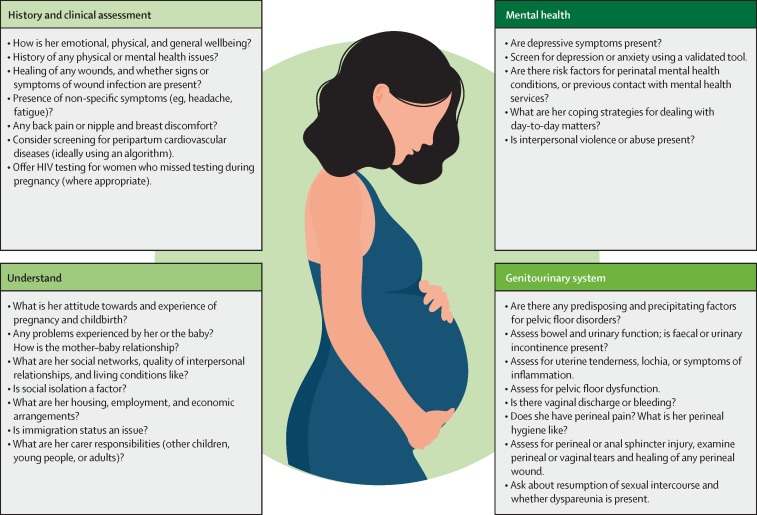
Recommended practices and actions for health providers, in order to identify postpartum women who are at risk of developing or currently have a medium-term or long-term condition

A key theme across the guidelines was the need for providers to consider a broad range of functional, emotional, behavioural, sexual, and other quality-of-life issues affecting women in the postpartum period. Several guidelines recommended systematic clinical assessments or formal screening using validated tools to identify those who have or are at risk of adverse conditions. We synthesised recommendations relating to clinical assessment and screening into a simplified list to help providers detect or exclude these conditions (figure 2). These practices are relevant to providers working in postpartum care, as well as those working in other health-care services in which they encounter women beyond the standard 6-week postpartum period. Opportunities to identify these conditions as early as possible should not be missed, so treatment can be commenced promptly. An overarching message is that timely, women-centred, and evidence-based care is the most effective preventive (or reparative) strategy for many complications.

## How can we improve the prevention, recognition, and treatment of medium-term and long-term conditions arising from labour and childbirth?

There is a scarcity of comprehensive evidence regarding the prevalence of these conditions. Many reviews had methodological gaps, which resulted in low or critically low-quality assessments using the AGREE-II tool. Authors of included reviews noted the challenges in pooling prevalence data from studies that used different clinical definitions, diagnostic tools, and sampling strategies, leading to greater uncertainty in pooled estimates. Although we sought prevalence estimates specific to LMICs, few reviews had sufficient data in this regard; thus, the burden of medium-term and long-term conditions in LMICs remains largely unknown. Even where LMIC-based data were available, there is an over-reliance on facility-based studies with non-representative sampling. These knowledge gaps highlight the need for robust, high-quality, population-based prevalence studies in LMICs to guide prioritisation and decision-making.

Although we found high-quality clinical guidelines for many conditions, a major concern was the paucity of guidelines developed within, or for, LMIC contexts specifically. Although the paucity of guidelines does not reflect the health-care provider's awareness of these conditions and their clinical practice, it is nonetheless concerning. We hypothesise that in many countries these long-term complications are underappreciated, under-recognised, and under-reported. Developing high-quality, evidence-based guidelines requires substantial resources and methodological expertise. Efforts to adapt existing guidelines to low-resource settings, using tools such as ADAPTE, can be useful.^94^ However, guidelines alone are insufficient to improve clinical practice and women's health outcomes—they need to be accompanied by proactive knowledge transfer strategies.^92^

One key theme from the guideline review was that women in the postpartum period might face a range of psychological, emotional, and physical challenges. These challenges can widely affect a woman's health and wellbeing, and that of her family. Supporting women in navigating these challenges requires a flexible and responsive postpartum care model—one that is person-centred and respectful, but also multidisciplinary, with integration of diverse health services. A model like this would ensure that women who have or are at risk of adverse conditions are identified as early as possible. Effective prevention of medium-term and long-term complications demands the provision of high-quality, timely, and evidence-based care during pregnancy, childbirth, and the early postpartum period. This care should allow the risky extremes of the so-called too much, too soon and too little, too late situations to be avoided.^25^

Formal postpartum care services have historically focused on the 6-week period after birth, reflecting the official WHO definition of the postpartum period of up to 42 days post-birth.^95^ However, this timeframe is not fit for purpose. The heightened risks from childbirth do not end at this somewhat arbitrary timepoint—they can persist up to and beyond 1 year after birth. Gazeley and colleagues found that in 12 sub-Saharan African countries, the risk of mortality in postpartum women was still 20% higher at 42 days to 4 months postpartum, as compared with the baseline risk of 12–17 months postpartum.^96^ Kassebaum and colleagues estimated that 35·6% of maternal deaths occurred between 24 h and 42 days postpartum, yet a further 12·1% occurred between 43 days and 1 year postpartum.^97^ A multitude of childbirth-related conditions, many of considerable prevalence, will first present after 42 days postpartum. Women who have antenatal complications, such as gestational diabetes or pre-eclampsia, are at an increased risk of non-communicable diseases in later life, such as diabetes and cardiovascular disease, and thus require additional preventive care into later life. Acknowledging that the coverage of postnatal care in many countries is poor,^98^ current postnatal care models are not adequately oriented to the timing and diversity of postpartum-related challenges that women face.^99^ Given the diverse nature of the conditions that we have identified, there is a need for more and better integration of postnatal care services with other disciplines to ensure that women have access to primary care providers, mental health services, or other relevant specialties, as required.

## Research priorities for medium-term and long-term conditions after labour and childbirth

A literature search identified ten research prioritisation activities for only a few of the adverse conditions we considered. These conditions included obstetric fistula,^100^, ^101^ faecal incontinence,^102^, ^103^ urinary incontinence,^104^ maternal mental health,^105^ peripartum cardiomyopathy,^106^ HIV in women in the postpartum period,^107^ and postpartum infections;^108^, ^109^ and a WHO-led prioritisation that captured a few other long-term conditions.^5^ This inconsistent approach reflects the absence of a comprehensive research agenda that addresses the full range of medium-term and long-term consequences after childbirth.

A key finding from our review is that more and better measurement of these conditions is urgently required. Although data suggest that some conditions might be more prevalent in LMICs, the scale of the burden in these countries is not fully known. Representative prevalence data need to be gathered and reported in a standardised way to be useful to clinicians, stakeholders, and policy makers. Converging on standardised measurement tools and study designs that ensure prevalence data are reliable, comparable, and representative is necessary.^110^ Epidemiological research to understand drivers, risk factors, and rates of medium-term and long-term complications; why rates differ across settings; and the effects of these complications on women's quality of life and wellbeing are also needed. An overarching methodological consideration is the need for population-based, rather than facility-based, sampling. Facility-based samples are typically composed of women who have access to care or have elected to seek it, and as such are not representative of all women in the postpartum period. This problem is more pronounced in settings where women's access to health services is poor. Conditions such as anal incontinence, urinary incontinence, postpartum depression and anxiety, and sexual dysfunction can be difficult for women and health-care providers to talk about; careful consideration is required to determine how these conditions can be accurately ascertained and reported.

Interventional research is needed on how we can most effectively prevent or reduce the effects of these conditions, particularly in LMIC settings. Implementation research is also required to understand how effective practices can be operationalised, and at what cost. Many of the guidelines that we identified recommend multidisciplinary models of care, special investigations, and interventions that require substantial health resources. Development and testing of novel, responsive, and person-centred models of postnatal care that can work for women in resource-limited settings is long overdue. Although the consideration of economic evidence was outside the scope of our analysis, it is probable that these conditions impose a considerable (and often avoidable) cost burden on health systems and societies. Economic evaluations are therefore crucial to attract attention and generate meaningful policy change around these conditions.

## A call to action

In conclusion, we call for greater recognition, improved measurements, and collective action and funding to prevent and manage medium-term and long-term consequences of labour and childbirth, many of which affect millions of women worldwide. These conditions are not mainstream in the global agenda or national health action plans of many countries, meaning that they are not prioritised from a public health perspective. This neglect has led to an unfounded misperception that these conditions are uncommon or unimportant. However, the trajectory of a woman's long-term health, wellbeing, and quality of life is shaped by her experiences during labour and childbirth, and the quality of care she received at that time. Efforts to reach universal health coverage need to address how these medium-term and long-term conditions after childbirth can be better prevented, identified, and treated. Widespread awareness-raising is required, and lessons need to be learned from advocacy success stories, such as the International Day to End Obstetric Fistula. The success of fistula-related advocacy includes framing fistula as a health and human rights issue,^111^ SDG target-setting,^112^ generating prevalence data and global estimates,^39^, ^42^ implementing national policy initiatives^113^ and norms and standards from international agencies,^13^, ^114^, ^115^ and multilateral advocacy at local, national, regional, and global levels.^15^

Several measures can be taken immediately to improve this situation. First, policy makers, clinicians, and those who manage health services can revisit their current care arrangements. They can ask questions such as: which part of the health service is responsible for the prevention and management of childbirth-related conditions, and how can the needs of women in the postpartum period be better met? How can postpartum care be extended or optimised to identify these conditions, and to better link with different disciplines and community-based services? How can the health system response and care availability for these conditions be strengthened? Second, it is important to amplify the voices of women, representative organisations, patient advocacy, and other community-led groups that are drawing attention to these conditions, often without the support of other maternity or women's health stakeholders. Third, greater investment is needed in epidemiological, interventional, and implementation research, as well as evidence-based guidelines for these conditions, to drive positive change. This research should engage with, and be driven by, key stakeholders in affected communities. Fourth, women and families should have access to evidence-based information on prevalence, prevention, and management of medium-term and long-term complications of childbirth. Fifth, postpartum period definitions as a threshold for postnatal care services should be reconsidered, as should the period beyond 42 days. Last, but not least, countries should prioritise the development and implementation of health policies that address the full range of health conditions that tend to emerge long after the time of birth, but which are traceable to labour and childbirth processes and events.

## Search strategy and selection criteria

To identify epidemiological data or estimates on prevalence, we did a comprehensive search of UN agency websites, population-representative household survey datasets, national and international maternity registry datasets, as well as PubMed, Embase, CINAHL, and Epistemonikos for systematic reviews. The search was conducted from Jan 1, 2000, to April 12, 2022. We searched for studies with several variations of the primary key search terms “pregnancy” or “childbirth” or “postnatal”, and “prevalence”, and terms related to the conditions of interest. We used a hierarchical search strategy to identify the best available estimate or evidence of the burden (ie, prevalence or incidence), with a specific focus on data for low-income and middle-income countries (appendix pp 2–4). The titles and abstracts of search results were independently screened and selected against prespecified eligibility criteria. When multiple systematic reviews were available, we prioritised by recency, their eligibility criteria and limitations, and population representativeness (appendix pp 2–4). For guidelines, we adopted the Institute of Medicine's definition of a guideline as “statements that include recommendations, intended to optimize patient care, that are informed by a systematic review of evidence and an assessment of the benefits and harms of alternative care options”. We first developed a list of 54 organisations producing guidelines that are used internationally for the conditions of interest and searched their websites directly for guidelines published since 2010. We also did a systematic search of MEDLINE, Trip database (Guideline filter function), and the Guideline International Network website (inception to May 10, 2022). Recovered citations were screened in duplicate. We used the AGREE-II instrument to identify high-quality guidelines. AGREE-II uses 23 criteria to assess the quality of a clinical practice guideline, on the basis of the guideline's scope, purpose, stakeholder involvement, rigour of development, clarity of presentation, applicability, and editorial independence. From the 46 high-quality guidelines, individual recommendations were extracted and analysed (appendix pp 50–71).

## Declaration of interests

We declare no competing interests.

## Footnotes

* Secondary to perineal tears, episiotomy, or caesarean section (see subsequent items).

† Risk of chronic anaemia might be higher in women following postpartum haemorrhage, anaemia during pregnancy, or other complications and conditions.
