# Supplementary material for: Neglected medium-term and long-term consequences of labour and childbirth: a systematic analysis of the burden, recommended practices, and a way forward
Source: Lancet Glob Health. 2023 Dec 6;12(2):e317–30. doi: 10.1016/S2214-109X(23)00454-0 (PMC10805007; doi:10.1016/S2214-109X(23)00454-0)
Supplement: Supplementary appendix [file mmc1.pdf]

THE LANCET
Global Health

Supplementary appendix

This appendix formed part of the original submission and has been peer reviewed. We post it as supplied by the authors.

Supplement to: Vogel JP, Jung J, Lavin T, et al. Neglected medium-term and long-term consequences of labour and childbirth: a systematic analysis of the burden, recommended practices, and a way forward. *Lancet Glob Health* 2023; published online Dec 6. https://doi.org/10.1016/S2214-109X(23)00454-0.

Table of Contents

Supplementary File S1. Methods including search strategy and eligibility criteria for epidemiological data on medium and long-term conditions of interest 2

Supplementary File S2. Methods related to systematic review of guidelines..................... 18

Supplementary Table S1. Epidemiological data on medium- and long-term conditions of interest 29

Supplementary Table S2. Summary of guidelines and recommendations on long-term conditions of interest 41

Supplementary Table S3. Recommended and not recommended practices for medium- and long-term conditions of interest 50

Supplementary File S1. Methods including search strategy and eligibility criteria for epidemiological data on medium and long-term conditions of interest

Defining the conditions of interest

The authorship team conducting scoping literature reviews in February 2022 to develop a list of medium and long-term conditions, which were refined through iterative discussions with the authorship group.

All conditions met the following criteria:

- Directly (or primarily) triggered or exacerbated by the process of labour and childbirth, or relate to the use of intrapartum interventions during labour and childbirth;
- Conditions that occur in the medium- to long-term postpartum period. This period was defined as starting from 6 weeks after birth[1], with no upper limit specified;
- These conditions may be related to spontaneous vaginal birth, instrumental vaginal birth or caesarean section, specific labour-related peripartum complications (such as perineal trauma), or related to the use of peripartum interventions in labour and childbirth (such as episiotomy, perineal repair, laparotomy, hysterectomy, uterine rupture repair, hysterectomy);
- Are neglected complications of labour and childbirth. By neglected, we mean a diverse group of health conditions that are:
    - prevalent in women after birth;
    - affect women who live in resource-limited settings;
    - have significant health, social and economic consequences; and
    - are absent from the global health agenda.

We excluded conditions that:

- Occurred during birth, but resolved within 6 weeks postpartum.[2] Hence, common, shorter-term complications (such as postpartum haemorrhage, postpartum eclampsia, peripartum sepsis and others) were excluded;
- Affected the newborn, infant or child only;
- Were directly or primarily related to co-morbid conditions or complications during pregnancy (rather than labour and childbirth itself);
- Are primarily related to adverse outcomes that occur during labour and childbirth (for example, conditions directly related to a severe maternal outcome, stillbirth, or similar).

[1] Women may experience signs or symptoms of some of these conditions within the first 6 weeks, that later continue into the medium or long-term (e.g. incontinence or postpartum depression) and these conditions are included.

Identifying available evidence on the burden of medium- and long-term conditions related to labour and childbirth

We aimed to identify the best available estimate or evidence of the burden (i.e. prevalence or incidence) of the specified medium and long-term conditions, with a focus on ensuring we captured data related to LMICs. To achieve this, a hierarchical search strategy was used:

1. We searched United Nations (UN) agency websites (WHO, World Bank, UNData and similar) to identify any global, regional, or national estimates since 2000 for each condition. If reliable estimates (including for low and middle-income countries) were available for a single condition, these were incorporated and reported.

2. We also searched websites of national- or international-level maternity registry datasets (such as USA, UK, European Health for All databases) and nationally or sub-nationally representative household survey datasets (Demographic and Health Surveys, Reproductive Health Surveys, Multiple Indicator Cluster Surveys[3]) for data on conditions of interest. If national-level or population-representative data were available for a given condition since 2000, these data were incorporated and reported.

3. Many conditions did not have any data identified from steps 1 and 2 above. Hence, we also searched PubMed, EMBASE, CINAHL and Epistemonikos using structured search terms to identify systematic reviews of prevalence or incidence published since 2000 on the condition of interest. The search strategy used synonyms for the condition of interest combined with search terms related to maternal health and prevalence/incidence, limited to 2000 onwards, and restricted to systematic reviews only (see end of this document for full search strategy). We did not include systematic reviews or studies that reported measures of association only (such as odds ratios or risk ratios). We also did not include systematic reviews that reported prevalence in non-representative samples such as highly specific/selected or at-risk populations (eg: migrant populations, groups of specific races, women with preterm infants only), nor did we include systematic reviews on the effects of interventions. To be considered a systematic review, the following features had to be present:
    - A clearly stated set of objectives with pre-defined eligibility criteria for the studies;
    - An explicit, reproducible methodology;
    - A systematic search of 2 or more scientific databases that attempts to identify all the studies that would meet the eligibility criteria;
    - An assessment of the validity of the findings of the included studies, for example through the assessment of the risk of bias or quality assessment of included studies;
    - A systematic presentation, and synthesis, of the characteristics and findings of the included studies.

4. If multiple systematic reviews were present for a single condition, we prioritised including those systematic reviews according to the following:

    - Systematic review's eligibility criteria considered data from LMICs to be eligible;

[3] We are aware that some medium and long term conditions (such as fistula, depression, secondary infertility and others) are reported in household surveys, but a number of other conditions of interest are not.

- Recency of the literature search date – more recent reviews were preferred;
- Minimal limitations to the time-span covered. Reviews that focused on a short time period, such as duration of the COVID-19 pandemic, were de-prioritised;
- Systematic review focused on conditions of interest and provided a clear definition
- The systematic review was framed at nationally or sub-nationally representative samples of women.

5. If no systematic reviews were identified for a condition, we conducted additional searches on Google and Google Scholar to ensure no eligible systematic reviews had been overlooked or not captured by the search strategy. If none were found, reviews that were previously excluded at full-text review - for not meeting systematic review criteria of quality assessment or having searched less than 2 databases - were included.

6. We then assessed the quality of included systematic reviews using a modified version[4] of AMSTAR (A MeaSurement Tool to Assess systematic Reviews).[1] AMSTAR assessments were performed independently by two reviewers and were categorised as high, medium, low or critically low quality.

7. We then assembled a database composed of the following:

   - Findings of any global, regional or national estimates on conditions of interest (#1 above)
   - Findings of any national-level or population-representative data on conditions of interest (#2 above)
   - Findings from any systematic reviews on conditions of interest (#3 to #6 above)

8. Findings were summarized narratively and reported.

A PRISMA flowchart summarizing the findings of above steps is provided below. A summary of modified AMSTAR assessments for each included review is also reported below.

[4] Selected AMSTAR Items were modified to improve suitability for systematic reviews of prevalence/incidence. The modifications included the following: Item 3 was met if authors provided an explanation of the selection of non-randomised study for interventions; the criteria 'included/consulted content experts in the field' was removed from Item 4; and for Item 7, providing the number and description of reasons for exclusion was deemed sufficient to meet the criteria 'provide a list of all potentially relevant studies that were read in full-text form but excluded from the review'.

Table S1. Search strategy for PubMed (searched 12 April 2022)

#	Query	Results
1	Pregnancy[MeSH] OR Pregnant Women[MeSH] OR Postpartum Period[MeSH] OR Maternal Health[MeSH] OR pregnant OR pregnancy OR postnatal OR postpartum OR maternal OR Parturition[MeSH] OR childbirth[MeSH] or birth OR delivery OR parturition OR "labor" OR "labour" OR puerperium OR obstetric OR deliver* OR caesarean OR mother*	2,598,596
2	Systematic[SB] OR Meta-Analysis[Publication Type] OR Cochrane Database Syst Rev[SO] OR "systematic review"[Title] OR "rapid review"[Title] OR "living review"[Title] OR "scoping review"[Title] OR "meta-analysis"[Title]	336,545
3	Prevalence[MeSH] OR Incidence[MeSH] OR Prevalence OR Incidence OR Burden	4,027,002
4	#1 AND #2 AND #3	15,901
5	Urinary Fistula[MeSH] OR Vaginal Fistula[MeSH] OR "urinary fistula" OR "vaginal fistula" OR "rectal fistula" OR "vesicovaginal fistula" OR "vesico-vaginal fistula" OR "rectovaginal fistula" OR "recto-vaginal fistula" OR "perianal fistula" OR "peri-anal fistula" OR "obstetric fistula" OR "urethrovaginal fistula" OR UVF OR "ureterovaginal fistula" OR "urogenital fistula" OR fistula OR "anovaginal fistula" OR "ano-vaginal fistula" OR VVF OR RVF	126,651
6	Pelvic Floor Disorders[MeSH] OR Pelvic Organ Prolapse[MeSH] OR Urinary Incontinence[MeSH] OR "pelvic floor disorder*" OR "pelvic organ prolapse" OR PFD OR POP OR cystocele OR enterocele OR "rectal prolapse" OR "uterine prolapse" OR "visceral prolapse" OR "vaginal prolapse" OR "anterior compartment prolapse" OR "posterior compartment prolapse" OR "apical compartment prolapse" OR "uterine procidentia" OR "vaginal wall prolapse" OR "genital prolapse" OR "urinary incontinen*" OR "faecal incontinen*" OR "fecal incontinen*" OR "anal incontinen*" OR "flatus incontinen*"	178,565
7	"Cervical insufficiency" OR "cervical incompetence" OR "incompetent cervix" OR "short cervix" OR "shortened cervix" OR "cervical weakness"	2,633
8	Wound Infection[MeSH] OR "wound complication" OR "wound morbidity" OR "wound infection" OR "wound breakdown" OR "wound dehiscence"	73,376
9	Infertility, Female[MeSH] OR "secondary infertility" OR subfertility	109,979
10	Dyspareunia[MeSH] OR Sexual Dysfunction, Physiological[MeSH] OR "sexual dysfunction" OR dyspareunia OR "sexual problem*" OR "sexual function" OR "sexual health" OR "sexual pain"	63,461
11	Depression, Postpartum[MeSH] or "postpartum depression" OR "post-partum depression" OR "postnatal depression" OR "post-natal depression" OR "Postpartum depressive disorder*" OR "post-partum depressive disorder*" OR "postpartum unipolar depression" OR "post-partum unipolar depression" OR "postnatal depressive disorder*" OR "post-natal depressive disorder*" OR "postnatal unipolar depression" OR "post-natal unipolar depression" OR PPD	21,755
12	Anxiety Disorders[MeSH] OR postpartum anxiety OR post-partum anxiety OR postnatal anxiety	91,796
13	Stress disorders, post traumatic[MeSH] OR PTSD OR "post-traumatic stress disorder*" OR "post traumatic stress disorder*" OR "posttraumatic stress disorder "	53,779
14	Psychotic disorders[MeSH] OR "postpartum psychosis" OR "post-partum psychosis" OR psychotic disorder* OR "postpartum psychoses" OR "post-partum psychoses"	74,239
15	Phobic Disorders[MeSH] OR tokophobia OR tocophobia	12,049
16	Cardiomyopathies[MeSH] OR Pregnancy Complications, Cardiovascular[MeSH] OR PPCM OR "peripartum cardiomyopath*" OR "peripartum cardiomyopath*" OR "postpartum cardiomyopath*" OR "post-partum cardiomyopath*"	120,349
17	Peripheral Nervous System Diseases[MeSH] OR Neuropath* OR "neural injur*" OR "nerve injur*" OR "lower extremity neuropath*" OR "lower extremity nerve injury" OR	324,218

	"femoral neuropath*" OR "peroneal neuropath*" OR "postpartum neuropath*" OR "post-partum neuropath*"	
18	Postpartum Thyroiditis[MeSH] OR Thyroid Diseases[MeSH] OR Hypopituitarism[MeSH] OR "postpartum thyroiditis" OR "post-partum thyroiditis" OR "grave's disease" OR "graves disease" OR "sheehan's syndrome" OR "sheehans syndrome" OR "postpartum thyroid" OR "post-partum thyroid"	168,513
19	Mastitis[MeSH] OR Lactation Disorders[MeSH] OR Breast Feeding/adverse effects[MeSH] OR mastitis	21,623
20	Gestational Weight Gain[MeSH] OR "weight retention" OR "weight gain" OR GWG	84,220
21	HIV Seropositivity[MeSH] OR Pregnancy Complications, Infectious/epidemiology[MeSH] OR Sepsis[MeSH] OR Puerperal Infection[MeSH] OR HIV seroconversion OR HIV seropositiv* OR sepsis OR septic OR septicaemia OR septicemia	283,578
22	Anemia, Iron-Deficiency/epidemiology [MeSH] OR Pregnancy Complications, Hematologic/blood[MeSH] OR Anemia/epidemiology[MeSH] OR Iron Deficiencies[MeSH] OR anemia OR anaemia	247,255
23	Placenta Previa/epidemiology[MeSH] OR Placenta Diseases/epidemiology[MeSH] OR "placenta previa" OR "placenta accrete" OR "placenta accreta" OR Uterine Rupture[MeSH] OR "uterine rupture" OR Abruptio Placentae/epidemiology[MeSH] OR "placental abruption" OR "abnormal placentation"	16,338
24	Postoperative Complications[MeSH] OR Obstetric Surgical Procedures/adverse effects[MeSH] OR Cesarean Section/adverse effects[MeSH] OR Vaginal Birth after Cesarean/adverse effects[MeSH] OR laparotomy/adverse effects[MeSH] OR Hysterectomy/adverse effects[MeSH] OR Intestinal Obstruction/epidemiology[MeSH] OR Intestinal Obstruction/complications[MeSH] OR Tissue Adhesions/epidemiology[MeSH] OR Tissue Adhesions/complications[MeSH] OR "bowel obstruction" OR "surgical injur*" OR Venous Thrombosis/epidemiology[MeSH] OR Venous Thromboembolism/epidemiology[MeSH] OR "deep vein thrombosis" OR DVT OR "venous thrombosis" OR Pneumonia/epidemiology[MeSH] OR pneumonia OR Chronic Pain/epidemiology[MeSH] or Pain Measurement[MeSH] OR "chronic pain" OR menorrhagia/epidemiology[MeSH] OR Dysmenorrhea/epidemiology[MeSH] OR menorrhagia OR dysmenorrhoea OR subsequent pregnanc*	1,223,933
25	Delivery, Obstetric/adverse effects [MeSH]	11,864
26	#5 OR #6 OR #7 OR #8 OR #9 OR #10 OR #11 OR #12 OR #13 OR #14 OR #15 OR #16 OR #17 OR #18 OR #19 OR #20 OR #21 OR #22 OR #23 OR #24 OR #25	2,974,675
27	#4 AND #26	4,558
28	#4 AND #26	3,241

Table 2. Search strategy for EMBASE (searched 12 April 2022)

#	Query	Results
1	exp pregnancy/ or pregnancy.mp. or pregnant women.mp. or exp pregnant woman/ or exp puerperium/ or postpartum.mp. or maternal health.mp. or exp postnatal care/ or postnatal.mp. or parturition.mp. or exp birth/ or exp childbirth/ or childbirth.mp. or birth.mp. or exp birth/ or labour.mp. or exp labor/ or obstetric.mp. or exp caesarean section/ or caesarean.mp. or mother.mp.	1,847,632
2	exp meta analysis/ or exp "systematic review"/ or (systematic review or metaanalysis or meta-analysis or meta analysis or rapid review or living review or scoping review).m_titl.	512,702
3	exp prevalence/ or prevalence.mp. or incidence.mp. or exp incidence/ or burden.mp. or exp disease burden/	2,894,941
4	1 and 2 and 3	7,576
5	exp urinary tract fistula/ or exp rectum fistula/ or exp cystovaginal fistula/ or exp rectovaginal fistula/ or exp anus fistula/ or (urinary fistula or vaginal fistula or rectal fistula or vesicovaginal fistula or vesico-vaginal fistula or rectovaginal fistula or recto-vaginal fistula or perianal fistula or peri-anal fistula or obstetric fistula or urethrovaginal fistula or UVF or ureterovaginal fistula or urogenital fistula or fistula or anovaginal fistula or ano-vaginal fistula or VVF or RVF).mp.	197,797
6	exp pelvic organ prolapse/ or exp urine incontinence/ or exp feces incontinence/ or exp anterior vaginal wall prolapse/ or exp urethral prolapse/ or exp rectum prolapse/ or exp uterus prolapse/ or exp posterior vaginal wall prolapse/ or exp rectum prolapse/ or exp pelvic floor disorder/ or exp rectocele/ or exp feces incontinence/ or (pelvic floor disorder or pelvic organ prolapse or POP or PFD or cystocele or vaginal prolapse or anterior compartment prolapse or posterior compartment prolapse or apical compartment prolapse or uterine procidentia or vaginal wall prolapse or genital prolapse or urinary incontinence or faecal incontinence or fecal incontinence or anal incontinence or flatulus incontinence).mp.	143,441
7	exp uterine cervix incompetence/ or (cervical insufficiency or cervical incompetence or incompetent cervix or short cervix or shortened cervix or cervical weakness).mp.	3,966
8	exp wound complication/ or exp wound infection/ or exp wound dehiscence/ or (wound infection or wound complication or wound morbidity or wound infection or wound breakdown or wound dehiscence).mp.	93,454
9	exp infertility/ep or (infertility or secondary infertility or subfertility).mp.	154,659
10	exp dyspareunia/ or exp female sexual dysfunction/ or exp sexual dysfunction/ or (dyspareunia or sexual dysfunction or sexual problem or sexual function or sexual health or sexual pain).mp.	134,564
11	exp postnatal depression/ep or exp postnatal depression/ep or (postpartum depression or post-partum depression or postnatal depression or post-natal depression or postpartum depressive disorder or post-partum depressive disorder or postpartum unipolar depression or post-partum unipolar depression or postnatal depressive disorder or post-natal depressive disorder or postnatal unipolar depression or post-natal unipolar depression or PPD).mp.	27,184
12	exp anxiety disorder/ep or exp postnatal depression/ep or (postpartum anxiety or post-partum anxiety or postnatal anxiety or post-natal anxiety).mp.	12,623
13	exp posttraumatic stress disorder/ep or (PTSD or post-traumatic stress disorder or posttraumatic stress disorder or post traumatic stress disorder).mp.	78,132
14	exp puerperal psychosis/ep or (postpartum psychosis or post-partum psychosis psychotic disorder* or postpartum psychoses or post-partum psychoses or puerperal psychosis).mp.	1,658
15	(tokophobia or fear of childbirth or tocophobia).mp. or exp "fear of childbirth"/	781
16	(peripartum cardiomyopath* or PPCM or peri-partum cardiomyopath* or postpartum cardiomyopath* or post-partum cardiomyopath*).mp. or exp peripartum cardiomyopathy/	3,054

17	(neuropath* or neural injur* or nerve injur* or lower extremity neuropath* or femoral neuropath* or peroneal neuropath* or postpartum neuropath* or post-partum neuropath*).mp. or exp neuropathy/ep or exp peroneal neuropathy/ep	426,874
18	(postpartum thyroiditis or post-partum thyroiditis or graves disease or sheehan*).mp. or exp postpartum thyroiditis/ep or exp Graves disease/ep	32,319
19	mastitis.mp. or exp mastitis/	20,760
20	(weight gain or weight retention or GWG).mp. or exp weight gain/ or exp gestational weight gain/	153,563
21	(HIV seropositivity or HIV seroconversion or HIV seropositive or sepsis or septicaemia or septicemia).mp. or exp Human immunodeficiency virus infection/ep or exp puerperal sepsis/ep or exp sepsis/ep [mp=title, abstract, heading word, drug trade name, original title, device manufacturer, drug manufacturer, device trade name, keyword heading word, floating subheading word, candidate term word]	340,175
22	exp anemia/ep or (anemia or anaemia).mp.	439,602
23	exp placenta previa/ep or exp placenta accreta/ep or exp placenta disorder/ep or exp solution placentae/ or exp uterus rupture/ep or (abnormal placentation or placenta previa or placenta accreta or placental abruption or abnormal placentation or uterine rupture).mp.	21,277
24	exp surgical infection/ep or exp peritoneum adhesion/ep or exp intestine obstruction/ or exp deep vein thrombosis/ or exp pneumonia/ep or exp menorrhagia/ or exp dysmenorrhea/ or exp chronic pain/ or exp vaginal delivery/co or exp caesarean section/co or exp laparotomy/co or exp hysterectomy/co or exp uterus rupture/co, su or (bowel obstruction or pelvic adhesion or tissue adhesion or surgical injury or surgical infection or venous thrombosis or deep vein thrombosis or pneumonia or chronic pain or menorrhagia or dysmenorrhoea or subsequent pregnanc*).mp.	772,432
25	5 or 6 or 7 or 8 or 9 or 10 or 11 or 12 or 13 or 14 or 15 or 16 or 17 or 18 or 19 or 20 or 21 or 22 or 23 or 24	2,752,924
26	4 and 25	2,349
27	26	2,349
28	limit 27 to yr="2015 -Current"	1,527

Table 3. Search strategy for CINAHL (searched 12 April 2022)

#	Query	Results
S27	S4 AND S25 from 2015-2022	635
S26	S4 AND S25	851
S25	S5 OR S6 OR S7 OR S8 OR S9 OR S10 OR S11 OR S12 OR S13 OR S14 OR S15 OR S16 OR S17 OR S18 OR S19 OR S20 OR S21 OR S22 OR S23 OR S24	407,258
S24	(MH (Obstetric Surgical Procedures/adverse effects OR Cesarean section/adverse effects OR Vaginal Birth after Cesarean/adverse effects OR laparotomy/adverse effects OR Hysterectomy/adverse effects OR Intestinal Obstruction/epidemiology OR Intestinal Obstruction/complications OR Tissue Adhesions/epidemiology OR Tissue Adhesions/complications OR Venous Thrombosis/epidemiology OR Venous Thromboembolism/epidemiology OR Pneumonia/epidemiology OR Chronic Pain/epidemiology OR menorrhagia/epidemiology OR Dysmenorrhea/epidemiology OR Delivery, Obstetric/adverse effects)) OR ("bowel obstruction" OR "surgical injur*" OR "deep vein thrombosis" OR DVT OR "venous thrombosis" OR pneumonia OR "chronic pain" OR menorrhagia OR dysmenorrhoea OR subsequent pregnanc*)	134,338
S23	(MH (Placenta Previa OR Placenta Diseases OR Abruptio Placentae OR Uterine Rupture)) OR ("placenta previa" OR "placenta accrete" OR "placenta accreta" OR "uterine rupture" OR "placental abruption" OR "abnormal placentation")	5,726
S22	MH (anemia) OR (Anemia OR anaemia)	33,607
S21	(MH (HIV Seropositivity OR Sepsis OR Puerperal Infection)) OR (HIV seroconversion OR HIV 9okophobia9t* OR sepsis OR septic OR septicaemia OR septicemia)	43,117
S20	MH(Gestational Weight Gain) OR ("weight retention" OR "weight gain" OR GWG)	21,093
S19	MH (mastitis) OR mastitis	941
S18	(MH (Postpartum Thyroiditis OR Thyroid Diseases OR Hypopituitarism)) OR ("postpartum thyroiditis" OR "post-partum thyroiditis" OR "grave's disease" OR "graves disease" OR "sheehan's syndrome" OR "sheehans syndrome" OR "postpartum thyroid" OR "post-partum thyroid")	4,706
S17	MH (Peripheral Nervous System Diseases) OR (Neuropath* OR "neural injur*" OR "nerve injur*" OR "lower extremity neuropath*" OR "lower extremity nerve injury" OR "femoral neuropath*" OR "peroneal neuropath*" OR "postpartum neuropath*" OR "post-partum neuropath*")	40,120
S16	MH (Cardiomyopathies) OR (PPCM OR "peripartum 9okophobia9thy*" OR "peripartum 9okophobia9thy*" OR "postpartum 9okophobia9thy*" OR "post-partum 9okophobia9thy*")	556
S15	MH ("Fear of childbirth") OR (Tokophobia or 9okophobia or "fear of childbirth")	399
S14	MH (Psychotic disorders) OR ("postpartum psychosis" OR "post-partum psychosis" OR psychotic disorder* OR "postpartum psychoses" OR "post-partum psychoses")	15,696
S13	(MH (Post traumatic stress disorder OR stress disorders, post traumatic)) OR (PTSD OR "post-traumatic stress disorder*" OR "post traumatic stress disorder*" OR "posttraumatic stress disorder")	21,904
S12	MH (Anxiety Disorders) OR (postpartum anxiety OR post-partum anxiety OR postnatal anxiety)	11,620
S11	MH (Postpartum Depression) OR ("postpartum depression" OR "post-partum depression" OR "postnatal depression" OR "post-natal depression" OR "Postpartum depressive disorder*" OR "post-partum depressive disorder*" OR "postpartum unipolar depression" OR "post-partum unipolar depression" OR "postnatal depressive disorder*" OR "post-natal depressive disorder*" OR "postnatal unipolar depression" OR "post-natal unipolar depression" OR PPD)	7,951
S10	(MH (Dyspareunia OR sexual dysfunction)) OR ("sexual dysfunction" OR dyspareunia OR "sexual problem*" OR "sexual function" OR "sexual health" OR "sexual pain")	24,089

S9	MH (Infertility) OR (“secondary infertility” OR subfertility)	12,212
S8	MH (Wound Infection) OR (“wound complication” OR “wound morbidity” OR “wound infection” OR “wound breakdown” OR “wound dehiscence”)	18,010
S7	“Cervical insufficiency” OR “cervical incompetence” OR “incompetent cervix” OR “short cervix” OR “shortened cervix” OR “cervical weakness”	845
S6	(MH (Pelvic Floor Disorders or Pelvic Organ Prolapse OR Urinary Incontinence) OR (“pelvic floor disorder*” OR “pelvic organ prolapse” OR PFD OR POP OR cystocele OR enterocele OR “rectal prolapse” OR “uterine prolapse” OR “visceral prolapse” OR “vaginal prolapse” OR “anterior compartment prolapse” OR “posterior compartment prolapse” OR “apical compartment prolapse” OR “uterine procidentia” OR “vaginal wall prolapse” OR “genital prolapse” OR “urinary incontinen*” OR “faecal incontinen*” OR “fecal incontinen*” OR “anal incontinen*” OR “flatus incontinen*”)	22,779
S5	(MH (Urinary Fistula OR Vaginal Fistula) OR) OR (“urinary fistula” OR “vaginal fistula” OR “rectal fistula” OR “vesicovaginal fistula” OR “vesico-vaginal fistula” OR “rectovaginal fistula” OR “recto-vaginal fistula” OR “perianal fistula” OR “peri-anal fistula” OR “obstetric fistula” OR “urethrovaginal fistula” OR UVF OR “ureterovaginal fistula” OR “urogenital fistula” OR fistula OR “anovaginal fistula” OR “ano-vaginal fistula” OR VVF OR RVF)	16,472
S4	S1 AND S2 AND S3	3,312
S3	(MH (Prevalence OR incidence)) OR (Prevalence OR Incidence OR Burden)	496,868
S2	(TI (Scoping review OR meta-analysis OR metaanalysis OR meta analysis OR systematic review OR rapid review OR living review)) OR (PT (Systematic Review OR Meta-Analysis)) OR JT (Cochrane Database Syst Rev)	181,510
S1	JT (MH (Pregnancy OR pregnant women OR postpartum period OR maternal health OR parturition OR childbirth) OR) OR (pregnant OR pregnancy OR postnatal OR postpartum OR maternal OR birth OR delivery OR parturition OR “labor” OR “labour” OR puerperium OR obstetric OR deliver* OR caesarean OR mother*)	583,588

Table 4. Search strategy for Epistemonikos (searched 12 April 2022)

#	Query
1	systematic OR meta-analysis OR meta analysis OR metaanalysis OR rapid review OR living review OR scoping review OR Cochrane Review
2	pregnancy OR pregnant OR postpartum OR post-partum OR postnatal OR maternal OR parturition OR childbirth OR birth OR delivery OR labour OR labor OR caesarean OR mother
3	prevalence OR incidence OR burden
4	#1 AND #2 AND #3, Filter Publication Type: Systematic Review
5	"urinary fistula" OR "vaginal fistula" OR "rectal fistula" OR "vesicovaginal fistula" OR "vesico-vaginal fistula" OR "rectovaginal fistula" OR "recto-vaginal fistula" OR "perianal fistula" OR "peri-anal fistula" OR "obstetric fistula" OR "urethrovaginal fistula" OR UVF OR "ureterovaginal fistula" OR "urogenital fistula" OR fistula OR "anovaginal fistula" OR "ano-vaginal fistula" OR VVF OR RVF) OR ("pelvic floor disorder*" OR "pelvic organ prolapse" OR PFD OR POP OR cystocele OR enterocele OR "rectal prolapse" OR "uterine prolapse" OR "visceral prolapse" OR "vaginal prolapse" OR "anterior compartment prolapse" OR "posterior compartment prolapse" OR "apical compartment prolapse" OR "uterine procidentia" OR "vaginal wall prolapse" OR "genital prolapse" OR "urinary incontinen*" OR "faecal incontinen*" OR "fecal incontinen*" OR "anal incontinen*" OR "flatus incontinen*" OR "Cervical insufficiency" OR "cervical incompetence" OR "incompetent cervix" OR "short cervix" OR "shortened cervix" OR "cervical weakness") OR ("wound complication" OR "wound morbidity" OR "wound infection" OR "wound breakdown" OR "wound dehiscence") OR ("secondary infertility" OR subfertility) OR ("sexual dysfunction" OR dyspareunia OR "sexual problem*" OR "sexual function" OR "sexual health" OR "sexual pain") OR ("postpartum depression" OR "post-partum depression" OR "postnatal depression" OR "post-natal depression" OR "Postpartum depressive disorder*" OR "post-partum depressive disorder*" OR "postpartum unipolar depression" OR "post-partum unipolar depression" OR "postnatal depressive disorder*" OR "post-natal depressive disorder*" OR "postnatal unipolar depression" OR "post-natal unipolar depression" OR PPD) OR (postpartum anxiety OR post-partum anxiety OR postnatal anxiety OR PTSD OR "post-traumatic stress disorder*" OR "post traumatic stress disorder*" OR "posttraumatic stress disorder") OR ("postpartum psychosis" OR "post-partum psychosis" OR psychotic disorder* OR "postpartum psychoses" OR "post-partum psychoses") OR (tokophobia OR tocophobia OR "fear of childbirth") OR (PPCM OR "peripartum cardiomyopath*" OR "peripartum cardiomyopath*" OR "postpartum cardiomyopath*" OR "post-partum cardiomyopath*") OR (Neuropath* OR "neural injur*" OR "nerve injur*" OR "lower extremity neuropath*" OR "lower extremity nerve injury" OR "femoral neuropath*" OR "peroneal neuropath*" OR "postpartum neuropath*" OR "post-partum neuropath*") OR ("postpartum thyroiditis" OR "post-partum thyroiditis" OR "grave's disease" OR "graves disease" OR "sheehan's syndrome" OR "sheehans syndrome" OR "postpartum thyroid" OR "post-partum thyroid") OR (mastitis) OR ("weight retention" OR "weight gain" OR GWG) OR (HIV seroconversion OR HIV seropositiv* OR sepsis OR septic OR septicaemia OR septicemia) OR (anemia OR anaemia) OR ("uterine rupture" OR Abruptio Placentae OR "placental abruption" OR "abnormal placentation") OR (postoperative complications OR laparotomy OR hysterectomy OR uterine rupture repair OR surgical site infection OR pelvic adhesion* OR bowel obstruction OR surgical injury OR surgical injuries OR scar complication OR deep vein thrombosis OR DVT OR pneumonia OR menorrhagia OR dysmenorrhoea OR "chronic pain" OR subsequent pregnancy OR subsequent pregnancies
6	#4 AND #5

Figure 1. PRISMA diagram of systematic reviews presenting epidemiological data on conditions of interest

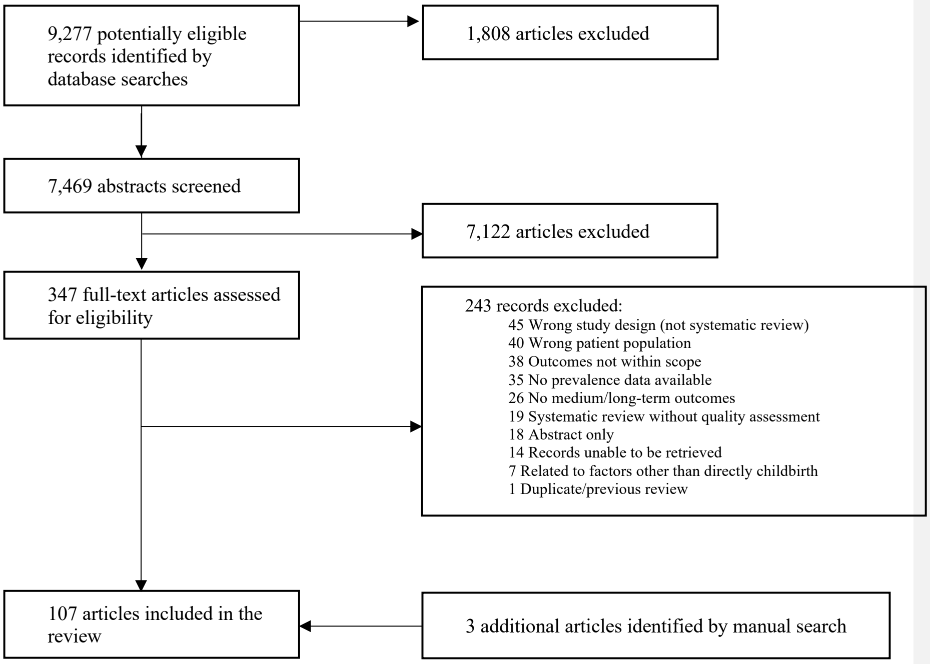

Table 4. Modified[5] AMSTAR assessments of identified systematic reviews

Author (year)	Overall Score	AMSTAR Checklist Questions															
		1	2	3	4	5	6	7	8	9	10	11	12	13	14	15	16
Banaei (2021)[2]	Critically Low	Yes	PY	Yes	Yes	Yes	Yes	Yes	Yes	Yes	No	Yes	Yes	Yes	Yes	Yes	Yes
Baradaran (2021)[3]	Low	Yes	PY	No	PY	No	No	Yes	PY	Yes	No	N/A	N/A	Yes	No	No	Yes
Bij de Vaate (2014)[4]	Moderate	Yes	PY	Yes	Yes	Yes	Yes	Yes	Yes	No	No	N/A	N/A	No	Yes	N/A	No
Blondon (2016)[5]	Low	Yes	PY	Yes	No	Yes	Yes	Yes	Yes	Yes	No	Yes	No	No	Yes	Yes	Yes
Chen (2020)[6]	Critically Low	Yes	Yes	No	PY	Yes	Yes	No	Yes	Yes	No	Yes	Yes	Yes	Yes	Yes	Yes
Chen (2022)[7]	High	Yes	PY	Yes	PY	Yes	Yes	Yes	No	Yes	No	Yes	Yes	Yes	Yes	Yes	Yes
Christopher (2019)[8]	Critically Low	Yes	Yes	Yes	Yes	Yes	No	Yes	PY	Yes	No	N/A	N/A	No	No	No	Yes
Cowgill (2015)[9]	Moderate	Yes	PY	No	Yes	No	Yes	Yes	Yes	Yes	No	N/A	N/A	Yes	No	N/A	Yes
De Mucio (2019)[10]	Critically Low	Yes	Yes	Yes	Yes	Yes	Yes	No	Yes	Yes	No	Yes	No	No	Yes	No	Yes
Dekel (2017)[11]	Low	Yes	No	No	PY	No	No	No	No	Yes	No	No	Yes	No	Yes	No	No
Dennis (2017)[12]	Low	Yes	PY	Yes	Yes	Yes	Yes	Yes	Yes	Yes	No	Yes	Yes	Yes	Yes	No	No
Downes (2017)[13]	Critically Low	Yes	No	Yes	No	No	No	No	Yes	No	No	N/A	N/A	No	Yes	N/A	Yes
Drake (2014)[14]	Critically Low	Yes	No	Yes	Yes	Yes	Yes	Yes	Yes	Yes	No	Yes	Yes	No	Yes	No	Yes
Fawcett (2019)[15]	Critically Low	Yes	PY	Yes	Yes	No	No	Yes	No	PY	Yes	Yes	Yes	Yes	Yes	No	Yes
Frigerio (2019)[16]	Critically Low	Yes	PY	No	Yes	Yes	Yes	Yes	Yes	No	No	N/A	N/A	No	No	N/A	Yes
Gray (2019)[17]	Low	Yes	Yes	Yes	Yes	Yes	Yes	No	Yes	Yes	No	No	No	No	Yes	No	Yes
Hahn-Holbrook (2017)[18]	Critically Low	Yes	No	Yes	Yes	No	No	Yes	No	No	No	Yes	No	No	Yes	Yes	Yes
Hartmann (2005)[19]	Low	No	No	Yes	Yes	Yes	Yes	No	PY	PY	No	Yes	No	Yes	Yes	No	Yes
Isogai (2019)[20]	Critically Low	No	No	No	No	No	No	No	No	No	No	N/A	N/A	No	No	N/A	Yes
Kalra (2022)[21]	Critically Low	Yes	Yes	No	Yes	Yes	Yes	Yes	Yes	Yes	No	N/A	N/A	Yes	No	N/A	Yes
Keag (2018)[22]	Low	Yes	Yes	Yes	Yes	Yes	Yes	No	Yes	Yes	No	Yes	No	No	Yes	No	No
Korzeniewski (2019)[23]	Critically Low	Yes	No	Yes	PY	No	No	Yes	Yes	Yes	No	N/A	N/A	Yes	No	N/A	Yes
Liu (2022)[24]	Low	Yes	Yes	No	Yes	Yes	Yes	Yes	Yes	Yes	No	Yes	No	No	No	Yes	Yes
Manresa (2019)[25]	Critically Low	Yes	Yes	Yes	Yes	Yes	Yes	Yes	Yes	Yes	No	Yes	No	No	Yes	No	Yes

[5] Selected AMSTAR Items were modified to improve suitability for systematic reviews of prevalence/incidence. The modifications included the following: Item 3 was met if authors provided an explanation of the selection of non-randomised study for interventions; the criteria 'included/consulted content experts in the field' was removed from Item 4; and for Item 7, providing the number and description of reasons for exclusion was deemed sufficient to meet the criteria 'provide a list of all potentially relevant studies that were read in full-text form but excluded from the review'.

Marshall (2011)[26]	High	No	No	No	PY	Yes	No	Yes	Yes	Yes	No	Yes	Yes	Yes	No	No	Yes
Meng (2015)[27]	Critically Low	No	No	No	PY	Yes	Yes	No	Yes	Yes	No	Yes	Yes	No	Yes	Yes	Yes
Moossdorff (2021)[28]	Critically Low	Yes	Yes	Yes	Yes	Yes	Yes	Yes	Yes	Yes	No	Yes	Yes	Yes	No	Yes	Yes
Nicholson (2006)[29]	Moderate	Yes	No	Yes	No	Yes	Yes	No	Yes	PY	No	Yes	No	No	No	No	No
Nielsen-Scott (2022)[30]	Critically Low	Yes	Yes	Yes	Yes	Yes	Yes	Yes	Yes	Yes	No	Yes	Yes	Yes	Yes	Yes	Yes
Nilsson (2018)[31]	Low	Yes	No	Yes	PY	Yes	Yes	Yes	Yes	Yes	No	N/A	N/A	Yes	No	N/A	Yes
O'Connell (2017)[32]	Critically Low	No	Yes	Yes	PY	Yes	Yes	No	Yes	No	No	Yes	Yes	No	Yes	No	Yes
Rider Sleutel (2020)[33]	Critically Low	Yes	No	No	PY	Yes	No	Yes	Yes	No	No	N/A	N/A	No	No	N/A	No
Shorey (2018)[34]	Critically Low	Yes	Yes	Yes	Yes	Yes	Yes	No	Yes	Yes	No	Yes	No	No	Yes	Yes	Yes
Sideris (2020)[35]	Critically Low	Yes	Yes	No	Yes	Yes	Yes	Yes	No	Yes	No	Yes	Yes	Yes	Yes	Yes	Yes
Smeets (2021)[36]	Critically Low	Yes	No	Yes	Yes	Yes	Yes	Yes	Yes	Yes	No	Yes	Yes	Yes	No	No	Yes
Tahtinen (2016)[37]	Moderate	Yes	Yes	Yes	Yes	Yes	Yes	No	Yes	Yes	No	Yes	Yes	No	Yes	Yes	Yes
Tulandi (2016)[38]	Critically Low	Yes	No	No	PY	Yes	Yes	Yes	Yes	Yes	No	N/A	N/A	No	No	N/A	No
VanderKruik (2017)[39]	Critically Low	Yes	Yes	Yes	Yes	Yes	Yes	Yes	Yes	Yes	No	N/A	N/A	Yes	Yes	N/A	Yes
Wang (2020)[40]	Critically Low	Yes	Yes	No	PY	Yes	Yes	No	Yes	Yes	Yes	Yes	Yes	Yes	No	Yes	Yes
Wang (2021)[41]	Moderate	Yes	No	No	Yes	Yes	Yes	Yes	No	No	No	N/A	N/A	No	No	N/A	No
Wang (2021)[42]	Low	Yes	Yes	Yes	Yes	Yes	Yes	Yes	Yes	Yes	No	Yes	Yes	No	Yes	No	Yes
Weibel (2016)[43]	Low	Yes	No	Yes	No	Yes	Yes	Yes	No	No	No	Yes	No	No	Yes	No	Yes
Wilson (2020)[44]	Critically low	Yes	No	Yes	Yes	Yes	Yes	Yes	Yes	No	No	Yes	No	No	Yes	No	Yes
Woody (2017)[45]	Moderate	Yes	No	Yes	Yes	No	No	No	Yes	No	No	Yes	No	No	Yes	No	Yes
Yildiz (2017)[46]	Critically Low	Yes	No	Yes	Yes	No	No	Yes	Yes	PY	No	Yes	Yes	Yes	Yes	No	No
Yimer (2019)[47]	Low	Yes	No	Yes	PY	No	Yes	Yes	Yes	Yes	No	N/A	N/A	No	No	N/A	Yes

N/A: No meta-analysis conducted; **PY**: Partial Yes

A detailed list of AMSTAR Checklist questions can be found from: Shea BJ, Reeves BC, Wells G, Thuku M, Hamel C, Moran J, Moher D, Tugwell P, Welch V, Kristjansson E, Henry DA. AMSTAR 2: a critical appraisal tool for systematic reviews that include randomised or non-randomised studies of healthcare interventions, or both. BMJ. 2017 Sep 21;358:j4008

References

1. Shea BJ, Reeves BC, Wells G, et al. AMSTAR 2: a critical appraisal tool for systematic reviews that include randomised or non-randomised studies of healthcare interventions, or both. *BMJ* 2017; **358**: j4008.
2. Banaei M, Kariman N, Ozgoli G, et al. Prevalence of postpartum dyspareunia: A systematic review and meta-analysis. *Int J Gynaecol Obstet* 2021; **153**(1): 14-24.
3. Baradaran K. Risk of Uterine Rupture with Vaginal Birth after Cesarean in Twin Gestations. *Obstet Gynecol Int* 2021; **2021**: 6693142.
4. Bij de Vaate AJ, van der Voet LF, Naji O, et al. Prevalence, potential risk factors for development and symptoms related to the presence of uterine niches following Cesarean section: systematic review. *Ultrasound in obstetrics & gynecology : the official journal of the International Society of Ultrasound in Obstetrics and Gynecology* 2014; **43(4)**: 372-82.
5. Blondon M, Casini A, Hoppe KK, Boehlen F, Righini M, Smith NL. Risks of Venous Thromboembolism After Cesarean Sections: A Meta-Analysis. *CHEST* 2016; **150**(3): 572-96.
6. Chen Y, Yang X, Guo C, et al. Prevalence of Post-Traumatic Stress Disorder Following Caesarean Section: A Systematic Review and Meta-Analysis. *Journal of Women's Health (15409996)* 2020; **29**(2): 200-9.
7. Chen Y, Geng X, Zhou H, et al. Systematic review and meta-analysis of evaluation of selective cesarean section in postpartum pelvic floor function recovery under perineal ultrasound. *Ann Palliat Med* 2022; **11**(2): 730-42.
8. Christopher S, McCullough J, Snodgrass SJ, Cook C. Predictive Risk Factors for First-Onset Lumbopelvic Pain in Postpartum Women: A Systematic Review. *Journal of Women's Health Physical Therapy* 2019; **43**(3): 127-35.
9. Cowgill KD, Bishop J, Norgaard AK, Rubens CE, Gravett MG. Obstetric fistula in low-resource countries: An under-valued and under-studied problem - systematic review of its incidence, prevalence, and association with stillbirth. *BMC Pregnancy and Childbirth* 2015; **15(1) (no pagination)**.
10. De Mucio B, Serruya S, Alemán A, Castellano G, Sosa CG. A systematic review and meta-analysis of cesarean delivery and other uterine surgery as risk factors for placenta accreta. *International Journal of Gynecology & Obstetrics* 2019; **147**(3): 281-91.
11. Dekel S, Stuebe C, Dishy G. Childbirth Induced Posttraumatic Stress Syndrome: A Systematic Review of Prevalence and Risk Factors. *Frontiers in psychology* 2017; **8**: 560.
12. Dennis CL, Falah-Hassani K, Shiri R. Prevalence of antenatal and postnatal anxiety: Systematic review and meta-analysis. *British Journal of Psychiatry* 2017; **210(5)**: 315-23.
13. Downes KL, Grantz KL, Shenassa ED. Maternal, Labor, Delivery, and Perinatal Outcomes Associated with Placental Abruption: A Systematic Review. *Am J Perinatol* 2017; **34**(10): 935-57.
14. Drake AL, Wagner A, Richardson B, John-Stewart G. Incident HIV during pregnancy and postpartum and risk of mother-to-child HIV transmission: a systematic review and meta-analysis. *PLoS medicine* 2014; **11**(2): e1001608.
15. Fawcett EJ, Fairbrother N, Cox ML, White IR, Fawcett JM. The Prevalence of Anxiety Disorders During Pregnancy and the Postpartum Period: A Multivariate Bayesian Meta-Analysis. *J Clin Psychiatry* 2019; **80**(4).
16. Frigerio M, Mastrolia SA, Spelzini F, Manodoro S, Yohay D, Weintraub AY. Long-term effects of episiotomy on urinary incontinence and pelvic organ prolapse: a systematic review. *Arch Gynecol Obstet* 2019; **299**(2): 317-25.
17. Gray TG, Vickers H, Jha S, Jones GL, Brown SR, Radley SC. A systematic review of non-invasive modalities used to identify women with anal incontinence symptoms after childbirth. *Int Urogynecol J* 2019; **30**(6): 869-79.
18. Hahn-Holbrook J, Cornwell-Hinrichs T, Anaya I. Economic and Health Predictors of National Postpartum Depression Prevalence: A Systematic Review, Meta-analysis, and Meta-Regression of 291 Studies from 56 Countries. *Frontiers in psychiatry* 2017; **8**: 248.
19. Hartmann K, Viswanathan M, Palmieri R, Gartlehner G, Thorp J, Jr., Lohr KN. Outcomes of routine episiotomy: a systematic review. *Jama* 2005; **293**(17): 2141-8.

20. Isogai T, Kamiya CA. Worldwide Incidence of Peripartum Cardiomyopathy and Overall Maternal Mortality. *Int Heart J* 2019; **60**(3): 503-11.
21. Kalra H, Tran T, Romero L, Chandra P, Fisher J. Burden of severe maternal peripartum mental disorders in low- and middle-income countries: a systematic review. *Archives of Women's Mental Health* 2022; **25(2)**: 267-75.
22. Keag OE, Norman JE, Stock SJ. Long-term risks and benefits associated with cesarean delivery for mother, baby, and subsequent pregnancies: Systematic review and meta-analysis. *PLoS Med* 2018; **15**(1): e1002494.
23. Korzeniewski R, Kiemle G, Slade P. Mothers' experiences of sex and sexual intimacy in the first postnatal year: a systematic review. *Sexual & Relationship Therapy* 2021; **36**(2/3): 219-37.
24. Liu X, Wang S, Wang G. Prevalence and Risk Factors of Postpartum Depression in Women: A Systematic Review and Meta-analysis. *Journal of clinical nursing* 2021; **08**.
25. Manresa M, Pereda A, Bataller E, Terre-Rull C, Ismail KM, Webb SS. Incidence of perineal pain and dyspareunia following spontaneous vaginal birth: a systematic review and meta-analysis. *International urogynecology journal* 2019; **30**(6): 853-68.
26. Marshall NE, Fu R, Guise JM. Impact of multiple cesarean deliveries on maternal morbidity: a systematic review. *American Journal of Obstetrics & Gynecology* 2011; **205**(3): 262.e1-8.
27. Meng K, Hu X, Peng X, Zhang Z. Incidence of venous thromboembolism during pregnancy and the puerperium: a systematic review and meta-analysis. *Journal of Maternal-Fetal & Neonatal Medicine* 2015; **28**(3): 245-53.
28. Moossdorff-Steinhauser HFA, Berghmans BCM, Spaanderman MEA, Bols EMJ. Prevalence, incidence and bothersomeness of urinary incontinence between 6 weeks and 1 year post-partum: a systematic review and meta-analysis. *Int Urogynecol J* 2021; **32**(7): 1675-93.
29. Nicholson WK, Robinson KA, Smallridge RC, Ladenson PW, Powe NR. Prevalence of postpartum thyroid dysfunction: a quantitative review. *Thyroid : official journal of the American Thyroid Association* 2006; **16**(6): 573-82.
30. Nielsen-Scott M, Fellmeth G, Opondo C, Alderdice F. Prevalence of perinatal anxiety in low- and middle-income countries: A systematic review and meta-analysis. *Journal of affective disorders* 2022; **306**: 71-9.
31. Nilsson C, Hessman E, Sjoblom H, et al. Definitions, measurements and prevalence of fear of childbirth: A systematic review. *BMC Pregnancy and Childbirth* 2018; **18(1) (no pagination)**.
32. O'Connell MA, Leahy-Warren P, Khashan AS, Kenny LC, O'Neill SM. Worldwide prevalence of tocophobia in pregnant women: systematic review and meta-analysis. *Acta Obstetricia et Gynecologica Scandinavica* 2017; **96**(8): 907-20.
33. Rider Sleutel M, True B, Webb J, Valdez E, Van Thi Tran M. Integrative Review of Lower Extremity Nerve Injury During Vaginal Birth. *JOGNN: Journal of Obstetric, Gynecologic & Neonatal Nursing* 2020; **49**(6): 507-24.
34. Shorey S, Chee CYI, Ng ED, Chan YH, Tam WWS, Chong YS. Prevalence and incidence of postpartum depression among healthy mothers: A systematic review and meta-analysis. *Journal of Psychiatric Research* 2018; **104**: 235-48.
35. Sideris M, McCaughey T, Hanrahan JG, et al. Risk of obstetric anal sphincter injuries (OASIS) and anal incontinence: A meta-analysis. *European Journal of Obstetrics and Gynecology and Reproductive Biology* 2020; **252**: 303-12.
36. Smeets CFA, Vergeldt TFM, Notten KJB, Martens FMJ, van Kuijk SMJ. Association between levator ani avulsion and urinary incontinence in women: A systematic review and meta-analysis. *Int J Gynaecol Obstet* 2021; **153**(1): 25-32.
37. Tähtinen RM, Cartwright R, Tsui JF, et al. Long-term Impact of Mode of Delivery on Stress Urinary Incontinence and Urgency Urinary Incontinence: A Systematic Review and Meta-analysis. *Eur Urol* 2016; **70**(1): 148-58.
38. Tulandi T, Cohen A. Emerging Manifestations of Cesarean Scar Defect in Reproductive-aged Women. *Journal of Minimally Invasive Gynecology* 2016; **23**(6): 893-902.
39. VanderKruik R, Barreix M, Chou D, et al. The global prevalence of postpartum psychosis: a systematic review. *BMC psychiatry* 2017; **17**(1): 272.
40. Wang K, Xu X, Jia G, Jiang H. Risk Factors for Postpartum Stress Urinary Incontinence: a Systematic Review and Meta-analysis. *Reprod Sci* 2020; **27**(12): 2129-45.

41. Wang Z, Liu J, Shuai H, et al. Mapping global prevalence of depression among postpartum women. *Translational Psychiatry* 2021; **11(1) (no pagination)**.
42. Wang Y, Liu H, Chen Y, Yu X, Zhang Y, Kong X. Prevalence and risk factors of chronic pain after cesarean section: A systematic review. [Chinese]. *Chinese Journal of Evidence-Based Medicine* 2021; **21(10)**: 1195-202.
43. Weibel S, Neubert K, Jelting Y, et al. Incidence and severity of chronic pain after caesarean section: A systematic review with meta-analysis. *European Journal of Anaesthesiology* 2016; **33(11)**: 853-65.
44. Wilson E, Woodd SL, Benova L. Incidence of and Risk Factors for Lactational Mastitis: A Systematic Review. *Journal of Human Lactation* 2020; **36**(4): 673-86.
45. Woody CA, Ferrari AJ, Siskind DJ, Whiteford HA, Harris MG. A systematic review and meta-regression of the prevalence and incidence of perinatal depression. *J Affect Disord* 2017; **219**: 86-92.
46. Yildiz PD, Ayers S, Phillips L. The prevalence of posttraumatic stress disorder in pregnancy and after birth: A systematic review and meta-analysis. *Journal of Affective Disorders* 2017; **208**: 634-45.
47. Yimer H, Woldie H. Incidence and Associated Factors of Chronic Pain After Caesarean Section: A Systematic Review. *Journal of Obstetrics and Gynaecology Canada* 2019; **41(6)**: 840-54.

Supplementary File S2. Methods related to systematic review of guidelines

We aimed to identify and summarise high-quality guidelines and recommendations pertaining to the prevention and management of medium- and long-term conditions arising from labour and childbirth. We adopted the Institute of Medicine's definition of a guideline as "statements that include recommendations, intended to optimize patient care, that are informed by a systematic review of evidence and an assessment of the benefits and harms of alternative care options".[5] Guidelines provide recommendations for evidence-based decision-making in clinical care; and hence recommendations within guidelines should be based on systematic assessments of available evidence.

Eligibility criteria

Guidelines meeting the following criteria were included:

- Meets the definition of clinical practice guidelines by the Institute of Medicine (i.e. informed by a systematic review of evidence);
- Developed by a nationally recognized committee or a medical society for national, regional or international use. Guidelines that were developed for subnational, hospital or local use are not eligible.
- Published since 1 Jan 2010
- Accessible in the public domain
- Provided recommendations related to prevention, screening, diagnosis or management of the medium/long term conditions of interest. Guidelines on nomenclature, terminology and definitions, or guidelines related to professional education and training, were not included.

If multiple versions of the same guideline were identified, only the most recent version was included.

Identifying eligible guidelines

We aimed to identify all eligible guidelines related to the specified medium and long-term conditions. To achieve this, a hierarchical approach was used:

1. We used scoping searches to develop a list of reputable organisations producing guidelines that are used internationally for the conditions of interest. We searched websites of these organisations for any guideline published from 2010 onwards that pertained to a condition of interest. See Table 1 below for a list of these organizations.

2. We also conducted a systematic search of MEDLINE, Trip database (using Guideline filter function) and Guideline International Network website for guidelines for the conditions of interest (see Table 2 below for the search strategy). Recovered citations were screened in duplicate using Covidence. First, we screened titles and abstracts of these citations. For potentially eligible citations, we recovered and screened the full texts. The aforementioned eligibility criteria were applied.

3. Findings from #1 and #2 were compiled and duplicates removed, and individual guidelines were mapped for each condition of interest. Where multiple versions of the same guideline

were identified, we kept only the most recent guideline.

4. For all guidelines, we extracted information on their characteristics and performed an AGREE-II assessment.[48] AGREE-II consists of 23 key items organized within 6 domains: scope and purpose, stakeholder involvement, rigour of development, clarity of presentation, applicability, and editorial independence. We calculated a total score as recommended by AGREE-II developers. We considered guidelines with an AGREE-II score of 6 or more as "high quality" which were included for extraction of recommendations. For any guidelines with an overall AGREE-II score of 5.5 (averaged score between reviewers), one reviewer specialising in guideline methodology re-assessed the guideline for eligibility.

Identifying eligible recommendations

5. From these high-quality guidelines, we extracted individual recommendations, and tagged them by recommendation type. We were specifically interested in recommendations pertaining directly to the identification, prevention and management of the conditions of interest – other recommendations in the same guideline that did not meet this definition were not extracted (recommendations regarding definitions, classification were excluded).

   The following types (with operational definitions) were used:

   - **Clinical Assessment** – Recommendations relating to the initial or ongoing assessment of women, usually involves history, physical examination or special tests. Recommendations might include referral on the basis of assessment findings.
   - **Screening** – Recommendations relating to formal screening of women for a condition of interest, typically involving a pre-specified screening tool. May include referral on the basis of screening findings.
   - **Diagnostic procedures** – Recommendations relating to the diagnosis of conditions of interest. Typically includes the use of special tests, and can include making management decisions based on their findings.
   - **Education** – Recommendations relating to education and training of healthcare providers for the conditions of interest.
   - **Prevention** – Recommendations relating to behavioural or lifestyle interventions, pharmacotherapies, psychosocial interventions (such as weight loss, diet, nutrition, physical activity), surgical procedures, complementary therapies or other interventions for preventing the condition of interest.
   - **Management** – Recommendations relating to behavioural or lifestyle interventions, pharmacotherapies, psychosocial interventions (such as weight loss, diet, nutrition, physical activity), surgical procedures, complementary therapies, post-intervention follow-up or referral to higher level of care for management the condition of interest.
   - **Health systems** – Recommendations relating to health policies (such as place of birth or level of care required for certain conditions), use of clinical guidelines or protocols, models of care (including multidisciplinary involvement), ensuring availability of certain health services (such as blood transfusion) when providing care, workforce or staffing arrangements, or use of clinical registries.
   - **Person-centred care** – Recommendations relating to interpersonal aspects of care, including respectful and non-stigmatizing care, discussing birth experiences, the need to

be aware of the emotional or psychological impact of conditions, and helping women set their goals of care.

- **Counselling** – Recommendations related to supporting women through information, counselling, shared decision-making and informed consent processes. This can include helping women consider benefits and risks of treatment options, discussing their options, or using patient decision aids.

6. We only extracted those recommendations that specifically pertained to the medium and long-term conditions of interest.
7. We summarised the identified recommendations for or against (“Recommended” or “not recommendation”) and identified where different guidelines agreed or disagreed.

A PRISMA flowchart summarizing the findings of above steps is provided below.

Table 1. List of identified guideline development organizations which were hand-searched

Academy of Breastfeeding Medicine
Academy of Nutrition and Dietetics
American Academy of Family Physicians
American Academy of Neurology
American Association of Clinical Endocrinology
American Psychiatric Association
American Psychological Association
American Society for Reproductive Medicine
American Society of Colon and Rectal Surgeon
American Society of Colon and Rectal Surgeons
American Society of Hematology
American Thyroid Association
American Urogynecologic Society
Association of Women's Health, Obstetric and Neonatal Nurses
Australasian Society of Infectious Diseases
British Committee for Standards in Hematology
British HIV Association
British Society of Haematology
British Thyroid Association
Canadian Cardiovascular Society
French National College of Obstetricians and Gynecologists
Endocrine Society
European AIDS Clinical Society
European Association of Urology
European Psychiatric Association
European Society of Cardiology
European Society of Endocrinology
European Thyroid Association
European Venous Forum, North American Thrombosis Forum, International Union of Angiology, and International Union of Phlebology
French academic societies
French College of Obstetricians & Gynaecologists
HIV Medicine Association
Institute of Obstetricians and Gynaecologists, Royal College of Physicians of Ireland
International Antiviral Society
International Continence Society
International Society for Fertility Preservation
International Society for the Study of Women's Sexual Health
International Society for Traumatic Stress Studies
International Society of Nephrology
International Society on Thrombosis and Haemostasis
International Urogynecological Association
Kidney Disease Improving Global Outcomes
Royal Australian and New Zealand College of Obstetrics and Gynaecology
Scottish Intercollegiate Guidelines Network
Society for Maternal-Fetal Medicine
Society of Urodynamics, Female Pelvic Medicine & Urogenital reconstruction International Scientific Committee
Thrombosis Canada
UK Continence Society

Urogynaecological Society of Australia and New Zealand
Urological Society of Australia and New Zealand
US Preventive Services Task Force
US Preventive Services Task Force
World Federation of Societies of Biological Psychiatry
World Society of Emergency Surgery and Peritoneum and Survey Society

Table 2. Search strategy for PubMed (searched 10 May 2022)

#	Query	Results
1	Pregnancy[MeSH] OR Pregnant Women[MeSH] OR Postpartum Period[MeSH] OR Maternal Health[MeSH] OR Delivery, Obstetric[MeSH] OR Peripartum Period[MeSH] OR pregnant[tiab] OR pregnancy[tiab] OR postnatal[tiab] OR postpartum[tiab] OR maternal[tiab] OR Parturition[MeSH] OR childbirth[tiab] OR birth[tiab] OR delivery[tiab] OR parturition[tiab] OR labor[tiab] OR labour[tiab] OR puerperium[tiab] OR obstetric[tiab] OR delivery[tiab] OR caesarean[tiab] OR mother*[tiab] OR women[tiab] OR Obstetrician[tiab] OR Gynecologist[tiab] OR Obstetrics[tiab] OR Gynecology[tiab] OR Gynaecology[tiab] OR female[tiab] OR Postnatal Care/methods*[MeSH]	3,375,244
2	Practice Guidelines as Topic[MeSH] OR Clinical Decision-Making[MeSH] OR Consensus[MeSH] OR Societies, Medical[MeSH] OR Clinical Protocols[MeSH] OR guideline*[ti] OR consensus*[tiab] OR protocol*[tiab] OR “best practice”[tiab] OR “position statement”[tiab] OR “policy statement*”[tiab] OR standards*[ti] OR “practice parameter”[tiab] OR “best practice”[tiab] OR CPG[tiab] OR CPGs[tiab] OR recommendat*[ti] OR Guideline[pt] OR Practice Guideline[pt]	1,214,289
3	Urinary Fistula/diagnosis*[MeSH] OR Urinary Fistula/therapy*[MeSH] OR Vaginal Fistula/diagnosis*[MeSH] OR Vaginal Fistula/therapy*[MeSH] OR “urinary fistula”[tiab] OR “vaginal fistula”[tiab] OR “recto?vaginal fistula”[tiab] OR “vesico?vaginal fistula”[tiab] OR “peri?anal fistula”[tiab] OR “obstetric fistula”[tiab] OR “urethrovaginal fistula”[tiab] OR “UVF”[tiab] OR “uterovaginal fistula”[tiab] OR “urogenital fistula”[tiab] OR “ano?vaginal fistula”[tiab]	8,038
4	Pelvic Floor Disorders/diagnosis*[MeSH] OR Pelvic Floor Disorders/therapy*[MeSH] OR Pelvic Organ Prolapse/diagnosis*[MeSH] OR Pelvic Organ Prolapse/therapy*[MeSH] OR Pelvic Organ Prolapse/surgery*[MeSH] OR Pelvic Pain/diagnosis*[MeSH] OR Pelvic Pain/therapy*[MeSH] OR Rectum Prolapse/diagnosis*[MeSH] OR Rectum Prolapse/therapy*[MeSH] OR Urinary Incontinence/diagnosis*[MeSH] OR Urinary Incontinence/therapy*[MeSH] OR Fecal Incontinence/diagnosis*[MeSH] OR Fecal Incontinence/therapy*[MeSH] OR "pelvic floor disorder"[tiab] OR "pelvic organ prolapse"[tiab] OR cystocele[tiab] OR enterocele[tiab] OR "rectal prolapse"[tiab] OR "uterine prolapse"[tiab] OR "vaginal prolapse"[tiab] OR "urinary incontinence"[tiab] OR "faecal incontinence"[tiab] OR “fecal incontinence”[tiab] OR "anal incontinence"[tiab] OR "flatus incontinence"[tiab]	64,384
5	"Cervical insufficiency"[tiab] OR "cervical incompetence"[tiab] OR "incompetent cervix"[tiab] OR "short cervix"[tiab] OR "shortened cervix"[tiab] OR "cervical weakness"[tiab]	1,918
6	Surgical Wound Infection/diagnosis*[MeSH] OR Surgical Wound Infection/therapy*[MeSH] OR "surgical wound complication"[tiab] OR "surgical wound infection"[tiab] OR "surgical wound breakdown"[tiab] OR "wound dehiscence"[tiab]	29,241
7	Infertility, Female/diagnosis*[MeSH] OR Infertility, Female/therapy*[MeSH] OR “secondary infertility”[tiab] OR sub?fertility[tiab]	17,689
8	Dyspareunia/diagnosis*[MeSH] OR Dyspareunia/therapy*[MeSH] OR dyspareunia[tiab] OR “sexual function*”[tiab] OR “sexual health”[tiab] OR “sexual pain”[tiab]	31,572
9	Depression, Postpartum/diagnosis[MeSH] OR Depression, Postpartum/therapy OR “postpartum depression”[tiab] OR “post-partum depression”[tiab] OR “postnatal depression”[tiab] OR “postnatal depression”[tiab] OR Anxiety	66,461

	Disorders/diagnosis[MeSH] OR “postpartum anxiety”[tiab] OR “post-partum anxiety” OR “postnatal anxiety”[tiab] OR “post-natal anxiety” OR Psychotic disorders/diagnosis*[MeSH] OR Psychotic Disorders/therapy*[MeSH] OR “postpartum psychosis”[tiab] OR “post-partum psychosis”[tiab] OR “psychotic disorder*”[tiab] OR tokophobia[tiab] OR tocophobia[tiab]	
10	Cardiomyopathies/diagnosis*[MeSH] OR Cardiomyopathies/therapy*[MeSH] OR PPCM[tiab] OR “peripartum cardiomyopath*”[tiab] OR “postpartum cardiomyopath*”[tiab] OR “post-partum cardiomyopath*”	56,768
11	Peripheral Nerve Injuries/diagnosis*[MeSH] OR Peripheral Nerve Injuries/therapy[MeSH] OR neuropathy OR neuropathies OR “neural injury” OR “neural injuries” OR “nerve injury” OR “nerve injuries” OR “nerve damage”	142,545
12	Postpartum Thyroiditis/diagnosis*[MeSH] OR Postpartum Thyroiditis/therapy*[MeSH] OR Hyperthyroidism/diagnosis*[MeSH] OR Hyperthyroidism/therapy*[MeSH] OR Hypopituitarism/diagnosis* OR Hypopituitarism/therapy*[MeSH] OR “graves disease”[tiab] OR “sheehan’s syndrome”[tiab] OR “postpartum thyroiditis”[tiab] OR “post-partum thyroiditis”[tiab]	32,904
13	Mastitis/diagnosis*[MeSH] OR Mastitis/therapy*[MeSH] OR mastitis[tiab]	14,828
14	Gestational Weight Gain/diagnosis*[MeSH] OR Gestational Weight Gain/therapy*[MeSH] OR “weight retention”[tiab] OR “gestational weight gain”[tiab] OR “GWG”[tiab]	6,849
15	HIV Seropositivity/diagnosis*[MeSH] OR HIV Seropositivity/therapy*[MeSH] OR Pregnancy Complications, Infectious/therapy*[MeSH] OR Pregnancy Complications, Infectious/diagnosis*[MeSH] OR Puerperal Infection/diagnosis*[MeSH] OR Puerperal Infection/therapy[MeSH] OR “HIV seroconversion”[tiab] OR “HIV seroconversion”[tiab] OR “HIV Seropositivity”[tiab] OR “postpartum sepsis” OR “puerperal sepsis”	30,658
16	Anemia, Iron-Deficiency/diagnosis*[MeSH] OR Anemia, Iron-Deficiency/therapy*[MeSH] OR Puerperal Disorders/blood[MeSH] OR “postpartum anemia” OR “postpartum anaemia” OR “post-partum anemia” OR “post-partum anaemia”	7,785
17	Placenta Previa/diagnosis*[MeSH] OR Placenta Previa/therapy*[MeSH] OR Placenta Diseases/diagnosis*[MeSH] OR Placenta Diseases/therapy*[MeSH] OR “placenta previa”[tiab] OR “placenta accrete”[tiab] OR “placenta accreta”[tiab] OR Uterine Rupture/diagnosis*[MeSH] OR Uterine Rupture/therapy*[MeSH] OR “uterine rupture”[tiab] OR Abruptio Placentae/diagnosis*[MeSH] OR Abruptio Placentae/therapy*[MeSH] OR “placental abruption”[tiab] OR “abnormal placentation”[tiab]	16,358

18	Gynecologic Surgical Procedures[MeSH] OR Postoperative Complications[MeSH] OR Obstetric Surgical Procedures[MeSH] OR Obstetric Surgical Procedures/adverse effects[MeSH] OR Cesarean Section/adverse effects[MeSH] OR Vaginal birth After Cesarean[MeSH] OR Vaginal Birth After Cesarean/adverse effects[MeSH] OR "Cesarean section*"[tiab] OR Pregnancy Complications/therapy*[MeSH] OR Pregnancy Complications/diagnosis*[MeSH] OR Pregnancy Complications/therapy*[MeSH] OR Pregnancy Complications/prevention&control*[MesH] OR Puerperal Disorders/diagnosis*[MeSH] OR Puerperal Disorders/therapy*[MeSH] OR Cystocele/complications[MeSH] OR Venous Thromboembolism/prevention&control*[MeSH] OR Venous Thrombosis/prevention&control*[MeSH] OR Postoperative Complications/prevention&control*[MeSH] OR Delivery, Obstetric/adverse effects[MeSH] OR Laparotomy/adverse effects[MeSH] OR Intestinal Obstruction/diagnosis*[MeSH] OR Intestinal Obstruction/therapy*[MeSH] OR Tissue Adhesions/diagnosis*[MeSH] OR Tissue Adhesions/therapy*[MeSH] OR "bowel obstruction"[tiab] OR "surgical injur*"[tiab] OR "deep vein thrombosis"[tiab] OR DVT OR "venous thrombosis"[tiab] OR pneumonia/diagnosis*[MeSH] OR pneumonia/therapy*[MeSH] OR pneumonia[tiab] OR Chronic Pain/diagnosis*[MeSH] OR Chronic Pain/therapy*[MeSH] "chronic pain"[tiab] OR Dysmenorrhea/diagnosis*[MeSH] OR Dysmenorrhea/therapy*[MeSH] OR menorrhagia[tiab] OR dysmenorrhoea[tiab] OR "subsequent pregnanc*"[tiab] OR Puerperal Disorders/diagnosis*[MeSH] OR Puerperal Disorders/therapy*[MeSH]	39,466
19	#3 AND #4 AND #5 AND #6 AND #7 AND #8 AND #9 AND #10 AND #11 AND #12 AND #13 AND #14 AND #15 AND #16 AND #17 AND #18	544,121
20	#1 AND #2 AND #19	10,257
21	#20 Filters: from 2010 – 2022	6,901

Table 2. Search strategy for TRIP database (searched 10 May 2022)

#	Query
1	Pregnancy OR Pregnant OR Postpartum OR Post-partum OR Maternal OR Postnatal OR Post-natal OR Parturition OR Delivery OR Obstetric OR Peripartum OR Peri-partum OR Childbirth OR Birth OR Delivery OR Labor OR Labour OR Puerperium OR Caesarean OR Mother
2	Fistula OR Pelvic Floor Disorders OR Incontinence OR Prolapse OR Cystocele OR enterocele OR Cervical insufficiency OR cervical incompetence OR shortened cervix OR Wound infection OR Wound complication OR wound breakdown OR Wound dehiscence OR subfertility OR Secondary infertility OR Dyspareunia OR sexual dysfunction OR Postpartum depression OR Post-partum depression OR Post-natal depression OR Postpartum depressive disorder OR Anxiety OR Post traumatic stress disorder OR PTSD OR Psychosis OR psychoses OR tokophobia OR tokophobia OR Cardiomyopathy OR Cardiomyopathies OR PPCM OR Neuropathy OR Neural Injury OR Thyroiditis OR Hypopituitarism OR Graves disease OR Sheehan's Syndrome OR Mastitis OR Weight retention OR GWG OR Seropositivity OR Seropositive OR Seroconversion OR sepsis OR septic OR septicaemia OR anemia OR anaemia OR Placenta previa OR Placenta accrete OR Uterine rupture OR abnormal placentation OR Postoperative complication OR Intestinal obstruction OR Tissue Adhesion OR Bowel Obstruction OR Venous Thrombosis OR Venous Thromboembolism OR deep vein thrombosis OR DVT OR Chronic Pain OR menorrhagia OR Dysmenorrhea
3	#1 AND #2
4	#3 Filters: from 2010 – 2022

Figure 1. PRISMA diagram for identification, screening, and selection of included guidelines

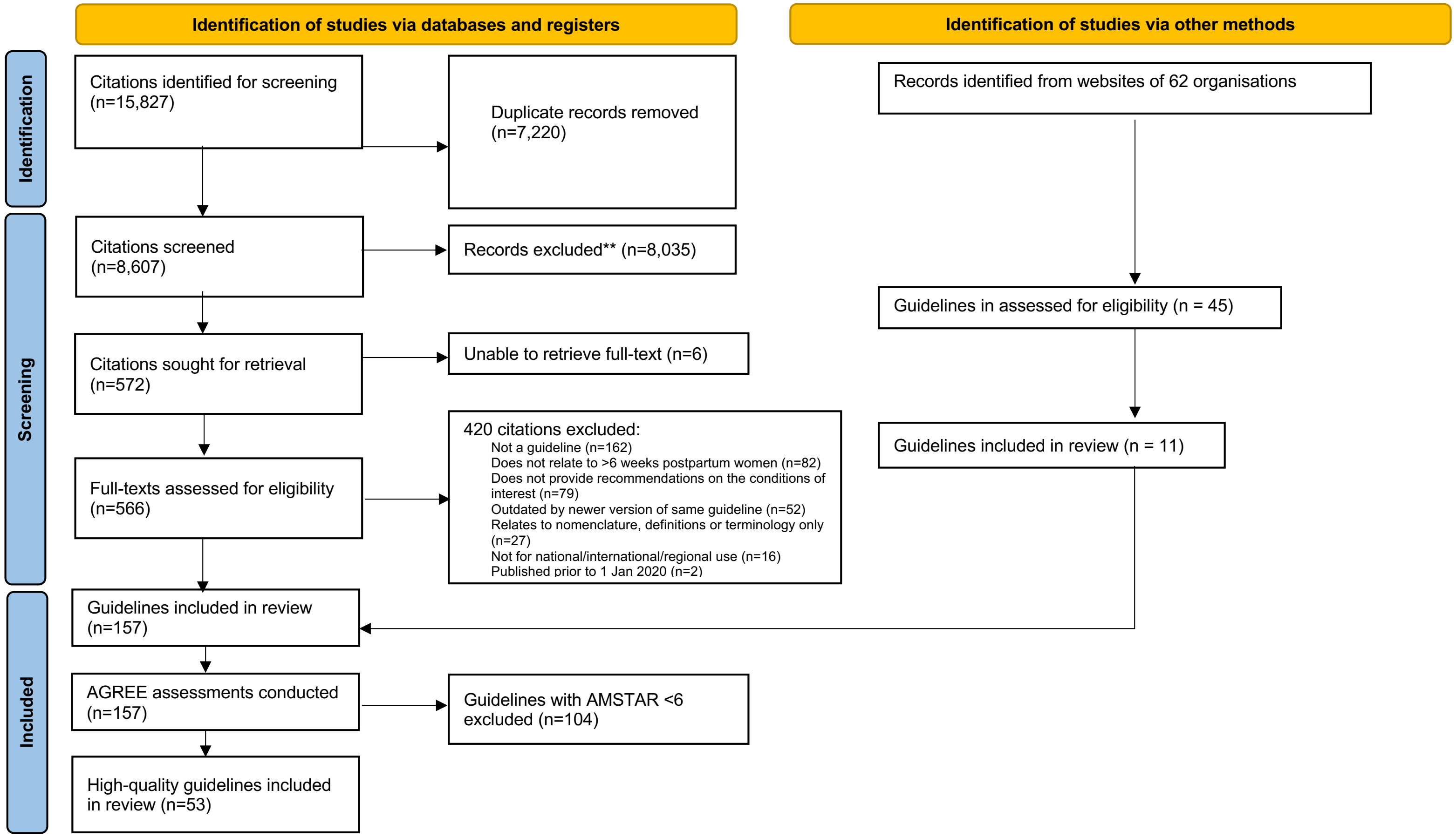

*Consider, if feasible to do so, reporting the number of records identified from each database or register searched (rather than the total number across all databases/registers).

**If automation tools were used, indicate how many records were excluded by a human and how many were excluded by automation tools.

From: Page MJ, McKenzie JE, Bossuyt PM, Boutron I, Hoffmann TC, Mulrow CD, et al. The PRISMA 2020 statement: an updated guideline for reporting systematic reviews. BMJ 2021;372:n71. doi: 10.1136/bmj.n71. For more information, visit: http://www.prisma-statement.org/

References

1. Brouwers MC, Kho ME, Browman GP, et al. AGREE II: advancing guideline development, reporting, and evaluation in health care. *Prev Med* 2010; **51**(5): 421-4.

Supplementary Table S1. Epidemiological data on medium- and long-term conditions of interest[6]

Condition	Evidence from representative household surveys or large maternity registers	Systematic reviews by AMSTAR rating (quality of reviews)		
		High or moderate	Low	Critically low
**Conditions related to birth**				
Genitourinary conditions				
**Fistula (any type)**	A meta-analysis by Maheu-Giroux et al (2015) of 19 national household surveys (DHS and MICS) including 262,100 respondents reported the lifetime prevalence of symptoms of vaginal fistula at 3.0 cases per 1,000 women of reproductive age (95% CI 1.36-5.5).[49]<br>Data from three DHS surveys estimated lifetime prevalence of fistula symptoms among women who ever had a live birth ranged from 3.4% in Uganda (6,410 women) to 5.8% in Malawi (9,298 women).[50]	*None identified*	Cowgill et al (2015) reported the incidence estimates of obstetric fistula in LMICs ranged from 0 to 4.09 cases per 1,000 births.[51] The prevalence estimates of obstetric fistula ranged from 0 to 81 cases per 1,000 women.	Adler et al (2013) reported an overall pooled prevalence of obstetric fistula in LMICs was 0.29 per 1,000 women (10 studies including 34,505 women; 95% CI 0.00-1.07).[52] The pooled incidence was 0.09 per 1,000 recently pregnant women (3 studies including 21,413 women; 95% CI 0.01-0.25).
**Pelvic organ prolapse**	Analysis of three NHANES surveys conducted among U.S. women between 2005 and 2010 reported the weighted prevalence of pelvic organ prolapse increased with parity, ranging from 1.4% to 4.5% (6,112 women).[53]	Chen et al (2022) reported that pelvic organ prolapse after vaginal birth occurred in 13.7% of women (4 studies, 826/2,289 women; OR=0.29, 95% CI 0.09-0.89).[7]	*None identified*	Keag et al (2018) reported pelvic organ prolapse after vaginal birth was 6% (2 studies, 2,055/34,310 women).[22]
**Anal incontinence**	Analysis of three NHANES surveys conducted among U.S. women between 2005 and 2010 reported the weighted prevalence of faecal incontinence increased with parity, ranging from 8.3% to 11.8% (6,112 women).[53]	Sideris et al (2020) reported that among women who gave birth vaginally (33 studies including 4,174 women) the overall pooled prevalence of anal sphincter defect on ultrasound was 26% (95% CI 21-30) and anal incontinence symptoms was 19% (25 studies including 2,523 women; 95% CI 14-25).[54]	*None identified*	Gray et al (2019) reported on faecal incontinence using validated questionnaires[17]:<br>- 27% (SD=0.3%, 16 studies) at 6 weeks to 1 year postpartum<br>- 33% (SD=0.2%, 6 studies) between 2 and 5 years postpartum<br>- 26% (SD=0.3%, 12 studies) at >5 years postpartum
**Urinary incontinence**	Analysis of three NHANES surveys conducted among U.S. women between 2005 and 2010 reported the weighted prevalence of moderate-to-severe urinary incontinence increased with parity, ranging from 11.8% to 23.9% (6,112 women).[53]	Moossdorff-Steinhauser et al (2021) reported the mean (weighted) prevalence of urinary incontinence in postpartum women between 6 weeks and 12 months in HICs was 31% (24 studies, 24,064 women; 95% CI 26-36).[28] Among these 24 studies the prevalence ranged between 10-63%.<br><br>Tahtinen et al (2016) reported the absolute prevalence of stress urinary incontinence among	Wang et al (2020) reported the incidence of postpartum SUI was 13.3% (46 studies of 73,010 participants). The incidence ranged from 0.4% in Iran to 26.6% in Sweden.[40]	Smeets et al (2021) identified seven studies which reported the prevalence of stress urinary incontinence in women with levator ani muscle avulsion ranging from 29-69% in women at least one year after childbirth.[36]<br><br>Keag et al (2018) reported urinary incontinence after vaginal birth was 15% (8 studies, 7,713/51,194 women).[22]

[6] For several conditions, no systematic reviews of prevalence were identified: Cervical incompetence, wound complications, Grave's disease, Sheehan's syndrome, postpartum weight retention, abnormal placentation, chronic anaemia, some post-procedural complications (surgical site infection, pelvic adhesions, bowel obstruction, surgical injury, pneumonia, menorrhagia, dysmenorrhea, sexual dysfunction), some post-episiotomy complications (poorly-healing perineum or perineal pain after episiotomy) and conditions related to operative vaginal birth

Condition	Evidence from representative household surveys or large maternity registers	Systematic reviews by AMSTAR rating (quality of reviews)		
		High or moderate	Low	Critically low
		women with vaginal birth ranged from 12% to 19%.[37]<br>Chen et al (2022) reported that stress urinary incontinence occurred postpartum in 7.9% of women (4 studies, 826/2289 women).[7]		
**Sexual health conditions**				
**Secondary infertility**	Mascarenhas et al (2012) analysed 277 DHS/RHS reported the prevalence of secondary infertility was 10.5% (95% CI 9.5 to 11.7%).[55]	*None identified*	*None identified*	*None identified*
**Postpartum sexual dysfunction (including dyspareunia)**	*None identified*	Banaei et al (2020) reported the overall prevalence of dyspareunia was 35% (95% CI 29-41%)[2]:<br>- 43% from 2 to 6 months postpartum (12 studies; 95% CI 36-50%),<br>- 22% from 6 to 12 months postpartum (9 studies; 95% CI 15-29%)<br>- 40% from 12 to 24 months postpartum (1 study).[2]<br>Pooled prevalence on the resumption of sexual intercourse:<br>- 89% from 2-6 months postpartum (11 studies; 95% CI 83-93%)<br>- 99% from 6-12 months postpartum (11 studies; 95% CI 97-100%).[2]	Korzeniewski et al (2021) review reported 82-90% of women resumed sexual intercourse between 6-9 months postpartum (3 studies), of which at least one problem was reported in 36-43% of women at 6 months, and 80-91% at 12 months (5 studies).[56]	Manresa et al (2019) reported the pooled prevalence of dyspareunia for women with an intact perineum following spontaneous vaginal birth was 15% at 6 months postpartum (3 studies including 423 women; 95% CI 0.05-0.44%) and 16% at 12 months postpartum (2 studies including 350 women; 95% CI 0.08-0.32%).[57]
**Mental health conditions**				
**Postpartum depression**	*None identified*	*None identified*	Liu et al (2021) reported the pooled prevalence of postpartum depression was 14% overall (95% CI 12-15%)[58]:<br>- 12.9% at 8 weeks postpartum (3 studies, 2,350 women; 95% CI 9.5-12.6%)<br>- 17.4% at 12 weeks postpartum (2 studies, 1,999 women; 95% CI 5.9-28.9%)<br>- 13.6% at 24 weeks postpartum (7 studies, 86,103 women; 95% CI 10.4-16.8%)<br><br>Prevalence varied by country economic level:<br>- 17.0% in developing countries (16 studies including 15,196 women; 95% CI 14-20%)	Four systematic reviews were identified:<br>- Woody et al (2017) identified 79 studies and reported the pooled prevalence of postnatal depression was 18.7% in LMICs (95% CI 17.8-19.7%) and 9.5% in HICs (95% CI 8.9-10.1%).[45]<br>- Hahn-Holbrook et al (2017) reported the pooled prevalence of postpartum depression was 17.7% between 4-52 weeks postpartum (291 studies including 296,284 women; 95% CI 16.6-18.8%) and ranged from 3.1% in Singapore to 37.7% in Chile.[59]<br>- Shorey et al (2018) reported the pooled prevalence of postpartum depression was 16% at 4-6 months (18 studies; 95% CI 13-19%), 20% at 7-12 months (7 studies; 95% CI 11-29%), and 25% at >12 months postpartum (6

Condition	Evidence from representative household surveys or large maternity registers	Systematic reviews by AMSTAR rating (quality of reviews)		
		High or moderate	Low	Critically low
			- 11.0% in developed countries (13 studies including 113,819 women; 95% CI 8.8-13.2%).	studies; 95% CI 12-39%) in women without a previous history of depression.[60]<br>- Wang et al (2021) reported the pooled prevalence of postpartum depression was 15.9% at 3-6 months (89 studies including 277,293 women; 95% CI 14-18%), 17.9% at 6-12 months (67 studies including 1,560,464 women; 95% CI 13-24%), and 17.9% at >12 months (15 studies including 11,374 women; 95% CI 14-23%) postpartum.[61]
**Post-traumatic stress disorder (PTSD)**	*None identified*	*None identified*	Fawcett et al (2019) reported the pooled prevalence of PTSD was 1.1% (9 studies including 7,544 women; 95% CI 0.5-2.0).[15]	Yildiz et al (2017) reported a pooled prevalence of PTSD was 1.4% at 3 months (4 studies including 1,525 women; 95% CI 0.3-6.5%) and 6.8% at 6 months (5 studies including 1,136 women; 95% CI 1.6-24.5%) postpartum.[62]<br><br>Dekel et al (2017) reported the rate of postpartum PTSD assessed from 3 to <6 months postpartum was between 3.4% and 6.7%.[63]
**Postpartum psychosis**	*None identified*	VanderKruik et al (2017) identified one study that reported prevalence of postpartum psychosis (5 in 1,000 in United States).[64]<br><br>Kalra et al (2022) reported a cumulative incidence of postpartum psychosis of 1.1 per 1,000 births from the five studies identified. The prevalence between studies ranged from 1.1 to 16.7 per 1,000 births.[65]	*None identified*	*None identified*
**Anxiety disorders**	*None identified*	Nielsen-Scott et al (2022) reported the pooled prevalence of postpartum anxiety disorder was 16% (1 study including 113 women; 95% CI 13.5-18.9%) and self-reported anxiety symptoms was 24.4% (15 studies including 6,370 women; 95% CI 16.2-33.7%).[66]	Dennis et al (2017) reported the pooled prevalence of postpartum anxiety disorder was:<br>- 9.6% at 5–12 weeks postpartum (5 studies including 2,712 women; 95% CI 3.4–15.9%)<br>- 9.9% at 1-24 weeks postpartum (9 studies including 28,495 women; 95% CI 6.1-13.8%)<br>- 9.3% at >24 weeks postpartum (5 studies including 28,244 women; 95% CI 5.5-13.1%).[67]<br><br>Fawcett et al (2019) reported the pooled prevalence of postpartum generalized	*None identified*

Condition	Evidence from representative household surveys or large maternity registers	Systematic reviews by AMSTAR rating (quality of reviews)		
		High or moderate	Low	Critically low
			anxiety disorder was 2.6% (9 studies, 4,430 women; 95% CI 1.3-3.8%).[15]	
**Secondary tokophobia**	*None identified*	*None identified*	Nilsson et al (2018) reported prevalence of fear of childbirth (as defined by W-DEQ≥85) varied from 6.3-14.8% (10 studies conducted in 7 European countries).[68]	O'Connell et al (2017) reported the pooled prevalence of tokophobia was 12% (17 studies; 95% CI 10-14%) amongst multiparous women.[69]
**Cardiovascular conditions**				
**Peripartum cardiomyopathy**	Ijaz et al (2022) analysed data from the National Inpatient Sample database between 2004 and 2018 from USA and reported the prevalence of peripartum cardiomyopathy[70]:<br>- 33.5 per 100,000 live births in women aged 15-35 years<br>- 77.6 per 100,000 live births in women aged 36-54 years.	*None identified*	*None identified*	Isogai et al (2019) reviewed 31 studies from 15 countries, and reported the incidence of peripartum cardiomyopathy varied with highest rates in Nigeria (1 in 102 births) and lowest in Japan (1 in 15,533 births).[20]
**Venous thromboembolism (VTE)**	*None identified*	*None identified*	Meng et al (2015) [71] reported the pooled incidence rate of VTE during pregnancy and puerperium was 1.4 per 1000 (1.0–1.8 per 1000). However, a separate pooled analysis suggests the proportion of these VTE that occurs postpartum (rather than antepartum) was 57.5% (95% CI: 20.9-63.9).	*None identified*
**Neurological conditions**				
**Neuropathies/neural injury**	*None identified*	*None identified*	*None identified*	Rider Sleutel et al (2020) identified five studies, and reported that the incidence of lower extremity nerve injury up to 6 months postpartum ranged from 0.3-2.3%.[72]
**Chronic pain**	*None identified*	*None identified*	*None identified*	Christopher et al (2019) identified four studies - first-onset low back pain incidence ranged between 19% and 53%, and the mean incidence was 31.6%.[73]<br><br>Manresa et al (2019) reported an incidence of perineal pain of 11% for women with an intact perineum following spontaneous vaginal birth at 3 months postpartum (2 studies, 758 women; 95% CI 0.01 to 100%).[57]
**Endocrine conditions**				
**Postpartum thyroiditis**	*None identified*	*None identified*	*None identified*	Nicholson et al (2006) reported the pooled prevalence of postpartum thyroid dysfunction was 8.1% (15 studies including 7,846 women; 95% CI 7.8-8.2%) and ranged between 0.9 to 11.7%.<br>- 7.9% at up to 6 months postpartum (7 studies including 3,568 women; 95% CI 7.7-8.1%)

Condition	Evidence from representative household surveys or large maternity registers	Systematic reviews by AMSTAR rating (quality of reviews)		
		High or moderate	Low	Critically low
				6.4% at up to 9 months postpartum (2 studies including 584 women; 95% CI 6.3-6.5%)<br>9.2% at up to 12 months postpartum (4 studies including 3,186 women; 95% CI 9.1-9.3%).[74]
**Breast conditions**				
**Mastitis**	*None identified*	*None identified*	*None identified*	Wilson et al (2020) reported the pooled incidence rate of lactational mastitis at 0-25 weeks postpartum was 11.1 episodes per 1,000 breastfeeding weeks (3 studies including 2,535 women; 95% CI 10.2-12.0). Prevalence ranged from 2.5% to 20%.[44]
**Infections**				
**HIV seroconversion**	*None identified*	*None identified*	*None identified*	Drake et al (2014) reported the pooled HIV incidence rate was 2.9/100 person-years postpartum (7 studies including 14,065 person-years; 95% CI 1.8-4.0).[14]
**Impacts on subsequent pregnancy**				
**Placenta previa or accreta**	*None identified*	*None identified*	*None identified*	De Mucio et al (2019) identified 16 studies and reported the increased absolute risk of placenta accreta following CS. The absolute risk was:<br>3.3 per 10,000 for no CS<br>12.9 per 10,000 after one CS<br>41.3 per 10,000 after two CS<br>78.3 per 10,000 after three CS<br>217 per 10,000 after four CS<br>230 per 10,000 after five or more CS.[75]
**Placental abruption**	*None identified*	*None identified*	*None identified*	Downes et al (2017) reported the incidence of abruption ranged from 0.01% to 5.1%.[13]
**Uterine rupture**	Vandenberghe et al (2018) analysed data from INOSS (nine European countries) and reported the prevalence of complete uterine rupture was 3.3 per 10,000 births (95% CI 3.1-3.5). Prevalence varied widely between countries, from 1.6 to 7.8 per 10,000 births.[76]	*None identified*	*None identified*	*None identified*
**Conditions specific to C-section, laparotomy, or hysterectomy**				
**Uterine and wound complications**	*None identified*	*None identified*	*None identified*	Weibel et al (2016) reported the pooled incidence of chronic wound pain after caesarean section[77]:<br>15.4% for 3-6 months postpartum (15 studies including 4,475 women; 95% CI 9.9-20.9%)<br>11.5% for 6-11 months postpartum (14 studies including 3,345 women; 95% CI 8.1-15.0%)

Condition	Evidence from representative household surveys or large maternity registers	Systematic reviews by AMSTAR rating (quality of reviews)		
		High or moderate	Low	Critically low
				11.2% at ≥12 months postpartum (12 studies including 3,451 women; 95% CI 7.4-15.0%)
**Uterine rupture**	Vandenberghe et al (2018) analysed data from INOSS (nine European countries)[76] and reported the overall prevalence of complete uterine rupture was:<br>- 22 per 10,000 births (95% CI 21-24) in women with previous CS<br>- 35 per 10,000 births (95% CI 32-37) in women with previous CS and labour.<br>- Prevalence varied widely between countries, ranging from 8 to 68 per 10,000 births in women with previous CS section, and from 16 to 80 per 10,000 births in women with previous Cs and labour.<br><br>Motomura et al (2017) analysed data from the WHO Multicountry Survey and reported the incidence of uterine rupture in women with prior CS was 45 per 10,000 births, ranging from 0.1% to 2.5%.[76]	*None identified*	Baradaran et al (2021) reported the absolute risk of uterine rupture in vaginal birth after caesarean (VBAC) and planned repeat CS was 0.87% and 0.09%, respectively (4 retrospective studies including 7,699 women). Uterine rupture rates ranged from 0-1.69% (4 studies including 7,699 women with twin pregnancies and a previous caesarean).[3]	*None identified*
**Scar complications**	*None identified*	*None identified*	*None identified*	Tulandi et al (2016) reported the prevalence of CS scar defect ranged between 24-88% on ultrasound examination (8 studies including 1,048 women).[78]<br><br>Bij de Vaate et al (2013) reported the prevalence of a niche (CS scar defect) in women with a history of CS varied between 56-84% using SHG (3 studies) and 24-80% using TVS (4 studies).[79]
**Deep vein thrombosis**	*None identified*	*None identified*	*None identified*	Blondon et al (2016) reported the pooled incidence of VTE following CS was 2.6 per 1,000 CS births (32 studies, 120,603 women; 95% CI 1.7-3.5). This was higher in studies with a longer and better postpartum follow-up (4.3 per 1,000 CS, 95% CI 1.0-4.3, 4 studies).[80]
**Pain/nerve injury/chronic pain**	*None identified*	*None identified*	*None identified*	Yimer et al (2019) reported the incidence of chronic post-CS pain at 2-6 months postpartum ranged between 4-41.8% (17 studies).[81]<br><br>Weibel et al (2016) reported the pooled incidence of chronic postsurgical pain 3-6 months after CS was

Condition	Evidence from representative household surveys or large maternity registers	Systematic reviews by AMSTAR rating (quality of reviews)		
		High or moderate	Low	Critically low
				15.4% (15 studies including 4,475 women; 95% CI 9.9-20.9).[77]<br><br>Wang et al (2021) reported the pooled prevalence of chronic pain post-CS was:<br>• 19% at 2 to 5 months (29 studies; 95% CI 12-23%)<br>• 13% at 6 to 11 months (22 studies; 95% CI 9-17%),<br>• 8% at 12 months or more (15 studies; 95% CI: 6-10%).[82]<br><br>Keag et al (2018) reported pelvic pain after CS occurred in 33/2,449 cases (1.3%) in 2 studies identified.[22]
**Secondary infertility**	*None identified*	*None identified*	*None identified*	Keag et al (2018) reported subfertility amongst women with previous CS was 43% (11 studies, 246,096/567,155 women).[22]
**Pelvic organ prolapse**	Analysis of three NHANES surveys conducted among U.S. women between 2005 and 2010 reported the weighted prevalence of pelvic organ prolapse among women who had CS was 1.9% (723 women; 95% CI 1.1-3.3) and among women with previous hysterectomy was 5.4% (1,717 women; 95% CI 4.0-7.3).[53]	Chen et al (2022) reported that pelvic organ prolapse after CS occurred in 21.2% of women (175/826 women).[7]	*None identified*	Keag et al (2018) reported pelvic organ prolapse after CS was 2.3% (2 studies, 116/4,989 women).[22]
**Urinary incontinence**	Analysis of three NHANES surveys conducted among U.S. women between 2005 and 2010 reported the weighted prevalence of urinary incontinence among women who had CS was 12.7% (723 women; 95% CI 10.4-15.4). The weighted prevalence among women with previous hysterectomy was 29.5% (1,717 women; 95% CI 26.8-32.3%).[53]	Tahtinen et al (2016) reported the absolute prevalence of stress urinary incontinence among women with CS ranged from 5% to 15%.[37]<br>Chen et al (2022) reported that stress urinary incontinence affected 10.2% of women after CS (84/826 women).[7]	*None identified*	Keag et al (2018) reported urinary incontinence after CS occurred in 14% of women (8 studies, 1,034/7306 women).[22]
**Anal incontinence**	Analysis of three NHANES surveys conducted among U.S. women between 2005 and 2010 reported the weighted prevalence of faecal incontinence among women who had CS was 6.4% (723 women; 95% CI 4.6-8.8%). The weighted prevalence among women with previous hysterectomy was 16.6% (1717 women; 95% CI 14.6-18.8).[53]	*None identified*	*None identified*	Keag et al reported faecal incontinence after CS was 4.3% (5 studies, 234/6449 women).[22]

<table>
<tr><th rowspan="2">Condition</th><th rowspan="2">Evidence from representative household surveys or large maternity registers</th><th colspan="3">Systematic reviews by AMSTAR rating (quality of reviews)</th></tr>
<tr><th>High or moderate</th><th>Low</th><th>Critically low</th></tr>
<tr><td>Post-traumatic stress disorder</td><td>None identified</td><td>None identified</td><td>Chen et al (2020) reported the pooled prevalence of PTSD after caesarean section was:
• 9.0% at >8 weeks postpartum (1 study, 122 women; 95% CI 4.6-15.6)
• 4.8% at 4 weeks to >12 months postpartum (1 study, 145 women; 95% CI 2.0-9.7%).[83]</td><td>None identified</td></tr>
<tr><td>Adverse effect on subsequent pregnancies</td><td>None identified</td><td>None identified</td><td>Marshall et al (2011) reported:
• absolute risk of previa associated with CS was 12 per 1,000 deliveries (95% CI 8-15 per 1,000)
• Overall incidence of abruption with any previous CS was 1.2-1.5%, and the rate of abruption was 10.3-15 per 1,000 deliveries.
• The reported incidence of placenta accreta ranged from 11% in women with 1 previous CS to 67% in women with ≥5 CS deliveries.[84]</td><td>De Mucio et al (2019) identified 16 studies and reported the increased absolute risk of placenta accreta following CS. The absolute risk was:
• 3.3 per 10,000 for no CS
• 12.9 per 10,000 after one CS
• 41.3 per 10,000 after two CS
• 78.3 per 10,000 after three CS
• 217 per 10,000 for four CS
• 230 per 10,000 for five or more CS.[75]</td></tr>
<tr><td colspan="5">Conditions related to episiotomy</td></tr>
<tr><td>Incontinence</td><td>None identified</td><td>None identified</td><td>None identified</td><td>Hartmann et al (2005) reviewed prospective cohort studies of women who received episiotomy and reported the prevalence of urinary incontinence ranged from 0.7-29.1% (4 studies, 667 women), anal incontinence ranged from 2.8-29.1% (3 studies, 675 women).[19]

Frigerio et al (2019) analysed the long-term impact (≥5 years) of episiotomy, which reported the prevalence of urinary incontinence ranging between 0% and 75.2% (18 studies).[16]</td></tr>
<tr><td>Postpartum sexual dysfunction (including dyspareunia)</td><td>None identified</td><td>None identified</td><td>None identified</td><td>Hartmann et al (2005) reviewed prospective cohort studies of women who received episiotomy and reported the prevalence of sexual dysfunction ranged from 7.9-64.9% (5 studies, 960 women).[19]

Manresa et al (2019) reported the incidence of dyspareunia for women with a repaired second-degree tear or episiotomy following spontaneous vaginal birth was:
• 16% at 6-7 weeks postpartum (2 studies; 95% CI 2-100%)</td></tr>
</table>

Condition	Evidence from representative household surveys or large maternity registers	Systematic reviews by AMSTAR rating (quality of reviews)		
		High or moderate	Low	Critically low
				- 19% at 3 months postpartum (3 studies; 95% CI 13-28%).

DHS, MICS: NHANES: INOSS. PERISTAT

Note: 95% CIs were reported for all prevalence and/or incidence estimates if available from the original review.

References

1. Maheu-Giroux M, Filippi V, Samadoulougou S, et al. Prevalence of symptoms of vaginal fistula in 19 sub-Saharan Africa countries: a meta-analysis of national household survey data. *Lancet Glob Health* 2015; **3**(5): e271-8.
2. Kiersten J, A. P. Incontinence data from the Demographic and Health Surveys: comparative analysis of a proxy measurement of vaginal fistula and recommendations for future population-based data collection. Calverton, Maryland, USA, 2008.
3. Cowgill KD, Bishop J, Norgaard AK, Rubens CE, Gravett MG. Obstetric fistula in low-resource countries: an under-valued and under-studied problem--systematic review of its incidence, prevalence, and association with stillbirth. *BMC Pregnancy Childbirth* 2015; **15**: 193.
4. Adler AJ, Ronsmans C, Calvert C, Filippi V. Estimating the prevalence of obstetric fistula: a systematic review and meta-analysis. *BMC Pregnancy Childbirth* 2013; **13**: 246.
5. Wu JM, Vaughan CP, Goode PS, et al. Prevalence and trends of symptomatic pelvic floor disorders in U.S. women. *Obstet Gynecol* 2014; **123**(1): 141-8.
6. Chen Y, Geng X, Zhou H, et al. Systematic review and meta-analysis of evaluation of selective cesarean section in postpartum pelvic floor function recovery under perineal ultrasound. *Ann Palliat Med* 2022; **11**(2): 730-42.
7. Keag OE, Norman JE, Stock SJ. Long-term risks and benefits associated with cesarean delivery for mother, baby, and subsequent pregnancies: Systematic review and meta-analysis. *PLoS Med* 2018; **15**(1): e1002494.
8. Sideris M, McCaughey T, Hanrahan JG, et al. Risk of obstetric anal sphincter injuries (OASIS) and anal incontinence: A meta-analysis. *Eur J Obstet Gynecol Reprod Biol* 2020; **252**: 303-12.
9. Gray TG, Vickers H, Jha S, Jones GL, Brown SR, Radley SC. A systematic review of non-invasive modalities used to identify women with anal incontinence symptoms after childbirth. *Int Urogynecol J* 2019; **30**(6): 869-79.
10. Moossdorff-Steinhauser HFA, Berghmans BCM, Spaanderman MEA, Bols EMJ. Prevalence, incidence and bothersomeness of urinary incontinence between 6 weeks and 1 year post-partum: a systematic review and meta-analysis. *Int Urogynecol J* 2021; **32**(7): 1675-93.
11. Tähtinen RM, Cartwright R, Tsui JF, et al. Long-term Impact of Mode of Delivery on Stress Urinary Incontinence and Urgency Urinary Incontinence: A Systematic Review and Meta-analysis. *Eur Urol* 2016; **70**(1): 148-58.
12. Wang K, Xu X, Jia G, Jiang H. Risk Factors for Postpartum Stress Urinary Incontinence: a Systematic Review and Meta-analysis. *Reprod Sci* 2020; **27**(12): 2129-45.
13. Smeets CFA, Vergeldt TFM, Notten KJB, Martens FMJ, van Kuijk SMJ. Association between levator ani avulsion and urinary incontinence in women: A systematic review and meta-analysis. *Int J Gynaecol Obstet* 2021; **153**(1): 25-32.
14. Mascarenhas MN, Flaxman SR, Boerma T, Vanderpoel S, Stevens GA. National, regional, and global trends in infertility prevalence since 1990: a systematic analysis of 277 health surveys. *PLoS Med* 2012; **9**(12): e1001356.
15. Banaei M, Kariman N, Ozgoli G, et al. Prevalence of postpartum dyspareunia: A systematic review and meta-analysis. *Int J Gynaecol Obstet* 2021; **153**(1): 14-24.
16. Korzeniewski R KG, Slade P. Mothers' experiences of sex and sexual intimacy in the first postnatal year: a systematic review. *Sexual and Relationship Therapy* 2019; **36**(2-3): 219-37.
17. Manresa M, Pereda A, Bataller E, Terre-Rull C, Ismail KM, Webb SS. Incidence of perineal pain and dyspareunia following spontaneous vaginal birth: a systematic review and meta-analysis. *Int Urogynecol J* 2019; **30**(6): 853-68.
18. Liu X, Wang S, Wang G. Prevalence and Risk Factors of Postpartum Depression in Women: A Systematic Review and Meta-analysis. *J Clin Nurs* 2022; **31**(19-20): 2665-77.
19. Woody CA, Ferrari AJ, Siskind DJ, Whiteford HA, Harris MG. A systematic review and meta-regression of the prevalence and incidence of perinatal depression. *J Affect Disord* 2017; **219**: 86-92.

20. Hahn-Holbrook J, Cornwell-Hinrichs T, Anaya I. Economic and Health Predictors of National Postpartum Depression Prevalence: A Systematic Review, Meta-analysis, and Meta-Regression of 291 Studies from 56 Countries. *Front Psychiatry* 2017; **8**: 248.
21. Shorey S, Chee CYI, Ng ED, Chan YH, Tam WWS, Chong YS. Prevalence and incidence of postpartum depression among healthy mothers: A systematic review and meta-analysis. *J Psychiatr Res* 2018; **104**: 235-48.
22. Wang Z, Liu J, Shuai H, et al. Mapping global prevalence of depression among postpartum women. *Transl Psychiatry* 2021; **11**(1): 543.
23. Fawcett EJ, Fairbrother N, Cox ML, White IR, Fawcett JM. The Prevalence of Anxiety Disorders During Pregnancy and the Postpartum Period: A Multivariate Bayesian Meta-Analysis. *J Clin Psychiatry* 2019; **80**(4).
24. Yildiz PD, Ayers S, Phillips L. The prevalence of posttraumatic stress disorder in pregnancy and after birth: A systematic review and meta-analysis. *J Affect Disord* 2017; **208**: 634-45.
25. Dekel S, Stuebe C, Dishy G. Childbirth Induced Posttraumatic Stress Syndrome: A Systematic Review of Prevalence and Risk Factors. *Front Psychol* 2017; **8**: 560.
26. VanderKruik R, Barreix M, Chou D, Allen T, Say L, Cohen LS. The global prevalence of postpartum psychosis: a systematic review. *BMC Psychiatry* 2017; **17**(1): 272.
27. Kalra H, Tran T, Romero L, Chandra P, Fisher J. Burden of severe maternal peripartum mental disorders in low- and middle-income countries: a systematic review. *Arch Womens Ment Health* 2022; **25**(2): 267-75.
28. Nielsen-Scott M, Fellmeth G, Opondo C, Alderdice F. Prevalence of perinatal anxiety in low- and middle-income countries: A systematic review and meta-analysis. *J Affect Disord* 2022; **306**: 71-9.
29. Dennis CL, Falah-Hassani K, Shiri R. Prevalence of antenatal and postnatal anxiety: systematic review and meta-analysis. *Br J Psychiatry* 2017; **210**(5): 315-23.
30. Nilsson C, Hessman E, Sjöblom H, et al. Definitions, measurements and prevalence of fear of childbirth: a systematic review. *BMC Pregnancy Childbirth* 2018; **18**(1): 28.
31. O'Connell MA, Leahy-Warren P, Khashan AS, Kenny LC, O'Neill SM. Worldwide prevalence of tocophobia in pregnant women: systematic review and meta-analysis. *Acta Obstet Gynecol Scand* 2017; **96**(8): 907-20.
32. Ijaz SH, Jamal S, Minhas AMK, et al. Trends in Characteristics and Outcomes of Peripartum Cardiomyopathy Hospitalizations in the United States Between 2004 and 2018. *Am J Cardiol* 2022; **168**: 142-50.
33. Isogai T, Kamiya CA. Worldwide Incidence of Peripartum Cardiomyopathy and Overall Maternal Mortality. *Int Heart J* 2019; **60**(3): 503-11.
34. Meng K, Hu X, Peng X, Zhang Z. Incidence of venous thromboembolism during pregnancy and the puerperium: a systematic review and meta-analysis. *J Matern Fetal Neonatal Med* 2015; **28**(3): 245-53.
35. Rider Sleutel M, True B, Webb J, Valdez E, Van Thi Tran M. Integrative Review of Lower Extremity Nerve Injury During Vaginal Birth. *J Obstet Gynecol Neonatal Nurs* 2020; **49**(6): 507-24.
36. Christopher S MJ, Snodgrass S.J., Cook C. Predictive Risk Factors for First-Onset Lumbopelvic Pain in Postpartum Women: A Systematic Review. *Journal of Women's Health Physical Therapy* 2049; **43**(3): 127-35.
37. Nicholson WK, Robinson KA, Smallridge RC, Ladenson PW, Powe NR. Prevalence of postpartum thyroid dysfunction: a quantitative review. *Thyroid* 2006; **16**(6): 573-82.
38. Wilson E, Woodd SL, Benova L. Incidence of and Risk Factors for Lactational Mastitis: A Systematic Review. *Journal of Human Lactation* 2020; **36**(4): 673-86.
39. Drake AL, Wagner A, Richardson B, John-Stewart G. Incident HIV during pregnancy and postpartum and risk of mother-to-child HIV transmission: a systematic review and meta-analysis. *PLoS medicine* 2014; **11**(2): e1001608.
40. De Mucio B, Serruya S, Alemán A, Castellano G, Sosa CG. A systematic review and meta-analysis of cesarean delivery and other uterine surgery as risk factors for placenta accreta. *Int J Gynaecol Obstet* 2019; **147**(3): 281-91.
41. Downes KL, Grantz KL, Shenassa ED. Maternal, Labor, Delivery, and Perinatal Outcomes Associated with Placental Abruption: A Systematic Review. *Am J Perinatol* 2017; **34**(10): 935-57.

42. Weibel S, Neubert K, Jelting Y, et al. Incidence and severity of chronic pain after caesarean section: A systematic review with meta-analysis. *Eur J Anaesthesiol* 2016; **33**(11): 853-65.
43. Vandenberghe G, Bloemenkamp K, Berlage S, et al. The International Network of Obstetric Survey Systems study of uterine rupture: a descriptive multi-country population-based study. *Bjog* 2019; **126**(3): 370-81.
44. Baradaran K. Risk of Uterine Rupture with Vaginal Birth after Cesarean in Twin Gestations. *Obstet Gynecol Int* 2021; **2021**: 6693142.
45. Tulandi T, Cohen A. Emerging Manifestations of Cesarean Scar Defect in Reproductive-aged Women. *J Minim Invasive Gynecol* 2016; **23**(6): 893-902.
46. Bij de Vaate AJ, van der Voet LF, Naji O, et al. Prevalence, potential risk factors for development and symptoms related to the presence of uterine niches following Cesarean section: systematic review. *Ultrasound Obstet Gynecol* 2014; **43**(4): 372-82.
47. Blondon M, Casini A, Hoppe KK, Boehlen F, Righini M, Smith NL. Risks of Venous Thromboembolism After Cesarean Sections: A Meta-Analysis. *Chest* 2016; **150**(3): 572-96.
48. Yimer H, Woldie H. Incidence and Associated Factors of Chronic Pain After Caesarean Section: A Systematic Review. *J Obstet Gynaecol Can* 2019; **41**(6): 840-54.
49. Wang Y LCY, Yu X., Zhang Y, Xiang. A systematic review of the prevalence and risk factors of chronic pain after cesarean section. *Chinese Journal of Evidence-Based Medicine* 2021; **21**(10): 1195-202.
50. Chen Y, Yang X, Guo C, et al. Prevalence of Post-Traumatic Stress Disorder Following Caesarean Section: A Systematic Review and Meta-Analysis. *J Womens Health (Larchmt)* 2020; **29**(2): 200-9.
51. Marshall NE, Fu R, Guise JM. Impact of multiple cesarean deliveries on maternal morbidity: a systematic review. *Am J Obstet Gynecol* 2011; **205**(3): 262.e1-8.
52. Hartmann K, Viswanathan M, Palmieri R, Gartlehner G, Thorp J, Jr., Lohr KN. Outcomes of routine episiotomy: a systematic review. *Jama* 2005; **293**(17): 2141-8.
53. Frigerio M, Mastrolia SA, Spelzini F, Manodoro S, Yohay D, Weintraub AY. Long-term effects of episiotomy on urinary incontinence and pelvic organ prolapse: a systematic review. *Arch Gynecol Obstet* 2019; **299**(2): 317-25.

Supplementary Table S2. Summary of guidelines and recommendations on long-term conditions of interest

Condition of interest	Number of high-quality guidelines	High-quality guidelines	No. of recommendations* Recommended	Not recommended
**Conditions related to labour and childbirth**				
Genitourinary conditions				
Fistula	1	WHO (2018)[85]	1	0
Pelvic floor disorders	7	CNGOF (2015)[86], CNGOF (2015)[87], CNGOF (2019)[88], DGGG/SGGG/OEGGG (2016)[89], NICE (2019)[90], NICE (2021)[91], NICE (2021)[92]	56	24
Pelvic organ prolapse	5	DGGG, SGGG, OEGGG (2016)[89], EAU (2019)[93], NICE (2019)[90], NICE (2021)[92], SOGC (2021)[94]	68	10
Anal incontinence	4	ASCRS (2015)[95], CNGOF (2015)[87], NICE (2021)[92], WHO (2022)[96]	20	5
Urinary incontinence	9	ACP (2014)[97], NICE (2019)[90], CNGOF (2015)[87], CNGOF (2019)[88], EAU (2019)[93], EAU (2022)[98], NICE (2021)[92], WHO (2022)[96], WPSI (2018)[99]	126	33
Uterine and wound complications	8	CNGOF (2015)[86], WHO (2013)[100], WHO (2022) [96]	8	0
Mental health conditions				
General mental disorders	4	COPE (2017)[101], HIS/SIGN (2012)[102], NICE (2014)[103], NICE (2021)[91]	38	1
Postpartum depression	10	CTFPHC (2013)[104], CTFPHC (2022)[105], COPE (2017)[106], HIS/SIGN (2012)[107], NICE (2014)[108,109], NICE (2017)[109], RNAO (2018)[110], WHO (2017)[111], WHO (2022)[112]	111	10
Anxiety	7	HIS/SIGN (2012)[107], COPE (2017)[95], NICE (2014)[108], RANZCP (2018)[113], WHO (2022)[112], WPSI (2020)[114]	29	1
Post-traumatic stress disorder	2	COPE (2017)[106], NICE (2014)[108]	2	1
Psychosis	3	COPE (2017)[106], HIS/SIGN (2012)[107], NICE (2014)[108]	27	7
Tokophobia	2	NICE (2014)[108], NICE (2021)[115]	2	0
Cardiovascular conditions				
Peripartum cardiomyopathy	2	ACOG (2019)[116] NICE (2019)[117]	19	0
Neurological conditions				
Pain/nerve injury	2	ACOG (2021)[118], CNGOF (2015)[86]	10	0
Endocrine conditions				
Thyroiditis	2	ATA (2016) [119], ATA (2017)[120]	13	2
Mastitis	1	WHO (2022)[112]	2	1

Metabolic conditions				
Weight retention	2	ACOG (2019)[121], WHO (2022)[112]	3	0
Infectious conditions				
HIV seroconversion	4	BHIVA (2019)[122], WHO (2015)[123], WHO (2017)[124], WHO (2022)[112]	18	1
Sepsis	2	WHO (2018)[125], WHO (2022)[112]	0	3
Haematological conditions				
Chronic anaemia	3	CNFOG (2015)[86], DSOG (2016)[126], WHO (2016)[127], WHO (2022)[112]	12	6
Impacts on subsequent pregnancy				
Adverse effects on subsequent pregnancies	1	RCOG (2018)[128]	0	0
Other conditions				
Poorly healed perineum	5	CNGOF (2015)[86], NICE (2017)[109], NICE (2021)[129], WHO (2018)[125], WHO (2022)[112]	33	3
Perineal pain	3	CNGOF (2015)[86], NICE (2021)[91] , WHO (2022)[112]	8	1
Chronic pain	1	EAU (2022)[130]	8	0
Placenta previa or accreta	4	IS-AIP (2019)[131], RCOG (2018)[128], SOGC (2019)[132], SOGC (2020)[133]	75	11
Deep vein thrombosis	4	ACOG (2018)[134], CNGOF (2015)[86], ESVS (2021)[135], WHO (2013)[108]	9	1
Sexual dysfunction	4	CNGOF (2015)[86], NICE (2021)[91], WHO (2013)[136], WHO (2017)[124]	3	1
**Conditions specific to C-section, laparotomy, or hysterectomy**				
Wound complications	2	CNGOF (2015)[86], NICE (2021)[91]	2	0
Pain/nerve injury	1	ACOG (2021)[118]	1	0
Placenta previa or accreta	3	IS-AIP (2019)[131], NICE (2021)[115], SOGC (2019)[132]	33	6
Adverse effects on subsequent pregnancies	3	AAFP (2015)[137], NICE (2019)[117], SOGC (2019)[138]	32	6
**Conditions related to episiotomy or perineal repair**				
Anal incontinence	1	SOGC (2015)[139]	2	0
Pelvic floor disorders	1	CNGOF (2019)[88]	9	4
Wound complications	1	WHO (2018) [125]	0	1
Poorly healed perineum	3	CNGOF (2015)[86], NICE (2017)[109], SOGC (2015)[139]	9	0
**Conditions specific to operative vaginal birth**				
Pelvic floor disorders	2	CNGOF (2019)[88], WHO (2021)[92]	13	1

[*] Five recommendations were statements of insufficient evidence for a particular intervention and were not classified as ‘Recommended’ or ‘Not recommended’.

REFERENCES

1. Shea BJ, Reeves BC, Wells G, et al. AMSTAR 2: a critical appraisal tool for systematic reviews that include randomised or non-randomised studies of healthcare interventions, or both. *BMJ* 2017; **358**: j4008.
2. Banaei M, Kariman N, Ozgoli G, et al. Prevalence of postpartum dyspareunia: A systematic review and meta-analysis. *Int J Gynaecol Obstet* 2021; **153**(1): 14-24.
3. Baradaran K. Risk of Uterine Rupture with Vaginal Birth after Cesarean in Twin Gestations. *Obstet Gynecol Int* 2021; **2021**: 6693142.
4. Bij de Vaate AJ, van der Voet LF, Naji O, et al. Prevalence, potential risk factors for development and symptoms related to the presence of uterine niches following Cesarean section: systematic review. *Ultrasound in obstetrics & gynecology : the official journal of the International Society of Ultrasound in Obstetrics and Gynecology* 2014; **43(4)**: 372-82.
5. Blondon M, Casini A, Hoppe KK, Boehlen F, Righini M, Smith NL. Risks of Venous Thromboembolism After Cesarean Sections: A Meta-Analysis. *CHEST* 2016; **150**(3): 572-96.
6. Chen Y, Yang X, Guo C, et al. Prevalence of Post-Traumatic Stress Disorder Following Caesarean Section: A Systematic Review and Meta-Analysis. *Journal of Women's Health (15409996)* 2020; **29**(2): 200-9.
7. Chen Y, Geng X, Zhou H, et al. Systematic review and meta-analysis of evaluation of selective cesarean section in postpartum pelvic floor function recovery under perineal ultrasound. *Ann Palliat Med* 2022; **11**(2): 730-42.
8. Christopher S, McCullough J, Snodgrass SJ, Cook C. Predictive Risk Factors for First-Onset Lumbopelvic Pain in Postpartum Women: A Systematic Review. *Journal of Women's Health Physical Therapy* 2019; **43**(3): 127-35.
9. Cowgill KD, Bishop J, Norgaard AK, Rubens CE, Gravett MG. Obstetric fistula in low-resource countries: An under-valued and under-studied problem - systematic review of its incidence, prevalence, and association with stillbirth. *BMC Pregnancy and Childbirth* 2015; **15(1) (no pagination)**.
10. De Mucio B, Serruya S, Alemán A, Castellano G, Sosa CG. A systematic review and meta-analysis of cesarean delivery and other uterine surgery as risk factors for placenta accreta. *International Journal of Gynecology & Obstetrics* 2019; **147**(3): 281-91.
11. Dekel S, Stuebe C, Dishy G. Childbirth Induced Posttraumatic Stress Syndrome: A Systematic Review of Prevalence and Risk Factors. *Frontiers in psychology* 2017; **8**: 560.
12. Dennis CL, Falah-Hassani K, Shiri R. Prevalence of antenatal and postnatal anxiety: Systematic review and meta-analysis. *British Journal of Psychiatry* 2017; **210(5)**: 315-23.
13. Downes KL, Grantz KL, Shenassa ED. Maternal, Labor, Delivery, and Perinatal Outcomes Associated with Placental Abruption: A Systematic Review. *Am J Perinatol* 2017; **34**(10): 935-57.
14. Drake AL, Wagner A, Richardson B, John-Stewart G. Incident HIV during pregnancy and postpartum and risk of mother-to-child HIV transmission: a systematic review and meta-analysis. *PLoS medicine* 2014; **11**(2): e1001608.
15. Fawcett EJ, Fairbrother N, Cox ML, White IR, Fawcett JM. The Prevalence of Anxiety Disorders During Pregnancy and the Postpartum Period: A Multivariate Bayesian Meta-Analysis. *J Clin Psychiatry* 2019; **80**(4).
16. Frigerio M, Mastrolia SA, Spelzini F, Manodoro S, Yohay D, Weintraub AY. Long-term effects of episiotomy on urinary incontinence and pelvic organ prolapse: a systematic review. *Arch Gynecol Obstet* 2019; **299**(2): 317-25.
17. Gray TG, Vickers H, Jha S, Jones GL, Brown SR, Radley SC. A systematic review of non-invasive modalities used to identify women with anal incontinence symptoms after childbirth. *Int Urogynecol J* 2019; **30**(6): 869-79.
18. Hahn-Holbrook J, Cornwell-Hinrichs T, Anaya I. Economic and Health Predictors of National Postpartum Depression Prevalence: A Systematic Review, Meta-analysis, and Meta-Regression of 291 Studies from 56 Countries. *Frontiers in psychiatry* 2017; **8**: 248.
19. Hartmann K, Viswanathan M, Palmieri R, Gartlehner G, Thorp J, Jr., Lohr KN. Outcomes of routine episiotomy: a systematic review. *Jama* 2005; **293**(17): 2141-8.

20. Isogai T, Kamiya CA. Worldwide Incidence of Peripartum Cardiomyopathy and Overall Maternal Mortality. *Int Heart J* 2019; **60**(3): 503-11.
21. Kalra H, Tran T, Romero L, Chandra P, Fisher J. Burden of severe maternal peripartum mental disorders in low- and middle-income countries: a systematic review. *Archives of Women's Mental Health* 2022; **25(2)**: 267-75.
22. Keag OE, Norman JE, Stock SJ. Long-term risks and benefits associated with cesarean delivery for mother, baby, and subsequent pregnancies: Systematic review and meta-analysis. *PLoS Med* 2018; **15**(1): e1002494.
23. Korzeniewski R, Kiemle G, Slade P. Mothers' experiences of sex and sexual intimacy in the first postnatal year: a systematic review. *Sexual & Relationship Therapy* 2021; **36**(2/3): 219-37.
24. Liu X, Wang S, Wang G. Prevalence and Risk Factors of Postpartum Depression in Women: A Systematic Review and Meta-analysis. *Journal of clinical nursing* 2021; **08**.
25. Manresa M, Pereda A, Bataller E, Terre-Rull C, Ismail KM, Webb SS. Incidence of perineal pain and dyspareunia following spontaneous vaginal birth: a systematic review and meta-analysis. *International urogynecology journal* 2019; **30**(6): 853-68.
26. Marshall NE, Fu R, Guise JM. Impact of multiple cesarean deliveries on maternal morbidity: a systematic review. *American Journal of Obstetrics & Gynecology* 2011; **205**(3): 262.e1-8.
27. Meng K, Hu X, Peng X, Zhang Z. Incidence of venous thromboembolism during pregnancy and the puerperium: a systematic review and meta-analysis. *Journal of Maternal-Fetal & Neonatal Medicine* 2015; **28**(3): 245-53.
28. Moossdorff-Steinhauser HFA, Berghmans BCM, Spaanderman MEA, Bols EMJ. Prevalence, incidence and bothersomeness of urinary incontinence between 6 weeks and 1 year post-partum: a systematic review and meta-analysis. *Int Urogynecol J* 2021; **32**(7): 1675-93.
29. Nicholson WK, Robinson KA, Smallridge RC, Ladenson PW, Powe NR. Prevalence of postpartum thyroid dysfunction: a quantitative review. *Thyroid : official journal of the American Thyroid Association* 2006; **16**(6): 573-82.
30. Nielsen-Scott M, Fellmeth G, Opondo C, Alderdice F. Prevalence of perinatal anxiety in low- and middle-income countries: A systematic review and meta-analysis. *Journal of affective disorders* 2022; **306**: 71-9.
31. Nilsson C, Hessman E, Sjoblom H, et al. Definitions, measurements and prevalence of fear of childbirth: A systematic review. *BMC Pregnancy and Childbirth* 2018; **18(1) (no pagination)**.
32. O'Connell MA, Leahy-Warren P, Khashan AS, Kenny LC, O'Neill SM. Worldwide prevalence of tocophobia in pregnant women: systematic review and meta-analysis. *Acta Obstetricia et Gynecologica Scandinavica* 2017; **96**(8): 907-20.
33. Rider Sleutel M, True B, Webb J, Valdez E, Van Thi Tran M. Integrative Review of Lower Extremity Nerve Injury During Vaginal Birth. *JOGNN: Journal of Obstetric, Gynecologic & Neonatal Nursing* 2020; **49**(6): 507-24.
34. Shorey S, Chee CYI, Ng ED, Chan YH, Tam WWS, Chong YS. Prevalence and incidence of postpartum depression among healthy mothers: A systematic review and meta-analysis. *Journal of Psychiatric Research* 2018; **104**: 235-48.
35. Sideris M, McCaughey T, Hanrahan JG, et al. Risk of obstetric anal sphincter injuries (OASIS) and anal incontinence: A meta-analysis. *European Journal of Obstetrics and Gynecology and Reproductive Biology* 2020; **252**: 303-12.
36. Smeets CFA, Vergeldt TFM, Notten KJB, Martens FMJ, van Kuijk SMJ. Association between levator ani avulsion and urinary incontinence in women: A systematic review and meta-analysis. *Int J Gynaecol Obstet* 2021; **153**(1): 25-32.
37. Tähtinen RM, Cartwright R, Tsui JF, et al. Long-term Impact of Mode of Delivery on Stress Urinary Incontinence and Urgency Urinary Incontinence: A Systematic Review and Meta-analysis. *Eur Urol* 2016; **70**(1): 148-58.
38. Tulandi T, Cohen A. Emerging Manifestations of Cesarean Scar Defect in Reproductive-aged Women. *Journal of Minimally Invasive Gynecology* 2016; **23**(6): 893-902.
39. VanderKruik R, Barreix M, Chou D, et al. The global prevalence of postpartum psychosis: a systematic review. *BMC psychiatry* 2017; **17**(1): 272.
40. Wang K, Xu X, Jia G, Jiang H. Risk Factors for Postpartum Stress Urinary Incontinence: a Systematic Review and Meta-analysis. *Reprod Sci* 2020; **27**(12): 2129-45.

41. Wang Z, Liu J, Shuai H, et al. Mapping global prevalence of depression among postpartum women. *Translational Psychiatry* 2021; **11(1) (no pagination)**.
42. Wang Y, Liu H, Chen Y, Yu X, Zhang Y, Kong X. Prevalence and risk factors of chronic pain after cesarean section: A systematic review. [Chinese]. *Chinese Journal of Evidence-Based Medicine* 2021; **21(10)**: 1195-202.
43. Weibel S, Neubert K, Jelting Y, et al. Incidence and severity of chronic pain after caesarean section: A systematic review with meta-analysis. *European Journal of Anaesthesiology* 2016; **33(11)**: 853-65.
44. Wilson E, Woodd SL, Benova L. Incidence of and Risk Factors for Lactational Mastitis: A Systematic Review. *Journal of Human Lactation* 2020; **36**(4): 673-86.
45. Woody CA, Ferrari AJ, Siskind DJ, Whiteford HA, Harris MG. A systematic review and meta-regression of the prevalence and incidence of perinatal depression. *J Affect Disord* 2017; **219**: 86-92.
46. Yildiz PD, Ayers S, Phillips L. The prevalence of posttraumatic stress disorder in pregnancy and after birth: A systematic review and meta-analysis. *Journal of Affective Disorders* 2017; **208**: 634-45.
47. Yimer H, Woldie H. Incidence and Associated Factors of Chronic Pain After Caesarean Section: A Systematic Review. *Journal of Obstetrics and Gynaecology Canada* 2019; **41(6)**: 840-54.
48. Brouwers MC, Kho ME, Browman GP, et al. AGREE II: advancing guideline development, reporting, and evaluation in health care. *Prev Med* 2010; **51**(5): 421-4.
49. Maheu-Giroux M, Filippi V, Samadoulougou S, et al. Prevalence of symptoms of vaginal fistula in 19 sub-Saharan Africa countries: a meta-analysis of national household survey data. *Lancet Glob Health* 2015; **3**(5): e271-8.
50. Kiersten J, A. P. Incontinence data from the Demographic and Health Surveys: comparative analysis of a proxy measurement of vaginal fistula and recommendations for future population-based data collection. Calverton, Maryland, USA, 2008.
51. Cowgill KD, Bishop J, Norgaard AK, Rubens CE, Gravett MG. Obstetric fistula in low-resource countries: an under-valued and under-studied problem--systematic review of its incidence, prevalence, and association with stillbirth. *BMC Pregnancy Childbirth* 2015; **15**: 193.
52. Adler AJ, Ronsmans C, Calvert C, Filippi V. Estimating the prevalence of obstetric fistula: a systematic review and meta-analysis. *BMC Pregnancy Childbirth* 2013; **13**: 246.
53. Wu JM, Vaughan CP, Goode PS, et al. Prevalence and trends of symptomatic pelvic floor disorders in U.S. women. *Obstet Gynecol* 2014; **123**(1): 141-8.
54. Sideris M, McCaughey T, Hanrahan JG, et al. Risk of obstetric anal sphincter injuries (OASIS) and anal incontinence: A meta-analysis. *Eur J Obstet Gynecol Reprod Biol* 2020; **252**: 303-12.
55. Mascarenhas MN, Flaxman SR, Boerma T, Vanderpoel S, Stevens GA. National, regional, and global trends in infertility prevalence since 1990: a systematic analysis of 277 health surveys. *PLoS Med* 2012; **9**(12): e1001356.
56. Korzeniewski R KG, Slade P. Mothers' experiences of sex and sexual intimacy in the first postnatal year: a systematic review. *Sexual and Relationship Therapy* 2019; **36**(2-3): 219-37.
57. Manresa M, Pereda A, Bataller E, Terre-Rull C, Ismail KM, Webb SS. Incidence of perineal pain and dyspareunia following spontaneous vaginal birth: a systematic review and meta-analysis. *Int Urogynecol J* 2019; **30**(6): 853-68.
58. Liu X, Wang S, Wang G. Prevalence and Risk Factors of Postpartum Depression in Women: A Systematic Review and Meta-analysis. *J Clin Nurs* 2022; **31**(19-20): 2665-77.
59. Hahn-Holbrook J, Cornwell-Hinrichs T, Anaya I. Economic and Health Predictors of National Postpartum Depression Prevalence: A Systematic Review, Meta-analysis, and Meta-Regression of 291 Studies from 56 Countries. *Front Psychiatry* 2017; **8**: 248.
60. Shorey S, Chee CYI, Ng ED, Chan YH, Tam WWS, Chong YS. Prevalence and incidence of postpartum depression among healthy mothers: A systematic review and meta-analysis. *J Psychiatr Res* 2018; **104**: 235-48.
61. Wang Z, Liu J, Shuai H, et al. Mapping global prevalence of depression among postpartum women. *Transl Psychiatry* 2021; **11**(1): 543.

62. Yildiz PD, Ayers S, Phillips L. The prevalence of posttraumatic stress disorder in pregnancy and after birth: A systematic review and meta-analysis. *J Affect Disord* 2017; **208**: 634-45.
63. Dekel S, Stuebe C, Dishy G. Childbirth Induced Posttraumatic Stress Syndrome: A Systematic Review of Prevalence and Risk Factors. *Front Psychol* 2017; **8**: 560.
64. VanderKruik R, Barreix M, Chou D, Allen T, Say L, Cohen LS. The global prevalence of postpartum psychosis: a systematic review. *BMC Psychiatry* 2017; **17**(1): 272.
65. Kalra H, Tran T, Romero L, Chandra P, Fisher J. Burden of severe maternal peripartum mental disorders in low- and middle-income countries: a systematic review. *Arch Womens Ment Health* 2022; **25**(2): 267-75.
66. Nielsen-Scott M, Fellmeth G, Opondo C, Alderdice F. Prevalence of perinatal anxiety in low- and middle-income countries: A systematic review and meta-analysis. *J Affect Disord* 2022; **306**: 71-9.
67. Dennis CL, Falah-Hassani K, Shiri R. Prevalence of antenatal and postnatal anxiety: systematic review and meta-analysis. *Br J Psychiatry* 2017; **210**(5): 315-23.
68. Nilsson C, Hessman E, Sjöblom H, et al. Definitions, measurements and prevalence of fear of childbirth: a systematic review. *BMC Pregnancy Childbirth* 2018; **18**(1): 28.
69. O'Connell MA, Leahy-Warren P, Khashan AS, Kenny LC, O'Neill SM. Worldwide prevalence of tocophobia in pregnant women: systematic review and meta-analysis. *Acta Obstet Gynecol Scand* 2017; **96**(8): 907-20.
70. Ijaz SH, Jamal S, Minhas AMK, et al. Trends in Characteristics and Outcomes of Peripartum Cardiomyopathy Hospitalizations in the United States Between 2004 and 2018. *Am J Cardiol* 2022; **168**: 142-50.
71. Meng K, Hu X, Peng X, Zhang Z. Incidence of venous thromboembolism during pregnancy and the puerperium: a systematic review and meta-analysis. *J Matern Fetal Neonatal Med* 2015; **28**(3): 245-53.
72. Rider Sleutel M, True B, Webb J, Valdez E, Van Thi Tran M. Integrative Review of Lower Extremity Nerve Injury During Vaginal Birth. *J Obstet Gynecol Neonatal Nurs* 2020; **49**(6): 507-24.
73. Christopher S MJ, Snodgrass S.J., Cook C. Predictive Risk Factors for First-Onset Lumbopelvic Pain in Postpartum Women: A Systematic Review. *Journal of Women's Health Physical Therapy* 2049; **43**(3): 127-35.
74. Nicholson WK, Robinson KA, Smallridge RC, Ladenson PW, Powe NR. Prevalence of postpartum thyroid dysfunction: a quantitative review. *Thyroid* 2006; **16**(6): 573-82.
75. De Mucio B, Serruya S, Alemán A, Castellano G, Sosa CG. A systematic review and meta-analysis of cesarean delivery and other uterine surgery as risk factors for placenta accreta. *Int J Gynaecol Obstet* 2019; **147**(3): 281-91.
76. Vandenberghe G, Bloemenkamp K, Berlage S, et al. The International Network of Obstetric Survey Systems study of uterine rupture: a descriptive multi-country population-based study. *Bjog* 2019; **126**(3): 370-81.
77. Weibel S, Neubert K, Jelting Y, et al. Incidence and severity of chronic pain after caesarean section: A systematic review with meta-analysis. *Eur J Anaesthesiol* 2016; **33**(11): 853-65.
78. Tulandi T, Cohen A. Emerging Manifestations of Cesarean Scar Defect in Reproductive-aged Women. *J Minim Invasive Gynecol* 2016; **23**(6): 893-902.
79. Bij de Vaate AJ, van der Voet LF, Naji O, et al. Prevalence, potential risk factors for development and symptoms related to the presence of uterine niches following Cesarean section: systematic review. *Ultrasound Obstet Gynecol* 2014; **43**(4): 372-82.
80. Blondon M, Casini A, Hoppe KK, Boehlen F, Righini M, Smith NL. Risks of Venous Thromboembolism After Cesarean Sections: A Meta-Analysis. *Chest* 2016; **150**(3): 572-96.
81. Yimer H, Woldie H. Incidence and Associated Factors of Chronic Pain After Caesarean Section: A Systematic Review. *J Obstet Gynaecol Can* 2019; **41**(6): 840-54.
82. Wang Y LCY, Yu X., Zhang Y, Xiang. A systematic review of the prevalence and risk factors of chronic pain after cesarean section. *Chinese Journal of Evidence-Based Medicine* 2021; **21**(10): 1195-202.
83. Chen Y, Yang X, Guo C, et al. Prevalence of Post-Traumatic Stress Disorder Following Caesarean Section: A Systematic Review and Meta-Analysis. *J Womens Health (Larchmt)* 2020; **29**(2): 200-9.

84. Marshall NE, Fu R, Guise JM. Impact of multiple cesarean deliveries on maternal morbidity: a systematic review. *Am J Obstet Gynecol* 2011; **205**(3): 262.e1-8.
85. World Health Organization. WHO Recommendation on Duration of Bladder Catheterization After Surgical Repair of Simple Obstetric Urinary Fistula. WHO Recommendation on Duration of Bladder Catheterization After Surgical Repair of Simple Obstetric Urinary Fistula. Geneva: World Health Organization; 2018.
86. Simon EG, Laffon M. [Maternal care after vaginal delivery and management of complications in immediate post-partum--Guidelines for clinical practice]. *J Gynecol Obstet Biol Reprod (Paris)* 2015; **44**(10): 1101-10.
87. Deffieux X, Vieillefosse S, Billecocq S, et al. [Postpartum pelvic floor muscle training and abdominal rehabilitation: Guidelines]. *J Gynecol Obstet Biol Reprod (Paris)* 2015; **44**(10): 1141-6.
88. Ducarme G, Pizzoferrato AC, de Tayrac R, et al. Perineal prevention and protection in obstetrics: CNGOF clinical practice guidelines. *J Gynecol Obstet Hum Reprod* 2019; **48**(7): 455-60.
89. Baeßler K, Aigmüller T, Albrich S, et al. Diagnosis and Therapy of Female Pelvic Organ Prolapse. Guideline of the DGGG, SGGG and OEGGG (S2e-Level, AWMF Registry Number 015/006, April 2016). *Geburtshilfe Frauenheilkd* 2016; **76**(12): 1287-301.
90. National Institute for Health and Care Excellence (NICE). Urinary incontinence and pelvic organ prolapse in women: management. 2019. https://www.nice.org.uk/guidance/ng123 (accessed 23 October 2022 2022).
91. National Institute for Health and Care Excellence (NICE). Postnatal care. 2021. https://www.nice.org.uk/guidance/ng194/resources/postnatal-care-pdf-66142082148037 (accessed 9 December 2022).
92. National Institute for Health and Care Excellence (NICE). Pelvic floor dysfunction: prevention and non-surgical management. 2021. https://www.nice.org.uk/guidance/ng210 (accessed 23 October 2022 2022).
93. European Association of Urology. Urinary incontinence in adults. 2019. https://d56bochluxqnz.cloudfront.net/media/EAU-Guidelines-on-Urinary-Incontinence-2019.pdf (accessed 23 October 2022 2022).
94. Geoffrion R, Larouche M. Guideline No. 413: Surgical Management of Apical Pelvic Organ Prolapse in Women. *J Obstet Gynaecol Can* 2021; **43**(4): 511-23.e1.
95. Paquette IM, Varma MG, Kaiser AM, Steele SR, Rafferty JF. The American Society of Colon and Rectal Surgeons' Clinical Practice Guideline for the Treatment of Fecal Incontinence. *Dis Colon Rectum* 2015; **58**(7): 623-36.
96. World Health Organization. WHO recommendations on maternal and newborn care for a positive postnatal experience. Geneva, 2022.
97. Qaseem A, Dallas P, Forciea MA, Starkey M, Denberg TD, Shekelle P. Nonsurgical management of urinary incontinence in women: a clinical practice guideline from the American College of Physicians. *Ann Intern Med* 2014; **161**(6): 429-40.
98. Nambiar AK, Arlandis S, Bø K, et al. European Association of Urology Guidelines on the Diagnosis and Management of Female Non-neurogenic Lower Urinary Tract Symptoms. Part 1: Diagnostics, Overactive Bladder, Stress Urinary Incontinence, and Mixed Urinary Incontinence. *Eur Urol* 2022; **82**(1): 49-59.
99. O'Reilly N, Nelson H, Conry J, et al. Screening for Urinary Incontinence in Women: A Recommendation From the Women's Preventive Services Initiative (2018). *Ann Intern Med* 2018; **169**(5).
100. World Health Organization. WHO Recommendations on Postnatal Care of the Mother and Newborn. 2013. https://apps.who.int/iris/handle/10665/97603.
101. Austin M, Highet N. Mental Health Care in the Perinatal Period: Australian Clinical Practice Guideline. . 2017. https://cope.org.au/wp-content/uploads/2017/10/Final-COPE-Perinatal-Mental-Health-Guideline.pdf (accessed 23 October 2022).
102. Healthcare Improvement Scotland, Scottish Intercollegiate Guidelines Network. Management of perinatal mood disorders. 2012. https://www.sign.ac.uk/assets/sign127_update.pdf (accessed 23 October 2022).

103. National Institute for Health and Care Excellence (NICE). Antenatal and postnatal mental health: clinical management and service guidance. 2014. https://www.nice.org.uk/guidance/cg192 (accessed 23 October 2022).
104. Joffres M, Jaramillo A, Dickinson J, et al. Recommendations on screening for depression in adults. *Cmaj* 2013; **185**(9): 775-82.
105. Lang E, Colquhoun H, LeBlanc JC, et al. Recommendation on instrument-based screening for depression during pregnancy and the postpartum period. *Cmaj* 2022; **194**(28): E981-e9.
106. Centre of Perinatal Excellence. Mental Health Care in the Perinatal Period. 2017. https://cope.org.au/wp-content/uploads/2017/10/Final-COPE-Perinatal-Mental-Health-Guideline.pdf (accessed 23 October 2022 2022).
107. Healthcare Improvement Scotland and Scottish Intercollegiate Guidelines Network. Management of perinatal mood disorders. 2012. https://www.sign.ac.uk/assets/sign127_update.pdf (accessed 23 October 2022 2022).
108. National Institute for Health and Care Excellence (NICE). Antenatal and postnatal mental health: clinical management and service guidance. 2014. https://www.nice.org.uk/guidance/cg192 (accessed 23 October 2022 2022).
109. National Institute for Health and Care Excellence (NICE). Intrapartum care for healthy women and babies. 2017. https://www.nice.org.uk/guidance/cg190 (accessed 23 October 2022 2022).
110. Registered Nurses' Association of Ontario. Assessment and interventions for perinatal depression. 2018. https://rnao.ca/sites/rnao-ca/files/bpg/Perinatal_Depression_FINAL_web.pdf (accessed 23 October 2022).
111. World Health Organization. WHO recommendations on maternal health. 2017. https://www.who.int/publications/i/item/WHO-MCA-17.10 (accessed 23 October 2022).
112. World Health Organization. WHO recommendations on maternal and newborn care for a positive postnatal experience. WHO recommendations on maternal and newborn care for a positive postnatal experience. Geneva: World Health Organization; 2022.
113. The Royal Australian and New Zealand College of Psychiatrists. Clinical practice guideline for the treatment of panic disorder, social anxiety disorder and generalised anxiety disorder. 2018. https://www.ranzcp.org/files/resources/college_statements/clinician/cpg/anxiety-cpg.aspx (accessed 23 October 2022).
114. Gregory KD, Chelmow D, Nelson HD, et al. Screening for Anxiety in Adolescent and Adult Women: A Recommendation From the Women's Preventive Services Initiative. *Ann Intern Med* 2020; **173**(1): 48-56.
115. National Institute for Health and Care Excellence (NICE). Caesarean birth. 2021. https://www.nice.org.uk/guidance/ng192 (accessed 23 October 2022).
116. American College of Obstetricians and Gynecologists, Presidential Task Force on Pregnancy Heart Disease Committee. ACOG Practice Bulletin No. 212: Pregnancy and Heart Disease. *Obstet Gynecol* 2019; **133**(5): e320-e56.
117. National Institute for Health and Care Excellence (NICE). Intrapartum care for women with existing medical conditions or obstetric complications and their babies. 2019. https://www.nice.org.uk/guidance/ng121 (accessed 23 October 2022).
118. American College of Obstetricians and Gynecologists. Pharmacologic Stepwise Multimodal Approach for Postpartum Pain Management: ACOG Clinical Consensus No. 1. *Obstet Gynecol* 2021; **138**(3): 507-17.
119. Ross DS, Burch HB, Cooper DS, et al. 2016 American Thyroid Association Guidelines for Diagnosis and Management of Hyperthyroidism and Other Causes of Thyrotoxicosis. *Thyroid* 2016; **26**(10): 1343-421.
120. Alexander EK, Pearce EN, Brent GA, et al. 2017 Guidelines of the American Thyroid Association for the Diagnosis and Management of Thyroid Disease during Pregnancy and the Postpartum. *Thyroid* 2017; **27**(3): 315-89.
121. Maxwell C, Gaudet L, Cassir G, et al. Guideline No. 392-Pregnancy and Maternal Obesity Part 2: Team Planning for Delivery and Postpartum Care. *J Obstet Gynaecol Can* 2019; **41**(11): 1660-75.

122. de Ruiter A, Taylor GP, Clayden P, et al. British HIV Association guidelines for the management of HIV infection in pregnant women 2012 (2014 interim review). *HIV Med* 2014; **15 Suppl 4**: 1-77.
123. World Health Organization. Consolidated Guidelines on HIV Testing Services: 5Cs: Consent, Confidentiality, Counselling, Correct Results and Connection 2015. Consolidated Guidelines on HIV Testing Services: 5Cs: Consent, Confidentiality, Counselling, Correct Results and Connection 2015. Geneva: World Health Organization; 2015.
124. World Health Organization. WHO recommendations on maternal health. 2017. https://www.who.int/publications/i/item/WHO-MCA-17.10 (accessed 23 October 2022 2022).
125. World Health Organization. Intrapartum care for a positive childbirth experience. 2018. https://apps.who.int/iris/bitstream/handle/10665/260178/9789241550215-eng.pdf (accessed 23 October 2022).
126. Danish Society of Obstetrics and Gynecology (DSOG). Anaemia and iron deficiency in pregnancy and postpartum. 2016. http://www.nfog.org/files/guidelines/NFOG_guidelines_DEN_Anaemia%20in%20pregnancy%20and%20post%20partum_2016.pdf (accessed 22 October 2022).
127. World Health Organization. Iron supplementation in postpartum women. 2016. https://apps.who.int/iris/handle/10665/249242 (accessed 23 October 2022).
128. Jauniaux E, Alfirevic Z, Bhide AG, et al. Placenta Praevia and Placenta Accreta: Diagnosis and Management: Green-top Guideline No. 27a. *Bjog* 2019; **126**(1): e1-e48.
129. National Institute for Health and Care Excellence (NICE). Postnatal Care. 2021. https://www.nice.org.uk/guidance/ng194 (accessed 23 October 2022.
130. European Association of Urology. Chronic Pelvic Pain. 2022. https://d56bochluxqnz.cloudfront.net/documents/full-guideline/EAU-Guidelines-on-Chronic-Pelvic-Pain-2022_2022-03-29-084111_kpbq.pdf (accessed 23 October 2022).
131. Collins SL, Alemdar B, van Beekhuizen HJ, et al. Evidence-based guidelines for the management of abnormally invasive placenta: recommendations from the International Society for Abnormally Invasive Placenta. *Am J Obstet Gynecol* 2019; **220**(6): 511-26.
132. Hobson SR, Kingdom JC, Murji A, et al. No. 383-Screening, Diagnosis, and Management of Placenta Accreta Spectrum Disorders. *J Obstet Gynaecol Can* 2019; **41**(7): 1035-49.
133. Jain V, Bos H, Bujold E. Guideline No. 402: Diagnosis and Management of Placenta Previa. *J Obstet Gynaecol Can* 2020; **42**(7): 906-17.e1.
134. American College of Obstetricians and Gynecologists. ACOG Practice Bulletin No. 196: Thromboembolism in Pregnancy. *Obstet Gynecol* 2018; **132**(1): e1-e17.
135. Kakkos SK, Gohel M, Baekgaard N, et al. Editor's Choice - European Society for Vascular Surgery (ESVS) 2021 Clinical Practice Guidelines on the Management of Venous Thrombosis. *Eur J Vasc Endovasc Surg* 2021; **61**(1): 9-82.
136. World Health Organization. WHO Recommendations on Postnatal Care of the Mother and Newborn. WHO Recommendations on Postnatal Care of the Mother and Newborn. Geneva: World Health Organization; 2013.
137. Hauk L. Planning for Labor and Vaginal Birth After Cesarean Delivery: Guidelines from the AAFP. *Am Fam Physician* 2015; **91**(3): 197-8.
138. Dy J, DeMeester S, Lipworth H, Barrett J. No. 382-Trial of Labour After Caesarean. *J Obstet Gynaecol Can* 2019; **41**(7): 992-1011.
139. Harvey MA, Pierce M, Alter JE, et al. Obstetrical Anal Sphincter Injuries (OASIS): Prevention, Recognition, and Repair. *J Obstet Gynaecol Can* 2015; **37**(12): 1131-48.

Supplementary Table S3. Recommended and not recommended practices for medium- and long-term conditions of interest

Table S3.1. Conditions related to labour and childbirth

Condition/Guidelines	Intervention
**Fistula**<br>WHO (2018)[85]	**Recommended**<br>Management - Short duration of bladder catheterization after fistula repair[85]
**Pelvic floor disorders**<br>CNGOF (2015)[86]<br>CNGOF (2015)[87]<br>CNGOF (2019)[88]<br>DGGG/SGGG/OEGGG (2016)[89]<br>NICE (2019)[90]<br>NICE (2021)[140]<br>NICE (2021)[92]	**Recommended**<br>Clinical assessment<br>- Identifying predisposing factors and need for referral[90]<br>- Routine postnatal assessment of bladder and bowel function[140]<br>- Assessment of pelvic floor dysfunction in primary care and excluding possible causes of pelvic floor dysfunction[92]<br>- Clinical assessment for possible causes of pelvic floor dysfunction[92]<br>Screening<br>- Discussing symptoms of pelvic floor dysfunction[92]<br>Prevention<br>- During pregnancy: perineal massage[88], deinfibulation for women with FGM before birth[88]<br>- During labour: choosing maternal position[88], delay in pushing[88], pushing technique[88], manual control of crowning[88]<br>- Pelvic floor muscle training (PFMT) during pregnancy and postnatally (for women who have first-degree relative with pelvic floor dysfunction, or have experienced risk factors during birth[92]), which is supervised by physiotherapist or other healthcare professional with appropriate experience[92].<br>- For women with a history of OASIS: prenatal clinical examination of the perineum[88], and mode of delivery[88]<br>- Postnatal perineal examination to identify OASIS[88]<br>Counselling<br>- Warning about effectiveness of PFMT - duration (1 year) but not long term (6-12 years)[87]<br>- Care planning/person-centered care - discussing pelvic floor dysfunction[92]<br>- How to seek advice from health care provider for women who are self-managing intravaginal device or pessary[92]<br>- Smoking cessation advice for women with mesh implantation[89]<br>Educational<br>- Training for health workers in perineal protection[88] and continuing education in repair of OASIS[88]<br>Management (surgery)<br>- Women with symptomatic stress incontinence and prolapse can be offered simultaneous surgery to treat stress incontinence[89,92], with postoperative application of topical estrogen[89]

	- Repair of external anal sphincter tears[86]<br>- Concomitant sacrocolpopexy and Burch colposuspension[89]<br>- Concomitant suburethral tape insertion (as two-stage procedures[89], and compared to Burch colposuspension for patients with sacrocolpopexy[89]) and vaginal prolapse surgery[89]<br>Management (all other)<br>- Motivation and encouragement for lifestyle changes for women with pelvic floor dysfunction[92]<br>- Lifestyle interventions - diet[92], fluid intake[92], healthy bowel habits[92], reduce caffeine intake[92], modify fluid intake[92], advising women with BMI >30 on weight loss for improving pelvic floor dysfunction[92]<br>- Supervised PFMT - supervised (yoga) or unsupervised (physical activity)[92]<br>- PFMT (preoperative and/or postoperative[89]) - choice of group or individual sessions[92], supervision by therapist (physiotherapist or midwife)[87], clinical review to assess progress during PFMT training program[92], continuation of PFMT training after end of formal training[92].<br>- For women who are unable to perform effective PFMT - supplementing with biofeedback, electrical stimulation or vaginal cones[92],<br>- Psychological impact management for women with pelvic floor dysfunction[92]<br>- Toileting and habit training[92]<br>Health systems<br>- Multidisciplinary care for the management of pelvic floor dysfunction[92]
	**Not recommended**<br>Clinical assessment<br>- Evaluation of cervico-urethral mobility and maximum voluntary contraction of the perineal muscles[87]<br>Prevention<br>- Planned CS for the primary prevention of postnatal urinary or anal incontinence[88], POP and protect sexual functioning[88], for women with a history of OASIS[88], for secondary prevention of urinary incontinence[88]<br>Interventions for prevention of OASIS:<br>- Methods to predict risk - measurement of pelvic dimensions and subpubic angle, length of perineal body or the genital hiatus[88]<br>- Models to predict risk of OASIS to advise/authorise mode of delivery[88]<br>- Epi-No device[88]<br>- PFMT during pregnancy[88]<br>- Fetal rotation for fetuses in posterior cephalic positions to reduce the risk of perineal injury[88]<br>- Specific pushing technique and maternal position in labour to prevent OASIS[88]<br>Management<br>- Surgery - Preoperative placement of ureteral stents[89] and multifilament mesh for prolapse repair[89]

	- PFMT – early rehabilitation within 2 months of childbirth[87], PFMT in asymptomatic women for preventing incontinence[87]<br>- Early rehabilitation, preventive rehabilitation and different techniques[87],<br>- Electrostimulation of the pelvic floor[87]<br>- Vaginal cones as a postpartum rehabilitation technique[87]<br>- Pharmacotherapy – vaginal diazepam to treat pelvic floor dysfunction[92]<br>- Waiting for women to lose weight before starting other pelvic floor management options [92]
**Pelvic Organ Prolapse**<br>DGGG, SGGG, OEGGG (2016)[89]<br>EAU (2019)[93]<br>NICE (2019)[90]<br>NICE (2021)[92]<br>SOGC (2021)[94]	**Recommended**<br>Clinical assessment<br>- Assessing symptoms of vaginal prolapse (by specialist evaluation[90]) in primary care[90]<br>Diagnostic procedures<br>- Using a validated questionnaire to assess pelvic organ prolapse[90] and repeat examination for women with symptoms not explained by findings of physical examination[90]<br>- Pelvic floor sonography as a useful diagnostic tool[89], vaginal imaging prior to and after surgery and to exclude extrauterine pelvic pathologies[89]<br>- Dynamic MRI in assessment of complex conditions[89]<br>- Urodynamic studies prior to planned prolapse surgery[89]<br>- Cystourethroscopy to exclude morphological causes in the presence of bladder pain and hematuria[89]<br>Counselling<br>- Counselling about risks of surgical correction and surgical outcomes with apical POP[94]<br>- Informed decision-making for treatment options for women with uterine prolapse, pelvic organ prolapse, and vault prolapse[90]<br>- Surgery - informed decision-making[90], use of synthetic meshes compared to anterior vaginal wall repair[89], concurrent surgery for stress urinary incontinence and pelvic organ prolapse[89], and risks of combined surgery compared to prolapse surgery alone for women with pelvic organ prolapse[93]<br>- Treatment options for women with anterior prolapse[90]<br>Management<br>- PFMT as first-line treatment for women with pelvic organ prolapse[90] and for lower stages of prolapse (Stages I and II)[89]<br>- Pessary use: alone or in conjunction with PFMT[90], suitable pessary types[89]; considerations before use[90,92], use for women with symptomatic pelvic organ prolapse[92], follow up review for women at risk of complications[90]; topical vaginal estrogen as an adjunct therapy[89]<br>- Renal sonography in women with high-grade prolapse[89]<br>- Cystourethroscopy at the end of prolapse surgery[89]<br>- Observation alone as a management option[89]<br>- Perioperative pelvic floor rehabilitation[89]<br>Management (surgery)

	- Choice of procedure for women with vault prolapse[90], anterior vaginal wall prolapse[90], or uterine prolapse[90], and for women who have no preference about uterine preservation[90]<br>- Surgery in the context of non-improvement or decline[90]<br>- Informed decision-making regarding risk of postoperative incontinence[90]<br>- Considerations for colpocleisis[90],[89], women with levator defects[89], uterine preservation[89]<br>- Considerations before synthetic polypropylene or biological mesh insertion for women with recurrent anterior vaginal wall prolapse[90]<br>- Posterior vaginal repair without mesh compared to transanal rectocele repair for women with a symptomatic rectocele[89] for women with a posterior vaginal wall prolapse[90]<br>- Postoperative follow up after surgery for pelvic organ prolapse[90]<br>- Concurrent surgery for stress urinary incontinence and pelvic organ prolapse[90]<br>- Concomitant apical fixation and anterior vaginal wall repair for women with anterior and middle prolapse[89]<br>- Concomitant placement of synthetic mesh in the anterior compartment and apical mesh fixation[89]<br>- Use of synthetic meshes compared to anterior vaginal wall repair[89]<br>- Options for repair of middle compartment prolapse[89]<br>- Sacrocolpopexy and sacrospinous fixation procedures[89]<br>- Minimally invasive laparoscopic or robotic approaches to sacrocolpopexy compared to open sacrocolpopexy[94]<br>- Intraoperative cystoscopy for vaginal vault suspension to the uterosacral ligaments[89]<br>- Anterior vaginal wall repair compared to transobturator mesh replacement in preoperatively continent women[89]<br>- Vaginal uterosacral ligament suspension and sacrospinous fixation[94]<br>- Hysteropexy as an alternative to hysterectomy and suspension for women with apical pelvic organ prolapse who wish to conserve their uterus[94]<br>- Risks of combined surgery compared to prolapse surgery alone for women with pelvic organ prolapse[93]<br>- Concomitant surgery for pelvic organ prolapse and SUI[93]<br>- PFMT - supervision and duration for women with symptomatic pelvic organ prolapse[92]
	**Not recommended**<br>Diagnostic procedures<br>- Routine imaging to document presence of vaginal prolapse following identification by physical examination[90]<br>Management<br>- Offering surgery to prevent incontinence in women having surgery for prolapse who do not have incontinence.[90]<br>- Hormonal replacement therapy in the treatment of prolapse or incontinence[89]<br>Management (surgery):<br>- Anterior vaginal wall repair compared to biological implants[89]<br>- Synthetic meshes for primary repair of the posterior compartmment[89]<br>- Concomitant hysterectomy and scarocolplexy[89]; biological implants in sacrocolplexy[89]

	- Vaginal high levator myorrhaphy and vaginal fixation of the vaginal vault to the fascia of the iliococcygeus muscle[89]
**Anal incontinence**<br>ASCRS (2015)[95]<br>CNGOF (2015)[87]<br>NICE (2021)[92]<br>WHO (2022)[112]	**Recommended**<br>Clinical assessment<br>- Assessing bowel function at postnatal contacts[112]<br>- Taking history from women with fecal incontinence symptoms[95]<br>Diagnostic procedures<br>- Use a validated quality of life measure as part of clinical assessment[95]<br>- Anorectal physiology testing[95]<br>- Endoscopic evaluation[95], endoanal ultrasound in women with suspected sphincter injury[95]<br>Management<br>- Dietary and medical management as first-line therapy[95]<br>- Bowel management programs[95]; PFMT[87], biofeedback[95]<br>- Surgery: for anatomical defects[95], sacral neuromodulation surgery[95], colostomy[95]<br>- Anorectal physiology testing[95]<br>- Percutaneous tibial nerve stimulation[95]<br>- Short duration of bladder catheterization after fistula repair[92]<br>- Injection of bulking agents into anal canal[95]<br>- Temperature-controlled radiofrequency energy application[95]<br>- Artificial bowel sphincter[95]
	**Not recommended**<br>Diagnostic procedures<br>- Routine pudendal nerve terminal motor latency testing[95]<br>Management<br>- Repeat anal sphincter reconstruction after a failed over-lapping sphincteroplasty, unless other treatment modalities are not possible or have failed[95]<br>- Plication of external anal sphincter[95]<br>- Magnetic sphincter[95]
**Urinary incontinence**<br>ACP (2014)[97]<br>AUA/SUFU (2014)[141]<br>CNGOF (2015)[87]<br>EAU (2019)[93]<br>EAU (2022)[98]<br>NICE (2019)[90]<br>NICE (2021)[92]	**Recommended**<br>Clinical assessment<br>- Assessing urinary function at postnatal contacts[112]<br>- Bladder diaries (duration of 3 days[93]) and pad testing[90] as part of assessment[93],[90]<br>- Clinical examination of women with urinary symptoms[87]<br>- Routine digital assessment before PFMT for treatment of UI[90]<br>- Urinalysis as part of initial assessment of UI[93]<br>- Classification of mixed urinary incontinence (MUI)[98]

WHO (2022)[112]<br>WPSI (2018)[142]	Diagnostic procedures<br>- *In women with SUI*: assessing post-void residual urine volume where invasive therapy is being considered[141]; multi-channel urodynamics or repeat stress testing with catheter removed in women with suspected SUI[141]<br>- Assessing urethral function as part of diagnosis of urodynamic SUI[141]<br>- Bladder blue filling test or urine dipstick test for investigation of urinary fistula, UTI or overflow urination[87]<br>- Diagnosis of postpartum UI based on history[87];<br>- Cystometry in women where invasive treatments being considered[141];<br>- Pad test for quantification of UI[98] with standardized duration/activity protocol[98]<br>- Use a validated urinary incontinence-specific symptom and quality-of-life questionnaire when therapies are being evaluated[90]<br>- Pressure flow studies (PFS) in women to determine if obstruction is present[141] and in patients with urgency incontinence after bladder outlet procedures[141]<br>- Preoperative urodynamic tests in cases of SUI with associated storage symptoms[98]<br>- Postvoid residual (PVR) in patients with lower urinary tract symptoms (LUTS)[141]<br>- Reassess woman with urinary incontinence (UI) after treatment of urinary tract infection (UTI)[93]<br>- Repeat stress testing with catheter removed in suspected stress urinary incontinence (SUI)[141]<br>- Stress testing with reduction of the prolapse in women with high grade pelvic organ prolapse (POP) but without SUI symptoms[141]<br>- Urodynamics testing when it may affect choice of invasive treatment[93]<br>- Video urodynamics in properly selected patients to localize the level of obstruction[141]<br>Counselling<br>- Counselling women on treatment options for UI[98], success rates of surgery for MUI[98], risks and benefits of artificial urinary sphincter or adjustable compression device[98], on risks and benefits of mid-urethral sling[98], that vaginal surgery can affect sexual function[93]<br>- Counselling obese and older women with SUI on risks and benefits of surgery[98]<br>- *For women with SUI:* counselling on intramural bulking agents and use a patient decision aid on surgery[90]<br>- Offering different surgical procedures for patients with SUI where conservative treatment has failed[98]<br>- Counselling women on systemic estradiol with UI that ceasing estradiol unlikely to improve incontinence[93]<br>- Counselling women receiving AUS or ACT on risks and benefits[93]<br>- Counselling women that long-term efficacy of single-incision sling is uncertain[93]<br>- Bowel management advice for adults with UI who experience constipation[93]<br>- Counselling patients with UI and MI on absence of DO on single urodynamic study[141]<br>Management<br>- Annual screening of women for urinary incontinence[142] and using validated questionnaire for standardized assessment of urinary symptoms[93]

	- Supported bladder training[92] in women with urgency UI[97,98]<br>- PFMT with bladder training in UI/SUI[97],[98]/MUI[90,93,98,143] and persistent UI[87] (intensity/frequency of PFMT program[93],[90]) in combination with electrical stimulation[90]<br>- Intramural bulking agents[93]<br>- *Pharmacotherapy:* drug treatment for women with UUI if bladder training is unsuccessful[97]; hormone replacement therapy - discuss alternative therapies of UI develops of experience worsens[93]; long-term vaginal estrogen therapy to postmenopausal women with UI[93]; desmopressin for short-term relief of daytime UI[93] and sodium level monitoring for patients on desmopressin[93]<br>- Trial of intravaginal devices if non-surgical options are unsuccessful[92]<br>- Lifestyle interventions: reducing caffeine intake[93]; smoking cessation[93]; weight loss and exercise for overweight and obese women with UI[90],[97],[93,98]/OAB[93,98] or LUTS/SUI[98]; modification of fluid intake for women with UI or OAB[93],[90]<br>- Incontinence pads or containment devices for UI[93],[98] and annual reviews of women using absorbent containment products[90]<br>- New SUI with pessary use should offer treatment or removal of pessary[92]<br>- *Surgery*: first options for complicated SUI[93] and selecting approach based on careful evaluation of the individual patient including multichannel urodynamics and imaging[93]<br>- *For patients with SUI*: ensuring further referrals for women having recurrent symptoms or complications after SUI[90]; Mid-urethral sling in women with uncomplicated SUI[93] or seeking surgery for SUI[98], specifically surgical approach[98] and vaginal examination at post-operative follow-up[90] for retropubic mesh with specific sling type and colour[90]; vaginal estrogen therapy for postmenopausal women[98]; balancing risks and benefits of PFMT and invasive surgery for SUI[98]; multidisciplinary care for women where primary surgery for SUI has failed or symptoms recurred[90]; postoperative follow up of women after surgery for SUI[90] and women with artificial urinary sphincter[90]<br>- *For patients with MUI*: treating worst symptoms first[98]; anticholinergic drugs or beta 3 agonists for patients with urgency-predominant MUI[98]; duloxetine to select patients with stress-predominant MUI that is unresponsive to conservative treatment[9,98]<br>- *For patients with OAB/UUI:* augmentation cystoplasty[98] or urinary diversion[98] where other treatments have failed; sacral nerve stimulation for patients who are refractory to anticholinergic medication[98]; percutaneous tibial nerve stimulation in women who have not benefited from anticholinergic medication[98]<br>- *For women seeking surgery for SUI:* autologous sling placement[98]; colposuspension[98]; urethral bulking agents[98]<br>- Indications for referring women with UI to a specialist service[90]<br>Health systems<br>- Health staff performing annual reviews of women using absorbent containment products[90]<br>- Management of complicated SUI in expert centres[93]
	**Not recommended**<br>Clinical assessment<br>- Routine urodynamics testing for uncomplicated SUI[93,98]

	Prevention<br>- Routine PFMT after childbirth for prevention of incontinence[112]<br>Diagnostic procedures<br>- Additional examinations in women with normal history and examination, no signs of UTI or retention[87]<br>- Cystoscopy in initial assessment[90] and imagine for routine assessment[90] of women with UI<br>- Multichannel filling and voiding cystometry before primary surgery for SUI[90],[90]<br>- Tests for urethral competence in women with UI[90]<br>- Urethral pressure profilometry or leak point pressure for grading incontinence severity[93]<br>- *For UI:* “stop peeing” exercises during postpartum rehabilitation[87]; absorbent containment products, handheld urinals or toileting aids as treatment for UI[90]; desmopressin for long-term control[93]; postpartum Pilates or osteopathy[87]; abdominal re-education in addition to perineal re-education during postpartum[87]<br>- *For SUI:* electric stimulation with surface electrodes[93,98]; systemic pharmacologic therapy[97]; alternative surgical procedures[90]; artificial urinary sphincter[90]<br>- *For UI and/or OAB:* magnetic stimulation[93]; complementary therapies[90]<br>- Autologous fat and hyaluronic acid as urethral bulking agents[98]<br>- Detrusor myomectomy for UUI[98]<br>- Intravaginal and intraurethral devices for the routine management of UI[90]<br>- Perineometry or pelvic floor electromyography as biofeedback as a routine part of PFMT[90]<br>- Surgical approach for retropubic mid-urethral mesh sling procedure[90]
**Uterine and wound complications**<br>CNGOF (2015)[86]<br>WHO (2013)[136]<br>WHO (2022)[112]	**Recommended**<br>Clinical assessment<br>- Clinical assessment at postnatal contact for signs of uterine or wound complications[112]<br>Diagnostic procedures<br>- Clinical diagnosis of postpartum endometritis based on pelvic pain, hyperthermia and fetid lochia[86]<br>- Imaging investigations for postnatal women with persistent fever despite antibiotics[86]<br>Management<br>- First-line antibiotic regimens for postpartum endometritis[86]<br>- Heparin therapy for endometritis-associated pelvic thrombophlebitis[86]<br>- Antibiotic prophylaxis after 3rd or 4th degree tear[136]<br>- Oral NSAID therapy for postpartum pain due to uterine cramping[112]
**General mental disorders**<br>COPE (2017)[106]<br>HIS/SIGN (2012)[107]<br>NICE (2014)[108]	**Recommended**<br>Screening<br>- Clinical assessment of emotional well-being at antenatal and postnatal contacts[106]<br>- Contact, support and monitoring[108]<br>Clinical assessment

NICE (2021)[140]	- Clinical assessment of women presenting with suspected mental health problem in pregnancy or postnatal[108]<br>- Asking women about signs and symptoms of perinatal mental health issues in perinatal period[140]<br>Person-centered care<br>- Supporting women on psychotropic medication to make informed choices about breastfeeding[107]<br>Counselling<br>- Culturally relevant information to women with mental health problems in pregnancy and postpartum[108]<br>- Counselling women on mental health problems during the perinatal period[106]; risks and benefits of treatment options[106],[108] specifically during breastfeeding[106],[108]; relapse risk[106]<br>- Involvement of significant other(s) in discussions about a woman's emotional well-being[106]<br>Educational<br>- Healthcare professional knowledge of perinatal mental health presentation and clinical course[108]<br>- Healthcare professional training on communication, psychosocial assessment and culturally safe care[106]<br>Health systems<br>- Stepped-care model of service delivery[108]; care pathways[108] and clinical networks[108] for perinatal mental health issues<br>- Admission to mother/baby unit for postpartum women needing inpatient mental health care[108],[106]<br>- Staffing and capacity of specialist perinatal inpatient services[108]<br>Management<br>- Ensuring supports are in place for women who elect to cease psychotropic medication[108]<br>- Seeking specialist advice from perinatal mental health services on choice of psychotropic medication[108]<br>- Relative risks and benefits of different antidepressants[108]<br>- Considering learning disabilities or acquired cognitive impairments in women with mental health issues during pregnancy or postpartum[108]<br>- Performing risk assessments[108]<br>- Safeguarding protocols when child maltreatment is suspected[108]<br>- Actions to take when risk of self-harm or suicide is present[108]<br>- Written care plans for women with severe mental illness[108]<br>- Delivery of perinatal mental health services and interventions by trained professionals[108]<br>- Timing of assessment and psychological intervention in women referred with known/suspected mental health problems in pregnancy or postpartum period[108]<br>- Regular review of psychotropic medication regimen[108]<br>- Encouraging breastfeeding in women with mental health problems, except when contraindicated due to medications[108]<br>- Contact, support and monitoring[108]<br>- Including baby in assessment, care and treatment of postnatal woman with mental health problem[106]<br>- Early postnatal review of psychotropic medication regimen used during pregnancy[106]
	**Not recommended**

	- Using tools for detection of depression, in order to detect other mental health problems[107]
**Postnatal depression**<br>CTFPHC (2013)[104]<br>CTFPHC (2022)[105]<br>COPE (2017)[106]<br>HIS/SIGN (2012)[107]<br>NICE (2014)[108,109]<br>NICE (2017)[109]<br>RNAO (2018)[144]<br>WHO (2017)[124]<br>WHO (2022)[112]	**Recommended**<br>Prevention<br>- Formal debriefing for all women to reduce occurrence/risk of postpartum depression[136,124]<br>- Printed educational materials for prevention of postpartum depression[136],[124]<br>- Psychosocial support and promoting self-care for women at high risk of postnatal depression[144],[124]<br>Diagnostic procedures<br>- Referral / further assessment for comprehensive perinatal depression assessment for persons who screen positive for perinatal depression[144]<br>Clinical assessment<br>- Normal emotional changes of perinatal period masking depression[107]<br>- Assessing woman's emotional and psychological condition after birth[136],[109]<br>Screening<br>- Early first screening and repeat screening in pregnancy for depression[106]<br>- Routine screening for depression in antenatal and postpartum women[144],[145]<br>- Enquiring on depressive symptoms at booking and postnatally in all women[107]<br>- Use of a validated screening tool[112] (GAD-2[108], EPDS[106],[107], [108],[146], [109], PHQ-9[108],[146], [109], Whooley[107], [146]) or referral to GP[109] in screening and monitoring for depression[109]<br>- Further assessment/referral based on EPDS score[106] and suicidality risk in the perinatal period[106]<br>- Re-evaluating women in whom depression is suspected[107]<br>- Asking women about resolution of maternal blues at 10-14 days postnatal[124,136]<br>- Culturally appropriate screening for depression[106]<br>Counselling<br>- Counselling for postpartum depression or anxiety[106]<br>- Informed decision-making and advocate for access to pharmacological interventions for perinatal depression[144]<br>- Counselling women on benefits of psychological interventions for depression and anxiety[106]<br>- Counsel women on risks of complementary therapies for perinatal depression[144]<br>Prevention<br>- Psychosocial and/or psychological interventions to prevent postpartum depression and anxiety[112]<br>Management (non-pharmacological):<br>- Psychoeducation for women with symptoms of perinatal depression[106]/at risk of perinatal depression[144]<br>- Psychotherapy for persons at risk of perinatal depression[144], or for mild to moderate postpartum depression[106,146]<br>- Counselling interventions for women at increased risk of perinatal depression[147]<br>- CBT for mild-moderate postnatal depression[107,148]<br>- Social support groups for women with postnatal depression symptoms[106]

	- Facilitated self-help in women with subthreshold, mild or moderate perinatal depression[109]<br>- Structured exercise for postnatal depression[107]<br>- Self-help for depression in the perinatal period[106]<br>- Evaluate and revise plan of care for perinatal depression[144]<br>- Mother-infant relationship interventions[106]<br>- Urgent mental health assessment, support and treatment when risk of suicide is high[106]<br>Management (pharmacological):<br>- For women with moderate or severe perinatal depression: antidepressants[109], non-directive counselling[148], psychological interventions[109]<br>- Alternative antidepressant options for depression[108]<br>- *SSRI use:* for moderate[106] to severe postnatal depression[146], choice based on infant health and age[106]<br>- TCA, and (S)NRI[109] for severe postnatal depression, with counselling on breastfeeding[107]<br>- Supporting women with depression or anxiety who stop taking psychotropic medication in pregnancy or postpartum[109]<br>- ECT indication: severe postnatal depression where antidepressants have failed[106], or where risk of suicide or level of distress is high[106]<br>Person-centered care<br>- Person-centered plan of care for those who screen positive for perinatal depression[144]<br>- Opportunity for women to discuss their birth experience during their hospital stay[136]<br>Health systems<br>- Education programs for undergraduate nurses and allied health professionals on perinatal depression[144]<br>- Health professionals: self-reflection on perinatal depression beliefs[144]; education on available mental health services and supports[144]; competency and supervision in providing mental health care[107]<br>- Health systems prioritizing early response for pregnant and postnatal women[107]
	**Not recommended**<br>Screening<br>- Routine screening for depression in adults at increased risk of depression[104]<br>- Instrument-based depression screening using a cut-off score[105]<br>Management<br>- Estrogen therapy in postnatal depression[107]<br>- Complementary therapy during pregnancy and lactation[107]<br>- Choice of antidepressants in women who are breastfeeding[107]<br>- Psychosocial assessment in antenatal period for purpose of identifying risk of postnatal depression[107]<br>- Omega-3 fatty acid supplementation for women with depression[106]
**Anxiety**<br>COPE (2017)[95]	**Recommended**<br>Clinical assessment

HIS/SIGN (2012)[107]<br>NICE (2014)[108]<br>RANZCP (2018)[149]<br>WHO (2022)[112]<br>WPSI (2020)[114]	- Clinical assessment of women in perinatal period[106,114]<br>Screening<br>- Screening using validated screening tool to identify postpartum anxiety[112] including GAD-2[87] (referral of women with high scores[118]), EPDS[106,148], DASS[106], K-10[106], ANRQ[106], and WPSI[114]<br>Management<br>- Multimodal management of anxiety[149] and reviewing response to anxiety management[108],[149]<br>- Facilitated self-help interventions for women subthreshold anxiety[136] and counselling women on benefits of psychological interventions[106]<br>- Postnatal choice of SSRI's based on infant health and age[106]; alternative antidepressant options for anxiety[108,124]<br>Prevention<br>- Psychosocial support[107,108,112] and/or psychological interventions[112]
	**Disagreement between guidelines**<br>Management: Use of benzodiazepines during pregnancy or breastfeeding [106-108,124,133]
**PTSD**<br>COPE (2017)[106]<br>NICE (2014)[108]	**Recommended**<br>Psychological interventions[108] or counselling[106] for women with PTSD
	**Not recommended**<br>Single-session psychological interventions for women with PTSD focused on reliving trauma[108]
**Psychosis**<br>COPE (2017)[106]<br>HIS/SIGN (2012)[107]<br>NICE (2014)[108]	**Recommended**<br>Clinical assessment<br>- Taking mental health history at postnatal contacts[107,108]<br>- Monitoring high-risk women for any symptom onset[107,108]<br>Counselling<br>- Counselling women who decide to stop psychotropic medication[108]<br>Management<br>- Anticonvulsants in pregnant and postpartum women[106],[108] and monitoring of lamotrigine levels[107,108]<br>- Choice of antipsychotic medications based on safety profile and breastfeeding[107]<br>- Referral to specialist perinatal mental health service[107],[108]<br>- Shared-decision making with women requiring mood stabilizer therapy and breastfeeding[107]<br>- Psychiatric plan with multidisciplinary care team for women at high risk of postnatal major mental illness[107]<br>- Mental health risk assessment based on family history[107]<br>- Ceasing psychotropic medication when planning pregnancy[107]<br>- Long-acting contraception for women taking valproate and where no alternative is available[107]<br>- Antidepressant dosing in breastfeeding women[107]<br>Counselling<br>- Treatment principles when prescribing psychotropic medication during pregnancy or breastfeeding[107]

	- Counselling women taking psychotropic medication and are of reproductive age of their potential effects on pregnancy, and contraceptive use[107]
	**Not recommended**<br>Management<br>- Valproate for women of childbearing age[107] for acute or long-term treatment[108]<br>- Carbamazepine for mental health conditions[108] and depot antipsychotics[108]<br>Health systems<br>- Short duration of bladder catheterization after fistula repair[107]
	**Disagreement between guidelines**<br>- Lithium/clozapine in breastfeeding women[106,107]
**Tokophobia**<br>NICE (2014)[108]<br>NICE (2021)[150]	**Recommended**<br>- Referral to mental health support services[108,150]
**Peripartum cardiomyopathy**<br>ACOG (2019)[151]<br>NICE (2021)[152]	**Recommended**<br>Counselling<br>- Counselling women with cardiovascular disease diseases on risks[152]<br>Clinical assessment<br>- Identifying women with heart disease[151] and investigations for women with clinical suspicion of intrapartum heart failure[151]<br>- Testing cardiac function[152] and baseline BNP level[152] for high-risk women<br>Screening<br>- Assessing for cardiovascular disease in postpartum period using an algorithm[152]<br>- Echocardiogram in postpartum women history of heart disease[152]<br>Diagnosis<br>- Evaluating symptomatic women for peripartum cardiomyopathy[152]<br>Educational<br>- Healthcare provider knowledge of cardiovascular disease presentations in postpartum women[152]<br>Management<br>- Intrapartum monitoring[151], timing of birth[151], and mode of birth[152] in women with cardiovascular disease<br>- Management of suspected heart failure should include cardiologist review, transthoracic echocardiogram, and NT-proBNP level measurement in the intrapartum period[151], and continued involvement of a cardiologist after birth[151]<br>- Postpartum follow-up of women with cardiovascular diseases[152]<br>Health systems<br>- Multidisciplinary team care[152] and referral to higher-level care[152] for women with cardiovascular diseases
**Pain/nerve injury**<br>ACOG (2021)[118]	**Recommended**<br>- Positioning of women in delivery room to prevent neuropathies[86]

CNGOF (2015)[86]	- Imaging in women with postpartum sensorimotor deficits without a clear diagnosis[86]<br>- Pain management includes shared decision-making with women[118] and a multimodal approach[118]<br>- Shortest duration possible of opioid therapy for acute pain management[118]
**Thyroiditis**<br>ATA (2016)[119]<br>ATA (2017)[120]	Recommended<br>Counselling<br>- Discussing risks and future pregnancies in women with hyperthyroidism[119]<br>Screening<br>- Screening women with postpartum depression for thyroid dysfunction[120] and investigations for postpartum thyrotoxicosis[119]<br>- Annual TSH testing for women with history of postpartum thyroiditis[120]<br>- BWPS score guiding treatment in women with thyroid storm[119]<br>Management<br>- Symptomatic treatment of postpartum thyroiditis with beta-blockers[119,120]<br>- Measuring TSH levels following thyrotoxic phase[120]<br>- LT4 treatment for women with postpartum thyroiditis[120]<br>- Multimodal therapy for patients with thyroid storm[119]
	Not recommended<br>- Antithyroid drugs in thyrotoxic phase[120]<br>- Treatment of euthyroid Ab-positive women with LT4 or iodine to prevent postpartum thyroiditis[120]
**Mastitis**<br>WHO (2022)[112]	Recommended<br>- Assess for breast pain at each postnatal contact after 24 hours[112]<br>- Breastfeeding counselling for preventing mastitis[112]
	Not recommended<br>- Routine oral or topical antibiotics for prevention[112]
**Weight retention**<br>ACOG (2019)[121]<br>WHO (2022)[112]	Recommended<br>- Counselling regarding postpartum weight management[121]<br>- Regular physical activity in postpartum women[112]
**HIV seroconversion**<br>BHIVA (2019)[122]<br>WHO (2015)[123]<br>WHO (2017)[124]<br>WHO (2022)[112]	Recommended<br>Clinical assessment<br>- Postpartum medical assessment of women with HIV who deliver outside a health facility[124]<br>Context-specific screening<br>- Antenatal and postnatal provider-initiated testing and counselling in high prevalence settings (including catch-up testing if needed)[112,123]<br>Screening<br>- Re-testing HIV-negative women who are in a sero-discordant couple or from a key population group[123]<br>- Re-testing for HIV in third trimester, during labour or immediate postnatal period in epidemic settings[123],[124]

	- Antenatal provider-initiated testing and counselling in low-prevalence settings[123]<br>- Postnatal provider-initiated testing and counselling in high prevalence settings[123]<br>- HIV testing of late-presenting women without a HIV result[122]<br>- HIV resistance testing prior to ART therapy, except for late-presenting women[122]<br>Management (postpartum care for HIV-positive women):<br>- Continuation of ART therapy[122,124]; discontinuation of NNRTI therapy[122]<br>- Follow-up postpartum care for HIV women includes linkages, safe infant feeding, ARV coverage & adherence, early infant diagnosis testing, family planning [124]<br>- Infant feeding for women with HIV: counselling on infant formula feeding[122,124]; intensive support and monitoring (monthly maternal and infant testing)[122]
	**Not recommended**<br>- Automatic referral to child protection of women with HIV on cART with repeatedly undetectable viral load, who choose to breastfeed[122]
**Sepsis**<br>WHO (2018)[153]<br>WHO (2022)[112]	**Not recommended**<br>- Routine antibiotics for prophylaxis after vaginal birth[112,153]
**Chronic anaemia**<br>CNFOG (2015)[86]<br>DSOG (2016)[154]<br>WHO (2016)[155]<br>WHO (2022)[112]	**Recommended**<br>- Assess women for anaemia when symptomatic[86,154] or postpartum blood loss >1000mL[86,154]<br>- Postnatal treatment for anaemia:[120] Oral iron supplementation[86,112,154,155] and blood transfusion[86,154] and injectable iron[86] when anaemia is severe
	**Not recommended**<br>- Assessing for anaemia postpartum in women with no risk factors[86] or when blood loss 500-1000mL[154]<br>- Postnatal IV iron supplementation for iron deficiency anaemia, or anaemia due to PPH[154]
**Poorly healed perineum**<br>CNGOF (2015)[86]<br>NICE (2017)[109]<br>NICE (2021)[156]<br>WHO (2018)[153]<br>WHO (2022)[112]	**Recommended**<br>- During second stage of labour, use hands on/hands poised technique[109,153], warm compresses[153]<br>- Counselling women with a history of perineal trauma on risk of perineal trauma in current birth[109] and women with history of 3rd/4th degree tear on mode of birth[109]<br>- Perineum hygiene advice for postpartum women[85]<br>- Instrumental birth or suspected fetal compromise as indications for episiotomy[109]<br>- Assessment (systematic[109,140]) for genital tract trauma[109] (obstetric-led care review[109,140]), including timing of assessment[109], maternal position during assessment[109], and documentation in clinical notes[109]<br>- Health professional training on appropriate perineal/genital assessment and repair[109]<br>- Performing a perineal repair - timing[109], pain management[109] and technique (suturing)[109]<br>- Referral to specialist care for women with poorly hearing perineum[140]<br>- Wound suturing for 1st degree perineal tear[109]

	- Perineal infection management[86], antibiotic therapy[86], and taking bacteriological samples[86]
	**Not recommended**<br>- Routine episiotomy for vaginal birth[86] and after 3rd or 4th degree tear[86]
	**Disagreement between guidelines**<br>- Perineal massage during second stage of labour[86, 153]
**Perineal pain**<br>CNGOF (2015)[86]<br>NICE (2021)[140]<br>WHO (2022)[112]	**Recommended**<br>- Assessing women for any perineal pain at postnatal contacts[157] using a validated pain scale to monitor perineal pain[140]<br>- Discuss perineal pain management options with woman (with consideration of breastfeeding)[140], and management by: paracetamol[86], oral NSAID therapy[86], ice packs or cold pads[86],<br>- Perineal pain can be associated with symptoms of depression, long-term perineal pain, problems with daily functioning and psychosexual difficulties[140]
	**Not recommended**<br>- Local anesthetics or therapeutic ultrasound in absence of demonstrated analgesic efficiency[86]
**Chronic pain**<br>EAU (2022)[158]	**Recommended**<br>- Performing history & examination to rule our treatable causes[158], and conducting investigations for possible causes (and to exclude disease-associated pelvic pain)[158]<br>- Assessing functional, emotional, behavioural, sexual and other quality of life issues[158]<br>- Ensuring a multidisciplinary care team is avaialble[158] and this team has required knowledge[158]. Involvement of mental health professionals if drug abuse is suspected[158]<br>- Appropriate use of opioids, in consultation with appropriate expertise[158]
**Placenta previa or accreta**<br>IS-AIP (2019)[131]<br>RCOG (2018)[128]<br>SOGC (2019)[132]<br>SOGC (2020)[133]	**Recommended**<br>Clinical assessment<br>- Identifying that women with previous CS, presence of low-lying placenta or placenta previa are at risk of PAS[128]<br>- Diagnosis of AIP/PAS after vaginal delivery requiring manual removal of placenta and the opinion of a senior, experienced obstetrician[131]<br>Counselling women on:<br>- Choice of anesthesia during CS for PAS[128]; and early ultrasound for future pregnancies following PAS[132]<br>- Risks of preterm birth and hemorrhage when low-lying placenta or placenta previa is present[128]<br>- Indications for urgent hospital attendance during 3rd trimester when low-lying placenta or placenta previa is present[128]<br>Screening:<br>- Mid-pregnancy fetal anomaly scan includes placental localization, so women with placenta previa or low-lying placenta are identifed[128]<br>- Women with previous CS and anterior low-lying placenta or placenta previa on anomaly scan are screened for PAS[128]<br>For women with Placenta Accreta Spectrum (PAS):<br>- Cervical cerclage[133]

Commented [JV1]: I think these are not recommended?

	- Accuracy of ultrasound by skilled provider for diagnosis[128]<br>- MRI to assess invasion[128]<br>- Multidisciplinary care team[128]<br>- Choice of anesthesia[128]<br>- Antenatal corticosteroids prior to delivery[131] and timing of birth[131]<br>- Hospital protocol for managing PAS[128,132]<br>- Uterus preserving surgery when PAS is limited[128]<br>- CS hysterectomy with placenta in situ[128]; postoperative follow up of women with PAS where placenta is left in situ[128]<br>- Leaving placenta in situ and emergency hysterectomy in women with unsuspected PAS diagnosed after birth[128]<br>- Women who decline blood transfusion should be cared for in unit with interventional radiology service[128]<br>- Measurement of Hb[131]<br>- Expectant outpatient management[131]<br>In women with placenta previa/low lying placenta:<br>- Timing of diagnosis of placenta previa or low-lying placenta[133]<br>- Transvaginal ultrasound if placenta previa or low-lying placenta is present/suspected[128,133]<br>- Follow up ultrasounds at 32 weeks for women with low-lying placenta or previa identified at routine fetal anomaly scan[128]<br>- Antenatal corticosteroids[128,133]<br>- In-hospital management of women with placenta previa[133]<br>- Tocolysis for women with antepartum hemorrhage due to contractions, and placenta previa[128]<br>- CS for women with low-lying placenta[128,133]/placenta previa (performed by experienced operator[128]) and alerting senior staff if emergency arises[128]<br>- Trial of labour in women with low-lying placenta[133]<br>- Regional anesthesia[128]<br>- CS: Place of birth[128], mode and timing of CS[133]; vertical incision at CS for transverse lie[128]<br>- During CS: cell salvage[128]; hysterectomy for PPH management[128]; availability of rapid infusion and fluid warming devices for women with placenta previa or low-lying placenta[128]<br>- Coordinating care with hematology/blood bank and liaising with transfusion service[128]<br>- Counselling on indications for transfusion and hysterectomy[128]<br>- Assessing risk of VTE[128]<br>- Prevention and treatment of anaemia[128]<br>- Individualized care[128]<br>- Late preterm delivery in women with placenta accreta[128]<br>- Delaying delivery to assemble care team and resources if placenta percreta identified at elective repeat CS[128]<br>- Pre/intraoperative ultrasound for placental localization and guiding CS incision[128]<br>Health systems

	- Prenatal diagnosis of severe PAS to support minimally invasive surgery[132]<br>- Refer women with risk factors for PAS & anterior placenta previa for diagnostic imaging[132]<br>- Clinical protocols for managing symptomatic women[131]<br>- Specialist center referral[128,132]/antenatal admission[132], and birth in specialist, multidisciplinary care centre[128]
	**Not recommended**<br>- For PAS: leaving placenta in situ in women undergoing CS[128]; interventional radiology for routine management[128]<br>- For women with low-lying placenta or placenta previa: use of tocolytics to prolong gestation[128]<br>- For management of AIP/PAS: methotrexate[128],[131] use of ureteric stents[128], prophylactic balloon catheters[131], local resection in selected women with AIP/PAS[131]<br>- Use of cervical cerclage in women with low-lying placenta or placenta previa[128]<br>- Uterus preserving surgery in placenta percreta[128]
**Deep vein thrombosis**<br>CNGOF (2015)[86]<br>ESVS (2021)[135]<br>WHO (2013)[108]<br>ACOG (2018)[134]	**Recommended**<br>- Use thromboprophylaxis and stockings based on risk factors[86]<br>- Advising women on signs of VTE postpartum[135]<br>- Compression ultrasonography for diagnosis of new onset DVT[134]<br>- Test for coagulation disorders in women with history of thrombosis[134]<br>- Protocol on use of safe use of anticoagulants and neuraxial anaesthesia[134] and on risk assessment for VTE in pregnancy and postpartum[134]<br>- LMWH for prevention and treatment of VTE[134]<br>- Anticoagulants compatible with breastfeeding[134]<br>- Postpartum pneumatic compression devices[134]
	**Not recommended**<br>- Use of D-dimer & Wells score in pregnant women with suspected DVT[135]
**Sexual dysfunction**<br>CNGOF (2015)[87]<br>NICE (2021)[140]<br>WHO (2013)[136]<br>WHO (2017)[124]	**Recommended**<br>Clinical assessment: sexual history at postnatal contact[124,136,140]
	**Not recommended**<br>Pelvic floor rehabilitation for dyspareunia[87]

Table S3.2. Conditions related to Caesarean section, laparotomy, hysterectomy, uterine rupture repair

Condition/Guidelines	Intervention
**Wound complications**<br>CNGOF (2015)[86]<br>NICE (2021)[140]	**Recommended**<br>Clinical assessment: Postnatal assessment of women following CS for symptoms of wound infection[140]<br>Prevention: Antibiotics at CS to prevent infections[86]
**Pain/nerve injury**<br>ACOG (2021)[118]	**Recommended**<br>Management: Multimodal approach for postpartum pain management after CS[118]
**Placenta previa or accreta**<br>IS-AIP (2019)[131]<br>NICE (2021)[150]<br>SOGC (2019)[132]	**Recommended**<br>Counselling<br>- Post-hysterectomy: counselling of women choosing expectant management for AIP / PAS[131]; women with AIP/PAS on future risk of same condition and fertility preservation[131]; counselling women with AIP/PAS on future risk of same condition[131]<br>- Discussing birth-related care with women with suspected PAS[150]<br>Diagnostic procedures<br>- Diagnosis of AIP/PAS after laparotomy using a stepwise process[131]<br>- Doppler ultrasound at 32-34 weeks in women with previous CS, to identify PAS if low-lying placenta is confirmed[150]<br>- Further imaging in women where Doppler ultrasound suggests PAS[150]<br>Management<br>- For women with PAS (general): Triple-P procedure[132], timing of CS birth[132], classical CS and non-removal of placenta[132], tranexamic acid at CS[132], modified lithotomy position[132], postpartum uterotonics[132]<br>- For women with AIP/PAS (general): local resection (in selected cases only)[131], tranexamic acid[131] for bleeding, uterotonics for bleeding[131]<br>- For women with AIP/PAS related to hysterectomy: expectant management[131], surgical position at CS[131], therapeutic embolization for PPH[131], management of bleeding[131], ureteric stents, uterine preservation procedures[131], total hysterectomy in women with AIP/PAS[131]; arresting blood flow from internal iliac prior to hysterectomy[132]<br>- Caesarean section: interoperative uterine ultrasound to locate placenta[131]; CS birth for women with placenta praevia[150]; regional anesthesia for women with PAS[132]<br>Health systems<br>- Hospital protocol for massive transfusion[132] and managing PAS[150]<br>- Post-hysterectomy: multidisciplinary care and blood products available during CS birth for women with PAS[150]
	**Not recommended**<br>Management<br>- Post-hysterectomy women with AIP/PAS: use of ureteric stents[131], prophylactic uterine artery embolisation[131], local resection in selected women[131]

	- Post-laparotomy women with AIP/PAS: surgical approach[131]<br>- Post-caesarean section: prophylactic uterotonics in women with suspected AIP/PAS[131]
**Adverse effects on subsequent pregnancies**<br>AAFP (2015)[137]<br>NICE (2019)[152]<br>RCOG (2018)[128]<br>SOGC (2019)[138]	**Recommended**<br>Counselling:<br>- Post-CS: risks of perinatal mortality/morbidity with trial of labour[138], risks of placenta previa and PAS with additional CS[138], risks of uterine rupture with trial of labour[138], risks of other maternal complications[138], likelihood of vaginal birth[137,138] and risks of labour vs elective CS[138], risk of trial of labour for women with breech presentation or multiple pregnancy or <18 month interval[138], counselling on VBAC and referral to VBAC services[137]<br>- Women with previous CS planning a trial of labour on: likelihood of uterine rupture with induction[138] and likelihood of uterine rupture with use of oxytocin for induction or augmentation[138]<br>- Women with previous CS requesting elective CS on: non-medical indication on future risk of PAS[128]<br>- Women with previous CS who are in labour on: risks of uterine rupture and emergency CS[152] and risks of using oxytocin for delay in first and second stage[152]<br>- Women with previous LSCS closed with single layer on risk of uterine rupture with trial of labour[138]<br>Management<br>- Women with previous CS: informed consent process on risks and benefits of trial of labour[137,138]; obtaining surgical record to determine uterine incision and whether trial of labour can be offered[138]; trial of labour (and its indications[138]) in women performed in hospital able to perform emergency CS[137,138]; Use of ultrasonographic measurement of lower uterine segment to inform risks of trial of labour after CS[138]; trial of labour for women with previous classical CS or T incision[138]; immediate response and laparotomy for woman with previous CS with suspected uterine rupture[138]; supporting women's informed decision-making on VBAC[137]<br>- For women with previous CS planning trial of labour: use of Foley catheter for cervical ripening[138]; use of prostaglandin E1/E2 for induction[138]<br>- For women with previous CS undergoing trial of labour[138]: alerting birthing unit staff to presence of woman, continuous electronic fetal monitoring; use of induction[152]<br>Health systems<br>- For women with previous CS: hospital guidelines for VBAC[137]
	**Not recommended**<br>- For women with previous CS: use of induction and methods of induction in women undergoing trial of labour[137]; amniotomy for women in labour[152]

Table S3.3. Conditions related to episiotomy/perineal repair

Condition/Guidelines	Intervention
**Anal incontinence** SOGC (2015)[139]	Recommended<br>- Rectal examination for women with 3rd/4th perineal tears[139]<br>- Referral for pelvic floor physiotherapy[139]
**Pelvic floor disorders** CNGOF (2019)[88]	Recommended<br>Prevention<br>- Description of injuries and repair when OASIS is identified[88]; detailed report of extend of injuries, technique of repair, and material used[88]<br>- Episiotomy methods - position of incision[88]<br>- Suturing - episiotomies and second-degree tears[88], anal sphincter[88]<br>- Surgery - repair of OASIS[88]
	Not recommended<br>Prevention<br>- Indications for episiotomy - substantial stretching of the perineum[88]<br>- Episiotomy to reduce the risk of OASIS[88]<br>- Suturing - first degree tears[88]
**Wound complications** WHO (2018)[157]	Not recommended<br>- Routine antibiotic prophylaxis after episiotomy[157]
**Poorly healed perineum** CNGOF (2015)[86] NICE (2017)[109] SOGC (2015)[139]	Recommended<br>Prevention<br>- Policy of restricted episiotomy[139]<br>- Mediolateral episiotomy technique[139]<br>- Intravenous antibiotics following OASIS repair[139]<br>Counselling<br>- Perineum hygiene advice for women with episiotomy or perineal tear[86,109]<br>Management<br>- Timing of perineal repair[139]<br>- Laxatives[139] and follow up[139] after OASIS repair<br>- Antibiotic therapy for 3rd/4th degree tear[86]

Table S3.4. Conditions related to operative vaginal birth

Condition/Guidelines	Intervention
**Pelvic floor disorders**<br>CNGOF (2019)[88]<br>WHO (2021)[92]	**Recommended**<br>Prevention<br>- Reducing the need for assisted vaginal birth[92] and primary CS[92]<br>- Assisted vaginal birth: training[92], episiotomy[92], conditions for safe and effective assisted vaginal birth[92]; choice of instrument determined by physician[92]. Debrief immediately following attempted or successful assisted vaginal birth[92]; choice of instrument determined by physician[92], spontaneous vaginal birth after assisted vaginal birth[92]<br>- Trial of vaginal birth[92]<br>- Episiotomy to reduce the risk of OASIS[88]<br>- Operative vaginal delivery involving several instruments[88], withdraw forceps or spatula if used just before cephalic deflexion[88]
	**Not recommended**<br>- Prevention: Choice of instrument for assisted vaginal birth[92]

References

1. Shea BJ, Reeves BC, Wells G, et al. AMSTAR 2: a critical appraisal tool for systematic reviews that include randomised or non-randomised studies of healthcare interventions, or both. *BMJ* 2017; **358**: j4008.
2. Banaei M, Kariman N, Ozgoli G, et al. Prevalence of postpartum dyspareunia: A systematic review and meta-analysis. *Int J Gynaecol Obstet* 2021; **153**(1): 14-24.
3. Baradaran K. Risk of Uterine Rupture with Vaginal Birth after Cesarean in Twin Gestations. *Obstet Gynecol Int* 2021; **2021**: 6693142.
4. Bij de Vaate AJ, van der Voet LF, Naji O, et al. Prevalence, potential risk factors for development and symptoms related to the presence of uterine niches following Cesarean section: systematic review. *Ultrasound in obstetrics & gynecology : the official journal of the International Society of Ultrasound in Obstetrics and Gynecology* 2014; **43(4)**: 372-82.
5. Blondon M, Casini A, Hoppe KK, Boehlen F, Righini M, Smith NL. Risks of Venous Thromboembolism After Cesarean Sections: A Meta-Analysis. *CHEST* 2016; **150**(3): 572-96.
6. Chen Y, Yang X, Guo C, et al. Prevalence of Post-Traumatic Stress Disorder Following Caesarean Section: A Systematic Review and Meta-Analysis. *Journal of Women's Health (15409996)* 2020; **29**(2): 200-9.
7. Chen Y, Geng X, Zhou H, et al. Systematic review and meta-analysis of evaluation of selective cesarean section in postpartum pelvic floor function recovery under perineal ultrasound. *Ann Palliat Med* 2022; **11**(2): 730-42.
8. Christopher S, McCullough J, Snodgrass SJ, Cook C. Predictive Risk Factors for First-Onset Lumbopelvic Pain in Postpartum Women: A Systematic Review. *Journal of Women's Health Physical Therapy* 2019; **43**(3): 127-35.
9. Cowgill KD, Bishop J, Norgaard AK, Rubens CE, Gravett MG. Obstetric fistula in low-resource countries: An under-valued and under-studied problem - systematic review of its incidence, prevalence, and association with stillbirth. *BMC Pregnancy and Childbirth* 2015; **15(1) (no pagination)**.
10. De Mucio B, Serruya S, Alemán A, Castellano G, Sosa CG. A systematic review and meta-analysis of cesarean delivery and other uterine surgery as risk factors for placenta accreta. *International Journal of Gynecology & Obstetrics* 2019; **147**(3): 281-91.
11. Dekel S, Stuebe C, Dishy G. Childbirth Induced Posttraumatic Stress Syndrome: A Systematic Review of Prevalence and Risk Factors. *Frontiers in psychology* 2017; **8**: 560.
12. Dennis CL, Falah-Hassani K, Shiri R. Prevalence of antenatal and postnatal anxiety: Systematic review and meta-analysis. *British Journal of Psychiatry* 2017; **210(5)**: 315-23.
13. Downes KL, Grantz KL, Shenassa ED. Maternal, Labor, Delivery, and Perinatal Outcomes Associated with Placental Abruption: A Systematic Review. *Am J Perinatol* 2017; **34**(10): 935-57.
14. Drake AL, Wagner A, Richardson B, John-Stewart G. Incident HIV during pregnancy and postpartum and risk of mother-to-child HIV transmission: a systematic review and meta-analysis. *PLoS medicine* 2014; **11**(2): e1001608.
15. Fawcett EJ, Fairbrother N, Cox ML, White IR, Fawcett JM. The Prevalence of Anxiety Disorders During Pregnancy and the Postpartum Period: A Multivariate Bayesian Meta-Analysis. *J Clin Psychiatry* 2019; **80**(4).
16. Frigerio M, Mastrolia SA, Spelzini F, Manodoro S, Yohay D, Weintraub AY. Long-term effects of episiotomy on urinary incontinence and pelvic organ prolapse: a systematic review. *Arch Gynecol Obstet* 2019; **299**(2): 317-25.
17. Gray TG, Vickers H, Jha S, Jones GL, Brown SR, Radley SC. A systematic review of non-invasive modalities used to identify women with anal incontinence symptoms after childbirth. *Int Urogynecol J* 2019; **30**(6): 869-79.
18. Hahn-Holbrook J, Cornwell-Hinrichs T, Anaya I. Economic and Health Predictors of National Postpartum Depression Prevalence: A Systematic Review, Meta-analysis, and Meta-Regression of 291 Studies from 56 Countries. *Frontiers in psychiatry* 2017; **8**: 248.
19. Hartmann K, Viswanathan M, Palmieri R, Gartlehner G, Thorp J, Jr., Lohr KN. Outcomes of routine episiotomy: a systematic review. *Jama* 2005; **293**(17): 2141-8.

20. Isogai T, Kamiya CA. Worldwide Incidence of Peripartum Cardiomyopathy and Overall Maternal Mortality. *Int Heart J* 2019; **60**(3): 503-11.
21. Kalra H, Tran T, Romero L, Chandra P, Fisher J. Burden of severe maternal peripartum mental disorders in low- and middle-income countries: a systematic review. *Archives of Women's Mental Health* 2022; **25(2)**: 267-75.
22. Keag OE, Norman JE, Stock SJ. Long-term risks and benefits associated with cesarean delivery for mother, baby, and subsequent pregnancies: Systematic review and meta-analysis. *PLoS Med* 2018; **15**(1): e1002494.
23. Korzeniewski R, Kiemle G, Slade P. Mothers' experiences of sex and sexual intimacy in the first postnatal year: a systematic review. *Sexual & Relationship Therapy* 2021; **36**(2/3): 219-37.
24. Liu X, Wang S, Wang G. Prevalence and Risk Factors of Postpartum Depression in Women: A Systematic Review and Meta-analysis. *Journal of clinical nursing* 2021; **08**.
25. Manresa M, Pereda A, Bataller E, Terre-Rull C, Ismail KM, Webb SS. Incidence of perineal pain and dyspareunia following spontaneous vaginal birth: a systematic review and meta-analysis. *International urogynecology journal* 2019; **30**(6): 853-68.
26. Marshall NE, Fu R, Guise JM. Impact of multiple cesarean deliveries on maternal morbidity: a systematic review. *American Journal of Obstetrics & Gynecology* 2011; **205**(3): 262.e1-8.
27. Meng K, Hu X, Peng X, Zhang Z. Incidence of venous thromboembolism during pregnancy and the puerperium: a systematic review and meta-analysis. *Journal of Maternal-Fetal & Neonatal Medicine* 2015; **28**(3): 245-53.
28. Moossdorff-Steinhauser HFA, Berghmans BCM, Spaanderman MEA, Bols EMJ. Prevalence, incidence and bothersomeness of urinary incontinence between 6 weeks and 1 year post-partum: a systematic review and meta-analysis. *Int Urogynecol J* 2021; **32**(7): 1675-93.
29. Nicholson WK, Robinson KA, Smallridge RC, Ladenson PW, Powe NR. Prevalence of postpartum thyroid dysfunction: a quantitative review. *Thyroid : official journal of the American Thyroid Association* 2006; **16**(6): 573-82.
30. Nielsen-Scott M, Fellmeth G, Opondo C, Alderdice F. Prevalence of perinatal anxiety in low- and middle-income countries: A systematic review and meta-analysis. *Journal of affective disorders* 2022; **306**: 71-9.
31. Nilsson C, Hessman E, Sjoblom H, et al. Definitions, measurements and prevalence of fear of childbirth: A systematic review. *BMC Pregnancy and Childbirth* 2018; **18(1) (no pagination)**.
32. O'Connell MA, Leahy-Warren P, Khashan AS, Kenny LC, O'Neill SM. Worldwide prevalence of tocophobia in pregnant women: systematic review and meta-analysis. *Acta Obstetricia et Gynecologica Scandinavica* 2017; **96**(8): 907-20.
33. Rider Sleutel M, True B, Webb J, Valdez E, Van Thi Tran M. Integrative Review of Lower Extremity Nerve Injury During Vaginal Birth. *JOGNN: Journal of Obstetric, Gynecologic & Neonatal Nursing* 2020; **49**(6): 507-24.
34. Shorey S, Chee CYI, Ng ED, Chan YH, Tam WWS, Chong YS. Prevalence and incidence of postpartum depression among healthy mothers: A systematic review and meta-analysis. *Journal of Psychiatric Research* 2018; **104**: 235-48.
35. Sideris M, McCaughey T, Hanrahan JG, et al. Risk of obstetric anal sphincter injuries (OASIS) and anal incontinence: A meta-analysis. *European Journal of Obstetrics and Gynecology and Reproductive Biology* 2020; **252**: 303-12.
36. Smeets CFA, Vergeldt TFM, Notten KJB, Martens FMJ, van Kuijk SMJ. Association between levator ani avulsion and urinary incontinence in women: A systematic review and meta-analysis. *Int J Gynaecol Obstet* 2021; **153**(1): 25-32.
37. Tähtinen RM, Cartwright R, Tsui JF, et al. Long-term Impact of Mode of Delivery on Stress Urinary Incontinence and Urgency Urinary Incontinence: A Systematic Review and Meta-analysis. *Eur Urol* 2016; **70**(1): 148-58.
38. Tulandi T, Cohen A. Emerging Manifestations of Cesarean Scar Defect in Reproductive-aged Women. *Journal of Minimally Invasive Gynecology* 2016; **23**(6): 893-902.
39. VanderKruik R, Barreix M, Chou D, et al. The global prevalence of postpartum psychosis: a systematic review. *BMC psychiatry* 2017; **17**(1): 272.
40. Wang K, Xu X, Jia G, Jiang H. Risk Factors for Postpartum Stress Urinary Incontinence: a Systematic Review and Meta-analysis. *Reprod Sci* 2020; **27**(12): 2129-45.

41. Wang Z, Liu J, Shuai H, et al. Mapping global prevalence of depression among postpartum women. *Translational Psychiatry* 2021; **11(1) (no pagination)**.
42. Wang Y, Liu H, Chen Y, Yu X, Zhang Y, Kong X. Prevalence and risk factors of chronic pain after cesarean section: A systematic review. [Chinese]. *Chinese Journal of Evidence-Based Medicine* 2021; **21(10)**: 1195-202.
43. Weibel S, Neubert K, Jelting Y, et al. Incidence and severity of chronic pain after caesarean section: A systematic review with meta-analysis. *European Journal of Anaesthesiology* 2016; **33(11)**: 853-65.
44. Wilson E, Woodd SL, Benova L. Incidence of and Risk Factors for Lactational Mastitis: A Systematic Review. *Journal of Human Lactation* 2020; **36**(4): 673-86.
45. Woody CA, Ferrari AJ, Siskind DJ, Whiteford HA, Harris MG. A systematic review and meta-regression of the prevalence and incidence of perinatal depression. *J Affect Disord* 2017; **219**: 86-92.
46. Yildiz PD, Ayers S, Phillips L. The prevalence of posttraumatic stress disorder in pregnancy and after birth: A systematic review and meta-analysis. *Journal of Affective Disorders* 2017; **208**: 634-45.
47. Yimer H, Woldie H. Incidence and Associated Factors of Chronic Pain After Caesarean Section: A Systematic Review. *Journal of Obstetrics and Gynaecology Canada* 2019; **41(6)**: 840-54.
48. Brouwers MC, Kho ME, Browman GP, et al. AGREE II: advancing guideline development, reporting, and evaluation in health care. *Prev Med* 2010; **51**(5): 421-4.
49. Maheu-Giroux M, Filippi V, Samadoulougou S, et al. Prevalence of symptoms of vaginal fistula in 19 sub-Saharan Africa countries: a meta-analysis of national household survey data. *Lancet Glob Health* 2015; **3**(5): e271-8.
50. Kiersten J, A. P. Incontinence data from the Demographic and Health Surveys: comparative analysis of a proxy measurement of vaginal fistula and recommendations for future population-based data collection. Calverton, Maryland, USA, 2008.
51. Cowgill KD, Bishop J, Norgaard AK, Rubens CE, Gravett MG. Obstetric fistula in low-resource countries: an under-valued and under-studied problem--systematic review of its incidence, prevalence, and association with stillbirth. *BMC Pregnancy Childbirth* 2015; **15**: 193.
52. Adler AJ, Ronsmans C, Calvert C, Filippi V. Estimating the prevalence of obstetric fistula: a systematic review and meta-analysis. *BMC Pregnancy Childbirth* 2013; **13**: 246.
53. Wu JM, Vaughan CP, Goode PS, et al. Prevalence and trends of symptomatic pelvic floor disorders in U.S. women. *Obstet Gynecol* 2014; **123**(1): 141-8.
54. Sideris M, McCaughey T, Hanrahan JG, et al. Risk of obstetric anal sphincter injuries (OASIS) and anal incontinence: A meta-analysis. *Eur J Obstet Gynecol Reprod Biol* 2020; **252**: 303-12.
55. Mascarenhas MN, Flaxman SR, Boerma T, Vanderpoel S, Stevens GA. National, regional, and global trends in infertility prevalence since 1990: a systematic analysis of 277 health surveys. *PLoS Med* 2012; **9**(12): e1001356.
56. Korzeniewski R KG, Slade P. Mothers' experiences of sex and sexual intimacy in the first postnatal year: a systematic review. *Sexual and Relationship Therapy* 2019; **36**(2-3): 219-37.
57. Manresa M, Pereda A, Bataller E, Terre-Rull C, Ismail KM, Webb SS. Incidence of perineal pain and dyspareunia following spontaneous vaginal birth: a systematic review and meta-analysis. *Int Urogynecol J* 2019; **30**(6): 853-68.
58. Liu X, Wang S, Wang G. Prevalence and Risk Factors of Postpartum Depression in Women: A Systematic Review and Meta-analysis. *J Clin Nurs* 2022; **31**(19-20): 2665-77.
59. Hahn-Holbrook J, Cornwell-Hinrichs T, Anaya I. Economic and Health Predictors of National Postpartum Depression Prevalence: A Systematic Review, Meta-analysis, and Meta-Regression of 291 Studies from 56 Countries. *Front Psychiatry* 2017; **8**: 248.
60. Shorey S, Chee CYI, Ng ED, Chan YH, Tam WWS, Chong YS. Prevalence and incidence of postpartum depression among healthy mothers: A systematic review and meta-analysis. *J Psychiatr Res* 2018; **104**: 235-48.
61. Wang Z, Liu J, Shuai H, et al. Mapping global prevalence of depression among postpartum women. *Transl Psychiatry* 2021; **11**(1): 543.

62. Yildiz PD, Ayers S, Phillips L. The prevalence of posttraumatic stress disorder in pregnancy and after birth: A systematic review and meta-analysis. *J Affect Disord* 2017; **208**: 634-45.
63. Dekel S, Stuebe C, Dishy G. Childbirth Induced Posttraumatic Stress Syndrome: A Systematic Review of Prevalence and Risk Factors. *Front Psychol* 2017; **8**: 560.
64. VanderKruik R, Barreix M, Chou D, Allen T, Say L, Cohen LS. The global prevalence of postpartum psychosis: a systematic review. *BMC Psychiatry* 2017; **17**(1): 272.
65. Kalra H, Tran T, Romero L, Chandra P, Fisher J. Burden of severe maternal peripartum mental disorders in low- and middle-income countries: a systematic review. *Arch Womens Ment Health* 2022; **25**(2): 267-75.
66. Nielsen-Scott M, Fellmeth G, Opondo C, Alderdice F. Prevalence of perinatal anxiety in low- and middle-income countries: A systematic review and meta-analysis. *J Affect Disord* 2022; **306**: 71-9.
67. Dennis CL, Falah-Hassani K, Shiri R. Prevalence of antenatal and postnatal anxiety: systematic review and meta-analysis. *Br J Psychiatry* 2017; **210**(5): 315-23.
68. Nilsson C, Hessman E, Sjöblom H, et al. Definitions, measurements and prevalence of fear of childbirth: a systematic review. *BMC Pregnancy Childbirth* 2018; **18**(1): 28.
69. O'Connell MA, Leahy-Warren P, Khashan AS, Kenny LC, O'Neill SM. Worldwide prevalence of tocophobia in pregnant women: systematic review and meta-analysis. *Acta Obstet Gynecol Scand* 2017; **96**(8): 907-20.
70. Ijaz SH, Jamal S, Minhas AMK, et al. Trends in Characteristics and Outcomes of Peripartum Cardiomyopathy Hospitalizations in the United States Between 2004 and 2018. *Am J Cardiol* 2022; **168**: 142-50.
71. Meng K, Hu X, Peng X, Zhang Z. Incidence of venous thromboembolism during pregnancy and the puerperium: a systematic review and meta-analysis. *J Matern Fetal Neonatal Med* 2015; **28**(3): 245-53.
72. Rider Sleutel M, True B, Webb J, Valdez E, Van Thi Tran M. Integrative Review of Lower Extremity Nerve Injury During Vaginal Birth. *J Obstet Gynecol Neonatal Nurs* 2020; **49**(6): 507-24.
73. Christopher S MJ, Snodgrass S.J., Cook C. Predictive Risk Factors for First-Onset Lumbopelvic Pain in Postpartum Women: A Systematic Review. *Journal of Women's Health Physical Therapy* 2049; **43**(3): 127-35.
74. Nicholson WK, Robinson KA, Smallridge RC, Ladenson PW, Powe NR. Prevalence of postpartum thyroid dysfunction: a quantitative review. *Thyroid* 2006; **16**(6): 573-82.
75. De Mucio B, Serruya S, Alemán A, Castellano G, Sosa CG. A systematic review and meta-analysis of cesarean delivery and other uterine surgery as risk factors for placenta accreta. *Int J Gynaecol Obstet* 2019; **147**(3): 281-91.
76. Vandenberghe G, Bloemenkamp K, Berlage S, et al. The International Network of Obstetric Survey Systems study of uterine rupture: a descriptive multi-country population-based study. *Bjog* 2019; **126**(3): 370-81.
77. Weibel S, Neubert K, Jelting Y, et al. Incidence and severity of chronic pain after caesarean section: A systematic review with meta-analysis. *Eur J Anaesthesiol* 2016; **33**(11): 853-65.
78. Tulandi T, Cohen A. Emerging Manifestations of Cesarean Scar Defect in Reproductive-aged Women. *J Minim Invasive Gynecol* 2016; **23**(6): 893-902.
79. Bij de Vaate AJ, van der Voet LF, Naji O, et al. Prevalence, potential risk factors for development and symptoms related to the presence of uterine niches following Cesarean section: systematic review. *Ultrasound Obstet Gynecol* 2014; **43**(4): 372-82.
80. Blondon M, Casini A, Hoppe KK, Boehlen F, Righini M, Smith NL. Risks of Venous Thromboembolism After Cesarean Sections: A Meta-Analysis. *Chest* 2016; **150**(3): 572-96.
81. Yimer H, Woldie H. Incidence and Associated Factors of Chronic Pain After Caesarean Section: A Systematic Review. *J Obstet Gynaecol Can* 2019; **41**(6): 840-54.
82. Wang Y LCY, Yu X., Zhang Y, Xiang. A systematic review of the prevalence and risk factors of chronic pain after cesarean section. *Chinese Journal of Evidence-Based Medicine* 2021; **21**(10): 1195-202.
83. Chen Y, Yang X, Guo C, et al. Prevalence of Post-Traumatic Stress Disorder Following Caesarean Section: A Systematic Review and Meta-Analysis. *J Womens Health (Larchmt)* 2020; **29**(2): 200-9.

84. Marshall NE, Fu R, Guise JM. Impact of multiple cesarean deliveries on maternal morbidity: a systematic review. *Am J Obstet Gynecol* 2011; **205**(3): 262.e1-8.
85. World Health Organization. WHO Recommendation on Duration of Bladder Catheterization After Surgical Repair of Simple Obstetric Urinary Fistula. WHO Recommendation on Duration of Bladder Catheterization After Surgical Repair of Simple Obstetric Urinary Fistula. Geneva: World Health Organization; 2018.
86. Simon EG, Laffon M. [Maternal care after vaginal delivery and management of complications in immediate post-partum--Guidelines for clinical practice]. *J Gynecol Obstet Biol Reprod (Paris)* 2015; **44**(10): 1101-10.
87. Deffieux X, Vieillefosse S, Billecocq S, et al. [Postpartum pelvic floor muscle training and abdominal rehabilitation: Guidelines]. *J Gynecol Obstet Biol Reprod (Paris)* 2015; **44**(10): 1141-6.
88. Ducarme G, Pizzoferrato AC, de Tayrac R, et al. Perineal prevention and protection in obstetrics: CNGOF clinical practice guidelines. *J Gynecol Obstet Hum Reprod* 2019; **48**(7): 455-60.
89. Baeßler K, Aigmüller T, Albrich S, et al. Diagnosis and Therapy of Female Pelvic Organ Prolapse. Guideline of the DGGG, SGGG and OEGGG (S2e-Level, AWMF Registry Number 015/006, April 2016). *Geburtshilfe Frauenheilkd* 2016; **76**(12): 1287-301.
90. National Institute for Health and Care Excellence (NICE). Urinary incontinence and pelvic organ prolapse in women: management. 2019. https://www.nice.org.uk/guidance/ng123 (accessed 23 October 2022 2022).
91. National Institute for Health and Care Excellence (NICE). Postnatal care. 2021. https://www.nice.org.uk/guidance/ng194/resources/postnatal-care-pdf-66142082148037 (accessed 9 December 2022).
92. National Institute for Health and Care Excellence (NICE). Pelvic floor dysfunction: prevention and non-surgical management. 2021. https://www.nice.org.uk/guidance/ng210 (accessed 23 October 2022 2022).
93. European Association of Urology. Urinary incontinence in adults. 2019. https://d56bochluxqnz.cloudfront.net/media/EAU-Guidelines-on-Urinary-Incontinence-2019.pdf (accessed 23 October 2022 2022).
94. Geoffrion R, Larouche M. Guideline No. 413: Surgical Management of Apical Pelvic Organ Prolapse in Women. *J Obstet Gynaecol Can* 2021; **43**(4): 511-23.e1.
95. Paquette IM, Varma MG, Kaiser AM, Steele SR, Rafferty JF. The American Society of Colon and Rectal Surgeons' Clinical Practice Guideline for the Treatment of Fecal Incontinence. *Dis Colon Rectum* 2015; **58**(7): 623-36.
96. World Health Organization. WHO recommendations on maternal and newborn care for a positive postnatal experience. Geneva, 2022.
97. Qaseem A, Dallas P, Forciea MA, Starkey M, Denberg TD, Shekelle P. Nonsurgical management of urinary incontinence in women: a clinical practice guideline from the American College of Physicians. *Ann Intern Med* 2014; **161**(6): 429-40.
98. Nambiar AK, Arlandis S, Bø K, et al. European Association of Urology Guidelines on the Diagnosis and Management of Female Non-neurogenic Lower Urinary Tract Symptoms. Part 1: Diagnostics, Overactive Bladder, Stress Urinary Incontinence, and Mixed Urinary Incontinence. *Eur Urol* 2022; **82**(1): 49-59.
99. O'Reilly N, Nelson H, Conry J, et al. Screening for Urinary Incontinence in Women: A Recommendation From the Women's Preventive Services Initiative (2018). *Ann Intern Med* 2018; **169**(5).
100. World Health Organization. WHO Recommendations on Postnatal Care of the Mother and Newborn. 2013. https://apps.who.int/iris/handle/10665/97603.
101. Austin M, Highet N. Mental Health Care in the Perinatal Period: Australian Clinical Practice Guideline. . 2017. https://cope.org.au/wp-content/uploads/2017/10/Final-COPE-Perinatal-Mental-Health-Guideline.pdf (accessed 23 October 2022).
102. Healthcare Improvement Scotland, Scottish Intercollegiate Guidelines Network. Management of perinatal mood disorders. 2012. https://www.sign.ac.uk/assets/sign127_update.pdf (accessed 23 October 2022).

103. National Institute for Health and Care Excellence (NICE). Antenatal and postnatal mental health: clinical management and service guidance. 2014. https://www.nice.org.uk/guidance/cg192 (accessed 23 October 2022).
104. Joffres M, Jaramillo A, Dickinson J, et al. Recommendations on screening for depression in adults. *Cmaj* 2013; **185**(9): 775-82.
105. Lang E, Colquhoun H, LeBlanc JC, et al. Recommendation on instrument-based screening for depression during pregnancy and the postpartum period. *Cmaj* 2022; **194**(28): E981-e9.
106. Centre of Perinatal Excellence. Mental Health Care in the Perinatal Period. 2017. https://cope.org.au/wp-content/uploads/2017/10/Final-COPE-Perinatal-Mental-Health-Guideline.pdf (accessed 23 October 2022 2022).
107. Healthcare Improvement Scotland and Scottish Intercollegiate Guidelines Network. Management of perinatal mood disorders. 2012. https://www.sign.ac.uk/assets/sign127_update.pdf (accessed 23 October 2022 2022).
108. National Institute for Health and Care Excellence (NICE). Antenatal and postnatal mental health: clinical management and service guidance. 2014. https://www.nice.org.uk/guidance/cg192 (accessed 23 October 2022 2022).
109. National Institute for Health and Care Excellence (NICE). Intrapartum care for healthy women and babies. 2017. https://www.nice.org.uk/guidance/cg190 (accessed 23 October 2022 2022).
110. Registered Nurses' Association of Ontario. Assessment and interventions for perinatal depression. 2018. https://rnao.ca/sites/rnao-ca/files/bpg/Perinatal_Depression_FINAL_web.pdf (accessed 23 October 2022).
111. World Health Organization. WHO recommendations on maternal health. 2017. https://www.who.int/publications/i/item/WHO-MCA-17.10 (accessed 23 October 2022).
112. World Health Organization. WHO recommendations on maternal and newborn care for a positive postnatal experience. WHO recommendations on maternal and newborn care for a positive postnatal experience. Geneva: World Health Organization; 2022.
113. The Royal Australian and New Zealand College of Psychiatrists. Clinical practice guideline for the treatment of panic disorder, social anxiety disorder and generalised anxiety disorder. 2018. https://www.ranzcp.org/files/resources/college_statements/clinician/cpg/anxiety-cpg.aspx (accessed 23 October 2022).
114. Gregory KD, Chelmow D, Nelson HD, et al. Screening for Anxiety in Adolescent and Adult Women: A Recommendation From the Women's Preventive Services Initiative. *Ann Intern Med* 2020; **173**(1): 48-56.
115. National Institute for Health and Care Excellence (NICE). Caesarean birth. 2021. https://www.nice.org.uk/guidance/ng192 (accessed 23 October 2022).
116. American College of Obstetricians and Gynecologists, Presidential Task Force on Pregnancy Heart Disease Committee. ACOG Practice Bulletin No. 212: Pregnancy and Heart Disease. *Obstet Gynecol* 2019; **133**(5): e320-e56.
117. National Institute for Health and Care Excellence (NICE). Intrapartum care for women with existing medical conditions or obstetric complications and their babies. 2019. https://www.nice.org.uk/guidance/ng121 (accessed 23 October 2022).
118. American College of Obstetricians and Gynecologists. Pharmacologic Stepwise Multimodal Approach for Postpartum Pain Management: ACOG Clinical Consensus No. 1. *Obstet Gynecol* 2021; **138**(3): 507-17.
119. Ross DS, Burch HB, Cooper DS, et al. 2016 American Thyroid Association Guidelines for Diagnosis and Management of Hyperthyroidism and Other Causes of Thyrotoxicosis. *Thyroid* 2016; **26**(10): 1343-421.
120. Alexander EK, Pearce EN, Brent GA, et al. 2017 Guidelines of the American Thyroid Association for the Diagnosis and Management of Thyroid Disease During Pregnancy and the Postpartum. *Thyroid* 2017; **27**(3): 315-89.
121. Maxwell C, Gaudet L, Cassir G, et al. Guideline No. 392-Pregnancy and Maternal Obesity Part 2: Team Planning for Delivery and Postpartum Care. *J Obstet Gynaecol Can* 2019; **41**(11): 1660-75.

122. de Ruiter A, Taylor GP, Clayden P, et al. British HIV Association guidelines for the management of HIV infection in pregnant women 2012 (2014 interim review). *HIV Med* 2014; **15 Suppl 4**: 1-77.
123. World Health Organization. Consolidated Guidelines on HIV Testing Services: 5Cs: Consent, Confidentiality, Counselling, Correct Results and Connection 2015. Consolidated Guidelines on HIV Testing Services: 5Cs: Consent, Confidentiality, Counselling, Correct Results and Connection 2015. Geneva: World Health Organization; 2015.
124. World Health Organization. WHO recommendations on maternal health. 2017. https://www.who.int/publications/i/item/WHO-MCA-17.10 (accessed 23 October 2022 2022).
125. World Health Organization. Intrapartum care for a positive childbirth experience. 2018. https://apps.who.int/iris/bitstream/handle/10665/260178/9789241550215-eng.pdf (accessed 23 October 2022).
126. Danish Society of Obstetrics and Gynecology (DSOG). Anaemia and iron deficiency in pregnancy and postpartum. 2016. http://www.nfog.org/files/guidelines/NFOG_guidelines_DEN_Anaemia%20in%20pregnancy%20and%20post%20partum_2016.pdf (accessed 22 October 2022).
127. World Health Organization. Iron supplementation in postpartum women. 2016. https://apps.who.int/iris/handle/10665/249242 (accessed 23 October 2022).
128. Jauniaux E, Alfirevic Z, Bhide AG, et al. Placenta Praevia and Placenta Accreta: Diagnosis and Management: Green-top Guideline No. 27a. *Bjog* 2019; **126**(1): e1-e48.
129. National Institute for Health and Care Excellence (NICE). Postnatal Care. 2021. https://www.nice.org.uk/guidance/ng194 (accessed 23 October 2022.
130. European Association of Urology. Chronic Pelvic Pain. 2022. https://d56bochluxqnz.cloudfront.net/documents/full-guideline/EAU-Guidelines-on-Chronic-Pelvic-Pain-2022_2022-03-29-084111_kpbq.pdf (accessed 23 October 2022).
131. Collins SL, Alemdar B, van Beekhuizen HJ, et al. Evidence-based guidelines for the management of abnormally invasive placenta: recommendations from the International Society for Abnormally Invasive Placenta. *Am J Obstet Gynecol* 2019; **220**(6): 511-26.
132. Hobson SR, Kingdom JC, Murji A, et al. No. 383-Screening, Diagnosis, and Management of Placenta Accreta Spectrum Disorders. *J Obstet Gynaecol Can* 2019; **41**(7): 1035-49.
133. Jain V, Bos H, Bujold E. Guideline No. 402: Diagnosis and Management of Placenta Previa. *J Obstet Gynaecol Can* 2020; **42**(7): 906-17.e1.
134. American College of Obstetricians and Gynecologists. ACOG Practice Bulletin No. 196: Thromboembolism in Pregnancy. *Obstet Gynecol* 2018; **132**(1): e1-e17.
135. Kakkos SK, Gohel M, Baekgaard N, et al. Editor's Choice - European Society for Vascular Surgery (ESVS) 2021 Clinical Practice Guidelines on the Management of Venous Thrombosis. *Eur J Vasc Endovasc Surg* 2021; **61**(1): 9-82.
136. World Health Organization. WHO Recommendations on Postnatal Care of the Mother and Newborn. WHO Recommendations on Postnatal Care of the Mother and Newborn. Geneva: World Health Organization; 2013.
137. Hauk L. Planning for Labor and Vaginal Birth After Cesarean Delivery: Guidelines from the AAFP. *Am Fam Physician* 2015; **91**(3): 197-8.
138. Dy J, DeMeester S, Lipworth H, Barrett J. No. 382-Trial of Labour After Caesarean. *J Obstet Gynaecol Can* 2019; **41**(7): 992-1011.
139. Harvey MA, Pierce M, Alter JE, et al. Obstetrical Anal Sphincter Injuries (OASIS): Prevention, Recognition, and Repair. *J Obstet Gynaecol Can* 2015; **37**(12): 1131-48.
140. National Institute for Health and Care Excellence (NICE). Postnatal care. Postnatal care. London: National Institute for Health and Care Excellence (NICE); 2021.
141. Collins CW, Winters JC. AUA/SUFU adult urodynamics guideline: a clinical review. *Urol Clin North Am* 2014; **41**(3): 353-62, vii.
142. Women's Preventive Services Initiative. Screening for Urinary Incontinence in Women: A Recommendation From the Women's Preventive Services Initiative. *Ann Intern Med* 2018; **169**(5).
143. Qaseem A, Dallas P, Forciea MA, Starkey M, Denberg TD, Shekelle P. Nonsurgical management of urinary incontinence in women: a clinical practice guideline from the American College of Physicians. *Ann Intern Med*

2014; **161**(6): 429-40.
144. Registered Nurses' Association of Ontario. Assessment and interventions for perinatal depression. 2018. https://rnao.ca/sites/rnao-ca/files/bpg/Perinatal_Depression_FINAL_web.pdf (accessed 23 October 2022 2022).
145. U.S. Preventive Services Task Force. Screening for Depression in Adults. 2016. https://www.uspreventiveservicestaskforce.org/uspstf/document/RecommendationStatementFinal/depression-in-adults-screening (accessed 23 October 2022 2022).
146. Ministry of Health Malaysia. Management of Major Depressive Disorder. 2019. https://www.moh.gov.my/moh/resources/Penerbitan/CPG/Psychiatry%20&%20Mental%20health/CPG_Management_of_MDD_(Second_Edition)_04092020.pdf (accessed 23 October 2022 2022).
147. Curry SJ, Krist AH, Owens DK, et al. Interventions to Prevent Perinatal Depression: US Preventive Services Task Force Recommendation Statement. *Jama* 2019; **321**(6): 580-7.
148. Austin MP, Middleton P, Reilly NM, Highet NJ. Detection and management of mood disorders in the maternity setting: the Australian Clinical Practice Guidelines. *Women Birth* 2013; **26**(1): 2-9.
149. The Royal Australian and New Zealand College of Psychiatrists. Clinical practice guideline for the treatment of panic disorder, social anxiety disorder and generalised anxiety disorder. 2018. https://www.ranzcp.org/files/resources/college_statements/clinician/cpg/anxiety-cpg.aspx (accessed 23 October 2022 2022).
150. National Institute for Health and Care Excellence (NICE). Caesarean birth. 2021. https://www.nice.org.uk/guidance/ng192 (accessed 23 October 2022 2022).
151. American College of Obstetricians and Gynecologists. ACOG Practice Bulletin No. 212: Pregnancy and Heart Disease. *Obstet Gynecol* 2019; **133**(5): e320-e56.
152. National Institute for Health and Care Excellence (NICE). Intrapartum care for women with existing medical conditions or obstetric complications and their babies. 2019. https://www.nice.org.uk/guidance/ng121 (accessed 23 October 2022 2022).
153. World Health Organization. Intrapartum care for a positive childbirth experience. 2018. https://apps.who.int/iris/bitstream/handle/10665/260178/9789241550215-eng.pdf (accessed 23 October 2022 2022).
154. Danish Society of Obstetrics and Gynecology (DSOG). Anaemia and iron deficiency in pregnancy and postpartum. 2016. http://www.nfog.org/files/guidelines/NFOG_guidelines_DEN_Anaemia%20in%20pregnancy%20and%20post%20partum_2016.pdf (accessed 22 October 2022 2022).
155. World Health Organization. Iron supplementation in postpartum women. 2016. https://apps.who.int/iris/handle/10665/249242 (accessed 23 October 2022 2022).
156. National Institute for Health and Care Excellence (NICE). Postnatal Care. 2021. https://www.nice.org.uk/guidance/ng194 (accessed 23 October 2022 2022).
157. World Health Organization. WHO recommendations: Intrapartum care for a positive childbirth experience. WHO recommendations: Intrapartum care for a positive childbirth experience. Geneva: World Health Organization; 2018.
158. European Association of Urology. Chronic Pelvic Pain. 2022. https://d56bochluxqnz.cloudfront.net/documents/full-guideline/EAU-Guidelines-on-Chronic-Pelvic-Pain-2022_2022-03-29-084111_kpbq.pdf (accessed 23 October 2022 2022).
